# TM-MC 2.0: an enhanced chemical database of medicinal materials in Northeast Asian traditional medicine

**DOI:** 10.1186/s12906-023-04331-y

**Published:** 2024-01-16

**Authors:** Sang-Kyun Kim, Myung-Ku Lee, Ho Jang, Jeong-Ju Lee, Sanghun Lee, Yunji Jang, Hyunchul Jang, Anna Kim

**Affiliations:** 1 KM Data Division, Korea Institute of Oriental Medicine, Daejeon, 34054 Republic of Korea; 2 Division of Biomedical Research, Korea Research Institute of Bioscience & Biotechnology, Daejeon, 34141 Republic of Korea

**Keywords:** Medicinal material, Chemical compound, Northeast Asian traditional medicine, Chromatography article

## Abstract

**Background:**

As chromatographic techniques have advanced, many articles that analyze the constituting compounds of medicinal materials have been published in relation to Northeast Asian traditional medicine, including traditional Chinese medicine (TCM). TM-MC was launched in 2015, providing information about the chemical compounds in medicinal materials from chromatographic articles in PubMed. Since 2015, through continuous curation efforts, we have now released TM-MC 2.0 with significant improvements to the quantity and quality of the data (https://tm-mc.kr).

**Description:**

TM-MC 2.0 contains 635 medicinal materials, 34,107 chemical compounds (21,306 identified and de-duplicated), 13,992 targets, 27,997 diseases, and 5,075 prescriptions (2,393 de-duplicated by name). The database provides the largest number of identified compounds for medicinal materials listed in the pharmacopoeia compared to all TCM databases. In particular, marker compounds of medicinal materials and many newly discovered compounds were added through the manual curation of recent chromatographic articles.

**Conclusion:**

TM-MC 2.0 provides the largest collection of information about the chemical compounds of the medicinal materials listed in the Korean, Chinese, and Japanese pharmacopoeias. Our database can be utilized for network pharmacology in traditional medicine and for the compound screening of medicinal materials for modern drug discovery.

**Supplementary Information:**

The online version contains supplementary material available at 10.1186/s12906-023-04331-y.

## Background

Traditional medicine in Northeast Asia has long been used to treat a wide range of diseases. In particular, China, Korea, and Japan have empirically proven numerous theories of traditional medicine and have demonstrated the efficacy of herbal medicines for thousands of years. In recent years, as modern medicine has advanced, much research has presented scientific evidence of the benefits of traditional medicine. Various analytical studies have identified the constituents of herbal medicines [1], and many studies have explored the pharmacological activities of herbal medicines [2, 3].

Since the early 2000s, several traditional Chinese medicine (TCM) databases have been made publicly available, providing information about prescriptions, herbal medicine ingredients, the gene targets of active ingredients, and the corresponding links to modern diseases. These include TCMSP [4], TCMID 2.0 [5], TCM-ID [6], and HIT 2.0 [7]. TCMSP 2.3 provides information about 502 herbs, 13,729 ingredients, 3,339 targets, and 867 diseases. TCMID 2.0 was updated from TCMID [8] in 2018 and includes information on 46,929 prescriptions, 8,159 herbs, 43,413 ingredients, 17,603 targets, and 4,633 diseases. TCMID mainly collected ingredients from the Encyclopedia of Traditional Chinese Medicines [9], TCM-ID, and TCM Database@Taiwan [10], and has information about a wide range of plants beyond herbs listed in the Chinese pharmacopoeia [11]. TCM-ID has been updated since it was built in 2006 and currently contains information about 7,443 prescriptions, 2,751 prescription components, and 7,085 ingredients. HIT 2.0 was updated from HIT [12] and provides a curated dataset that focuses on the targets of herbal ingredients sourced from TCM-ID.

Recently, expanded databases and analytical systems such as HERB [13] and TCM-Mesh [14] that integrate several TCM databases have been reported. HERB provides gene expression profiles from high-throughput experiments and information pertaining to targets and diseases for herbs and ingredients obtained by combining multiple TCM databases. The herbs and ingredients in HERB have been integrated from SymMap [15], TCMID 2.0, TCMSP, and TCM-ID and amount to 7,263 and 49,258, respectively. TCM-Mesh is a database and data-mining system for network pharmacology analyses of TCM prescriptions compiled by integrating multiple databases, including TCM Database@Taiwan and TCMID.

In 2015, we established TM-MC [16], which provides manually curated information on the chemical compounds of medicinal materials listed in Korean, Chinese, and Japanese pharmacopoeias from chromatography studies in PubMed. TM-MC contains 536 medicinal materials and 14,127 compounds extracted from nearly 4,000 articles, but lists only the names of compounds described in the referenced articles. Accordingly, it was necessary to identify the chemical structures of the compounds. Furthermore, many chromatography articles have been published since 2015, meaning that the curation of these articles was also necessary.

Therefore, in response to these requirements, we released TM-MC 2.0, which significantly improved the quantity and quality of the data. Table 1 presents an overview of the data in TM-MC and TM-MC 2.0. By manually curating even recently published articles, the numbers of medicinal materials and chemical compounds have increased and, in particular, the chemical structure of every compound has been identified. TM-MC 2.0 also includes new information about prescriptions, gene targets, modern diseases, and their associations, which can be utilized in network pharmacology research.

TM-MC 2.0 provides a comprehensive resource for systems pharmacology research in Northeast Asian traditional medicine through the manual curation of the latest chromatographic studies. While other TCM databases also provide a variety of resources, TM-MC 2.0 has an advantage in that it contains the largest amount of identified chemical data of medicinal materials listed in pharmacopoeias compared to other TCM databases.

**Table 1:** Data overview and data counts for TM-MC and TM-MC 2.0. The numbers in parentheses refer to the number of compounds and prescriptions de-duplicated

	Data sources	Number of TM-MC data	Number of TM-MC 2.0 data
Medicinal materials	Extracted from the Korean, Chinese, and Japanese pharmacopoeias	536	635
Chemical compounds	Manually extracted and curated from chromatographic articles in PubMed	14,127	34,107 (21,306)
Prescriptions	Two prescription textbooks and five internal medicine textbooks used at Korean medicine universities	0	5,075 (2,393)
Targets	Protein-chemical links for Homo sapiens species in STITCH v5.0	0	13,992
Diseases	Gene-disease associations in DisGeNET v7.0	0	27,997

## Construction and content

### Chemical compounds of medicinal materials

In our previous database, in order to construct information on the chemical compounds of medicinal materials, PubMed articles were searched using the names of medicinal materials listed in the Korean, Chinese, and Japanese pharmacopoeias [17, 18] and chromatographic articles were filtered with chromatography-related keywords. Then, Korean medicine experts manually extracted the names of the constituent compounds of the medicinal materials from the full text of the filtered articles. In TM-MC 2.0, using the same method used to create TM-MC, chromatographic articles of medicinal materials with PMID numbers up to 32 million in PubMed were searched. 45 medicinal materials were built up with PMID numbers up to 35 million (Supplementary Table S1). After manually curating 10,373 of the retrieved articles, TM-MC 2.0 provides 34,107 chemical compounds of 635 medicinal materials extracted from the articles. The 635 medicinal materials include 556 from the Chinese pharmacopoeia, 454 from the Korean pharmacopoeia, and 192 from the Japanese pharmacopoeia, with many medicinal materials listed in multiple pharmacopoeias. All compounds are accompanied by the PMID of the article as evidence of its extraction, with the corresponding chemical structures illustrated using ChemDraw (https://perkinelmerinformatics.com/products/research/chemdraw). Excluding 3,257 compounds that could not be identified and drawn due to typos or a lack of information in the article, TM-MC 2.0 provides data for 21,306 unique compounds. Duplicates were removed using InChIKey for each compound. All identified compounds were given a numerical unique TM-MC ID, and 9,544 compounds have duplicated IDs because they are synonyms of other compounds. SDF and JPG files of the chemical structures of individual compounds can be downloaded from the TM-MC 2.0 website.

### Identifiers and pharmacokinetic properties

In addition to the TM-MC ID, chemical identifiers such as InChIKey, SMILES, and the molecular formulae are given for all identified compounds. PubChem [19] compound IDs were also provided by matching using the InChIKey data. Of the 21,306 identified compounds present in TM-MC 2.0, 16,030 compounds exactly matched the InChIKey of the PubChem compounds and 18,317 compounds matched the first 14 characters of the InChIKey. Because compounds may generally have stereoisomers, only the first 14 characters of the InChIKey were used for the comparison without stereochemical information. The remaining 2,989 were identified as newly discovered compounds that were not searched for in PubChem.

TM-MC 2.0 also contains information on a variety of physicochemical properties, such as the molecular weight, logP, hydrogen bond acceptor count, hydrogen bond donor count, topological polar surface area, rotatable bond count, aromatic ring count, and structural alert count for screening compounds for pharmacological activity and drug discovery. These property values were obtained by the Calculators and Predictors of ChemAxon (https://chemaxon.com/calculators-and-predictors). InChIKey was computed by InChI 1.0.6 [20], drug likeness was calculated using the quantitative estimate of drug-likeness (QED) equation [21], and oral bioavailability was assessed using Veber’s rule [22].

### Targets and diseases of chemical compounds

Information on the targets of chemical compounds was retrieved from STITCH v5.0 [23], which provides a great many of interactions involving chemicals and proteins. In our database, only interactions for Homo sapiens were used. After STITCH compounds with the same first 14 characters of InChIKey of the TM-MC compounds were searched, targets mapped to these searched compounds were provided. We used only part of the InChIKey because STITCH does not distinguish between different stereoisomers [24, 25]. For this reason, using the full InChIKey would make many compounds in STITCH unsearchable.

In order to provide disease information related to the targets, the gene-disease associations obtained from DisGeNET v7.0 were used [26]. Given that DisGeNET identifies targets using Entrez Gene IDs but STITCH uses Ensembl IDs, the protein aliases in the STRING database [27] were used to map these two different IDs.

### Prescriptions containing medicinal materials

In Northeast Asian traditional medicine, various prescriptions consisting of multiple medicinal materials are commonly used to treat diseases rather than a single medicinal material. TM-MC 2.0 contains 5,075 prescriptions which include one or more of 635 medicinal materials. These prescriptions were extracted and curated manually from two prescription textbooks [28, 29] and five internal medicine textbooks [30–34] used at Korean medicine universities. In textbooks, the same prescription can have different medicinal materials depending on the symptoms it treats. Therefore, in TM-MC 2.0, there are multiple prescriptions with the same name, and after de-duplicating with the prescription name, there were 2,393 prescriptions remaining. The treatment symptom data will be supplemented in the future. Each prescription consists of medicinal materials and their corresponding processing methods and dosages, the referenced ancient literature, and the page number of the textbook from which they were extracted. In addition, if there was any modern medical research on prescriptions in PubMed, indications of such prescriptions described in the papers were curated and provided.

## Utility and discussion

### Website and use case

TM-MC 2.0 is available at https://tm-mc.kr. The browsing and search capabilities of our database are similar to those of the previous system, but there have been updates for some data fields that were added or changed. In TM-MC 2.0, a timeline menu has been added to show a time series trend of when the curated compounds first appeared in PubMed articles. Detailed instructions for using our database are described in the help menu on the website.

**Fig. 1:**
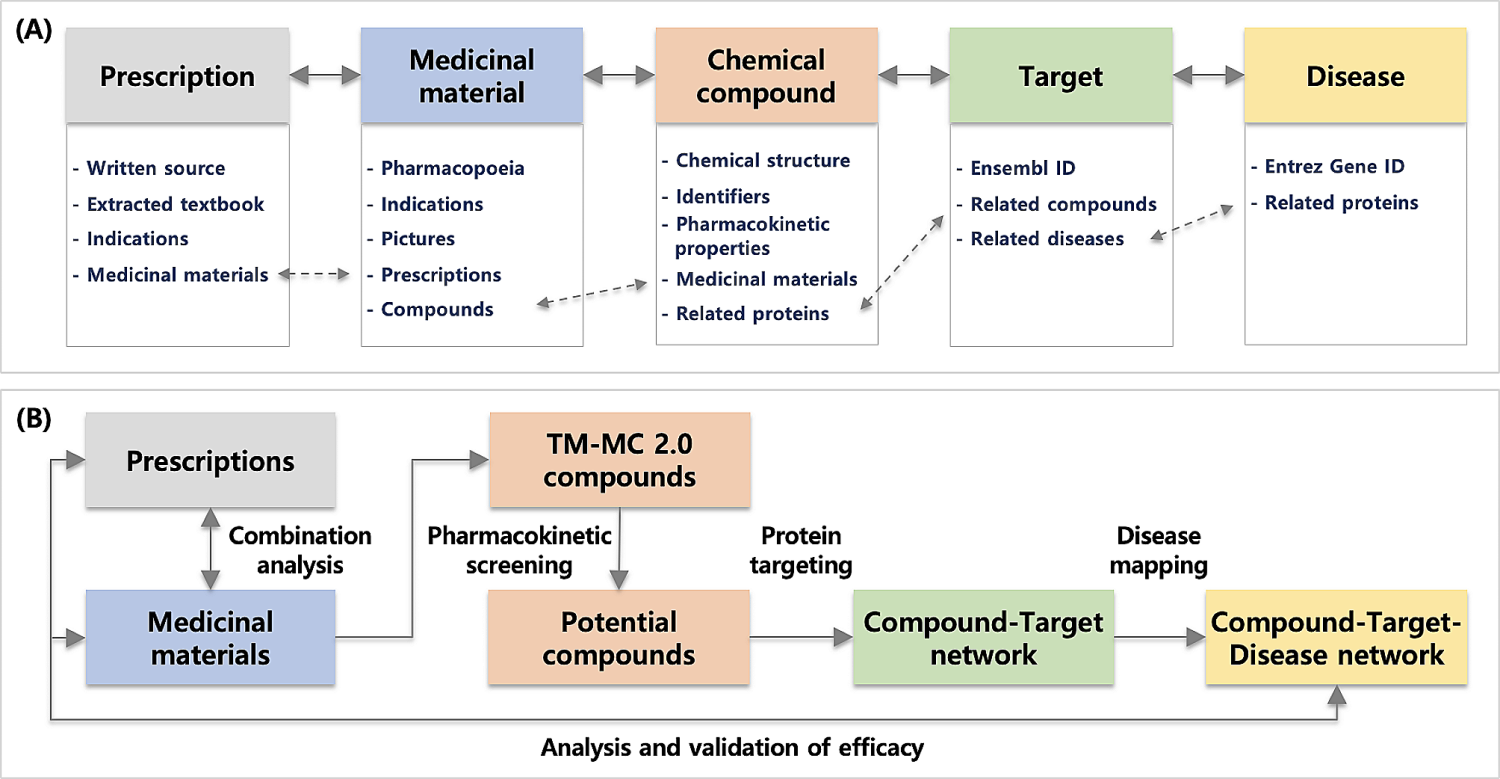
Data structures (**A**) and a use case (**B**) in TM-MC 2.0

Figure 1A describes the items in the TM-MC 2.0 data and the relationships between them. Figure 1B illustrates one use case of many scenarios using TM-MC 2.0 data. TM-MC 2.0 contains information on many medicinal materials and their constituent compounds. It also provides identifiers and pharmacokinetic properties of the compounds. In traditional medicine, prescriptions are used to treat diseases, and TM-MC 2.0 can be used to screen candidate active compounds for various combinations of medicinal materials. After protein targeting of potential components, a compound-target-disease network can be built, which can then be analyzed to predict the efficacy of herbal combinations or to conduct studies to validate the efficacy of existing prescriptions.

### Comparison with TCM databases

The most advantageous information in TM-MC 2.0 is the chemical compounds of medicinal materials. In order to describe how TM-MC 2.0 differs from other TCM databases, we compared the chemical information in TM-MC 2.0, TCMSP 2.3, and TCMID 2.0. Due to the different medicinal materials in the three databases, only compounds for the 387 medicinal materials common to all three databases were used for this comparison (Supplementary Table S2). In TCMSP, compounds with names ending in _qt were excluded from the comparison because _qt denotes a compound with no aglycone. Because the three databases may have different numbers of stereoisomers for a single compound, only the first 14 characters of InChIKey were used to include stereoisomers in the comparison. As TCMID does not provide InChIKeys for compounds, the InChIKey corresponding to the PubChem ID was used instead. Moreover, there are compounds with different InChIKeys but the same compound name, and these were mapped to the same compound to make the comparison less rigorous despite this difference.

**Fig. 2:**
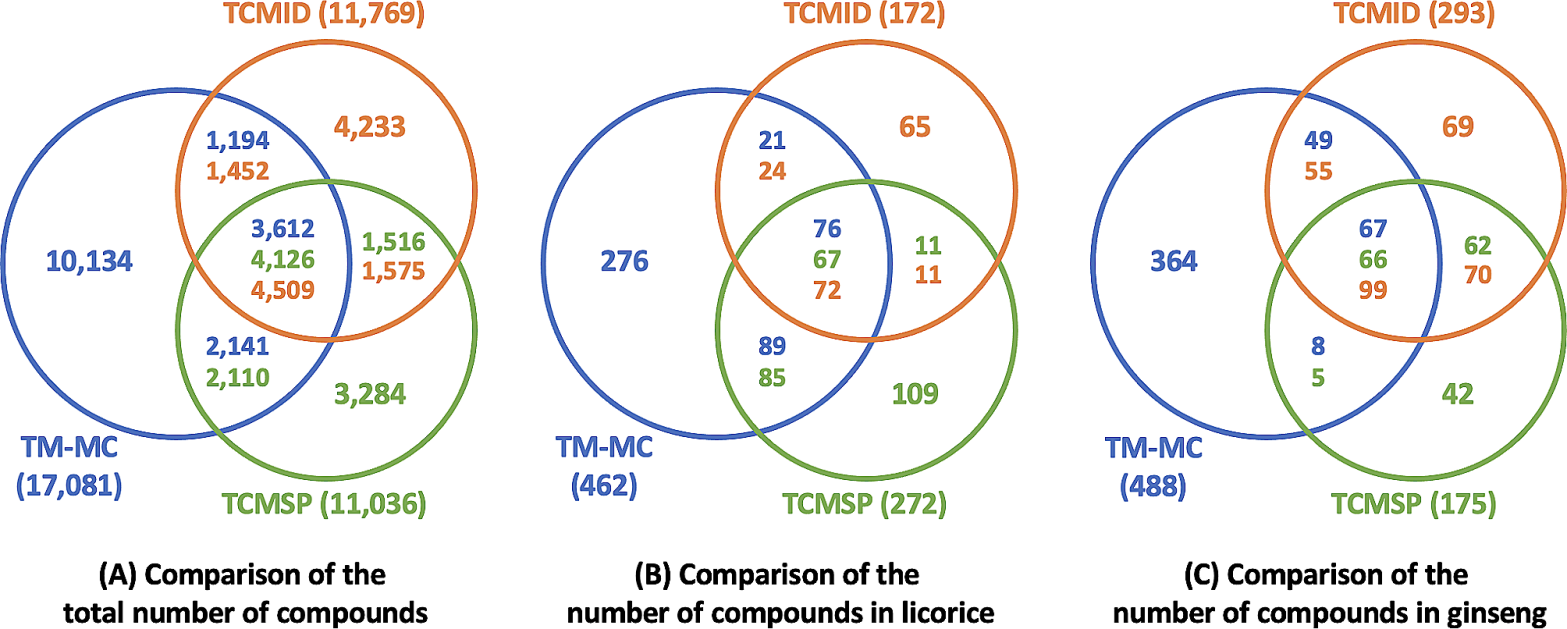
Comparisons of the number of compounds with the same InChIKeys or compound names in TM-MC 2.0, TCMSP 2.3, and TCMID 2.0. Blue, green, and orange denote TM-MC, TCMSP, and TCMID, respectively. The difference in the number of overlapping compounds present between two or three databases is due to the fact that the comparison was done using only the first 14 characters of InChIKey

Figure 2A shows the number of compounds matched by InChIKeys or compound names for 387 medicinal materials in the three databases. Figure 2B and C correspondingly present the numbers of compounds in licorice and ginseng, which are two of most popular herbal medicines. In particular, licorice is described as a case study in TCMSP and ginseng is used as a comparison example in TCM-Mesh. Supplementary Tables S3-S5 and S6-S8 list the compounds of licorice and ginseng in each database, respectively.

As illustrated in Fig. 2, TM-MC provides a much larger number of compounds than TCMSP and TCMID, but there are many compounds that are not common to two or three databases. Even between TCMSP and TCMID, many compounds do not overlap. It was noted in each paper that the compounds in TCMSP were gathered from literature searches and that those in TCMID were mainly collected from several books and databases. Therefore, while it is difficult to identify each compound in TCMSP and TCMID, it is certain that compounds that exist only in these databases are those that cannot be retrieved from chromatography articles in PubMed. Because TM-MC provides a literature citation for each compound, we checked the distribution of the newly discovered compounds in TM-MC according to the publication year in the PubMed articles, finding that more than half of the 10,134 compounds only present in TM-MC, 5,449 compounds, were curated from articles published after 2014, when TCMSP and TCMID were built. The number of newly discovered compounds per year and the corresponding lists can be confirmed in the timeline menu on the TM-MC homepage.

In order to compare the quality as well as quantity of the chemical data, we compared how many of the marker compounds listed in the Chinese pharmacopoeia were included in each database. The three databases have 239 medicinal materials in common with 350 marker compounds (Supplementary Table S9). Of the 350 marker compounds, only nine compounds could not be found in TM-MC, while TCMSP and TCMID did not have 63 and 55 compounds, respectively. This indicates that TM-MC 2.0 provides more comprehensive information about the compounds of medicinal materials and not merely a high number of compounds.

In recent years, many chromatographic articles have been published on the compounds of medicinal materials. TM-MC 2.0 has an advantage over other TCM databases in that it contains many new compounds that do not exist in previous databases. It also provides pharmacokinetic properties and links to targets for new compounds, which can help those involved in network pharmacology analyses and compound screening for drug discovery.

TM-MC 2.0 has a large number of compounds of medicinal materials, but for some medicinal materials with fewer articles found in PubMed, there may be less compound information than other TCM databases, as Chinese journals have published articles on the constituents of these medicinal materials, and TCM databases contain this information. However, as PubMed is continually updated and newer articles reference the results of Chinese articles, the chemical information in TM-MC 2.0 will increase.

### Usefulness and future plans

Recently, many databases have been proposed in Asian traditional medicine, including TCM, containing medicinal materials, compounds, targets, diseases, and their relationships. TM-MC 2.0 in particular provides the largest collection of information about identified compounds of medicinal materials listed in the pharmacopoeia as well as many new compounds not found in other TCM databases. We have curated articles with PMID numbers up to 32 million in PubMed, and for 45 medicinal materials, articles with PMID numbers up to 35 million have been added to TM-MC 2.0. As we work on curation, articles continue to be published, and we will therefore continue to curate the articles and update our database hereafter. We plan to upload the updated database to the TM-MC 2.0 homepage once a month.

TM-MC 2.0 contains many newly discovered compounds of medicinal materials, but they are not well-known active compounds. Individual medicinal materials generally contain tens to hundreds of compounds, and multiple compounds are known to exert synergistic therapeutic effects on multiple targets in traditional medicine. Therefore, in order to analyze the pharmacological activities of medicinal materials, it is desirable to screen all possible compounds rather than only major compounds. TM-MC 2.0 can be utilized for this purpose.

Compared to other popular chemical databases such as PubChem, TM-MC 2.0 does not have much information about individual compounds for drug screening. However, in that we have the chemical structure of each compound, we will attempt to use this feature to provide additional physicochemical properties and additional useful information such as safety and toxicity data.

Our database also provides prescriptions, the pharmacokinetic properties of compounds, compound-target associations and target-disease associations for large-scale pharmacological analyses, but the information about targets and diseases is not curated manually such as in HERB or HIT 2.0, implying that there may be less information available for a pharmacological analysis. However, because HERB and HIT 2.0 constructed their targets and diseases using the compounds retrieved from existing TCM databases, they do not provide sufficient information on targets and diseases for compounds newly discovered in recent chromatographic research on medicinal materials. Therefore, in the future, efforts should be made to explore the associations among these new compounds and targets or diseases, and we plan to improve our database by uncovering these associations.

## Conclusions

TM-MC 2.0 provides the largest collection of information about the chemical compounds of medicinal materials listed in the Korean, Chinese, and Japanese pharmacopoeias. The information on the chemical compounds of medicinal materials was manually extracted and curated from chromatographic articles in PubMed. All compounds have been identified and de-duplicated, and they are provided with their corresponding identifiers and pharmacokinetic properties. TM-MC 2.0 also includes new information on prescriptions, gene targets, modern diseases, and their associations. Our database can be utilized for network pharmacology in traditional medicine and for the compound screening of medicinal materials for modern drug discovery.

### Electronic supplementary material

Below is the link to the electronic supplementary material.

Supplementary Material 1

## Data Availability

All data is freely available at https://tm-mc.kr.

## Abbreviations

HERB: high-throughput experiment- and reference-guided database of TCM
InChIKey: International Chemical Identifier Key
PMID: PubMed identifier
QED: quantitative estimate of drug-likeness
SDF: structure-data format
SMILES: Simplified Molecular Input Line Entry System
TCM: traditional Chinese medicine
TCMID: Traditional Chinese Medicine Integrated Database
TCM-ID: Traditional Chinese Medicine Information Database
HIT: Herbal Ingredients’ Targets Platform
TCMSP: traditional Chinese medicine systems pharmacology database and analysis platform
TM-MC: database of medicinal materials and chemical compounds in Northeast Asian traditional medicine
