# Supplementary material for: TM-MC 2.0: an enhanced chemical database of medicinal materials in Northeast Asian traditional medicine
Source: BMC Complement Med Ther. 2024 Jan 16;24:40. doi: 10.1186/s12906-023-04331-y (PMC10790428; doi:10.1186/s12906-023-04331-y)
Supplement: Supplementary file 1 — Supplementary Material 1 [file 12906_2023_4331_MOESM1_ESM.docx]

**TM-MC 2.0: an enhanced chemical database of medicinal materials**

**in Northeast Asian traditional medicine**

| **No** | **Table caption** | **Page** |
| --- | --- | --- |
| **Table S1** | **List of 45 medicinal materials built up to articles with 35 million PMIDs and their names in Korean, Chinese, and Japanese pharmacopoeias** | **2** |
| **Table S2** | **List of 387 medicinal materials common to TM-MC, TCMSP, and TCMID** | **4** |
| **Table S3** | **List of licorice compounds in TM-MC and their existence in TCMSP and TCMID** | **14** |
| **Table S4** | **List of licorice compounds in TCMSP and their existence in TM-MC and TCMID** | **26** |
| **Table S5** | **List of licorice compounds in TCMID and their existence in TM-MC and TCMSP** | **34** |
| **Table S6** | **List of ginseng compounds in TM-MC and their existence in TCMSP and TCMID** | **39** |
| **Table S7** | **List of ginseng compounds in TCMSP and their existence in TM-MC and TCMID** | **52** |
| **Table S8** | **List of ginseng compounds in TCMID and their existence in TM-MC and TCMSP** | **57** |
| **Table S9** | **List of marker compounds from Chinese pharmacopoeia and their existence in TM-MC, TCMSP, and TCMID** | **65** |

Table S1 List of 45 medicinal materials built up to articles with 35 million PMIDs and their names in Korean, Chinese, and Japanese pharmacopoeias.

|  | Latin | Korean | Chinese | Japanese |
| --- | --- | --- | --- | --- |
| 1 | Achyranthis Radix | 우슬 | 牛膝 | ゴシツ |
| 2 | Acori Graminei Rhizoma | 석창포 | 石菖蒲 | セキショウコン |
| 3 | Actinidiae Fructus | 목천료 |  |  |
| 4 | Akebiae Fructus | 목통 | 预知子 |  |
| 5 | Akebiae Fructus | 예지자 | 预知子 |  |
| 6 | Artemisiae Annuae Herba | 청호 | 青蒿 |  |
| 7 | Boehmeriae Radix | 저마근 | 苎麻根 |  |
| 8 | Brassicae Semen | 개자 | 芥子 |  |
| 9 | Cannabis Semen | 마인 | 火麻仁 | マシニン |
| 10 | Centellae Herba |  | 积雪草 |  |
| 11 | Chelidonii Herba | 백굴채 | 白屈菜 |  |
| 12 | Codonopsis Pilosulae Radix | 당삼 | 党参 | トウジン |
| 13 | Commelinae Herba |  | 鸭跖草 |  |
| 14 | Cuscutae Semen | 토사자 | 菟丝子 |  |
| 15 | Daturae Folium | 만타라엽 |  |  |
| 16 | Carotae Fructus |  | 南鹤虱 |  |
| 17 | Euonymi Ramuli Suberalatum | 귀전우 | 鬼箭羽 |  |
| 18 | Foeniculi Fructus | 회향 | 小茴香 | ウイキョウ |
| 19 | Gastrodiae Rhizoma | 천마 | 天麻 | テンマ |
| 20 | Ginkgo Folium | 은행엽 | 银杏叶 |  |
| 21 | Ginkgonis Semen | 백과 | 白果 |  |
| 22 | Glycyrrhizae Radix et Rhizoma | 감초 | 甘草 | カンゾウ |
| 23 | Houttuyniae Herba | 어성초 | 魚腥草 | ジュウヤク |
| 24 | Hyperici Perforati Herba |  | 贯叶金丝桃 |  |
| 25 | Ilicis Cornutae Folium |  | 枸骨叶 |  |
| 26 | Lithospermi Radix | 자근 | 紫草 | シコン |
| 27 | Lonicerae Japonicae Flos | 금은화 | 金银花 | キンギンカ |
| 28 | Lonicerae Folium et Caulis | 인동 | 忍冬藤 | ニンドウ |
| 29 | Mori Radicis Cortex | 상백피 | 桑白皮 | ソウハクヒ |
| 30 | Mori Folium | 상엽 | 桑叶 |  |
| 31 | Mori Fructus | 상심자 | 桑椹 |  |
| 32 | Mori Ramulus | 상지 | 桑枝 |  |
| 33 | Ginseng Folium |  | 人参叶 |  |
| 34 | Ginseng Radix | 인삼 | 人参 | ニンジン |
| 35 | Picrasmae Lignum | 고목 | 苦木 | ニガキ |
| 36 | Persicae Semen | 도인 | 桃仁 | トウニン |
| 37 | Puerariae Radix | 갈근 | 葛根 | カッコン |
| 38 | Puerariae Flos | 갈화 |  |  |
| 39 | Galla Rhois | 오배자 | 五倍子 |  |
| 40 | Rosae Laevigatae Radix |  | 金樱根 |  |
| 41 | Rosae Rugosae Flos | 매괴화 | 玫瑰花 |  |
| 42 | Sedi Herba |  | 垂盆草 |  |
| 43 | Thlaspi Herba |  | 菥蓂 |  |
| 44 | Zizyphi Fructus | 대조 | 大枣 | タイソウ |
| 45 | Zosterae Herba | 해대 |  |  |

**Table S2 List of 387 medicinal materials common to TM-MC, TCMSP, and TCMID.**

| No | TM-MC | | | | TCMSP | TCMID |
| --- | --- | --- | --- | --- | --- | --- |
|  | Latin | Korean | Chinese | Japanese | Chinese | ID |
| 1 | Abri Herba | 계골초 | 鸡骨草 |  | 鸡骨草 | 5658 |
| 2 | Abutili Semen | 경마자 | 苘麻子 |  | 苘麻子 | 3632 |
| 3 | Achyranthis Radix | 우슬 | 牛膝 | ゴシツ | 牛膝 | 5736 |
| 4 | Aconiti Koreani Tuber | 백부자 | 白附子 关白附 |  | 白附子 | 3658 |
| 5 | Aconiti Kusnezoffii Tuber | 초오 | 草乌 |  | 草乌 | 1767 |
| 6 | Aconiti Lateralis Radix Preparata | 부자 | 附子 | ブシ | 附子 | 1173 |
| 7 | Aconiti Tuber | 천오 | 川乌 |  | 川乌 | 3194 |
| 8 | Acori Graminei Rhizoma | 석창포 | 石菖蒲 | セキショウコン | 石菖蒲 | 6161 |
| 9 | Adenophorae Radix | 사삼 | 南沙参 | シャジン | 南沙参 | 3279 |
| 10 | Aesculi Semen | 사라자 | 娑罗子 |  | 娑罗子 | 2895 |
| 11 | Agrimoniae Herba | 용아초 | 仙鹤草 |  | 仙鹤草 | 4918 |
| 12 | Ailanthi Radicis Cortex | 저백피 | 椿皮 |  | 椿皮 | 2300 |
| 13 | Akebiae Caulis | 목통 | 木通 | モクツウ | 木通 | 2276 |
| 14 | Akebiae Fructus | 예지자 | 预知子 |  | 预知子 | 2143 |
| 15 | Alismatis Rhizoma | 택사 | 泽泻 | タクシャ | 泽泻 | 2349 |
| 16 | Allii Bulbus | 대산 | 大蒜 |  | 大蒜 | 1766 |
| 17 | Allii Fistulosi Bulbus | 총백 |  |  | 葱白 | 1428 |
| 18 | Allii Macrostemi Bulbus | 해백 | 薤白 | ガイハク | 薤白 | 6621 |
| 19 | Allii Tuberosi Semen | 구자 | 韭菜子 |  | 韭菜子 | 2984, 4487 |
| 20 | Aloe | 노회 | 芦荟 | アロエ | 芦荟 | 3683 |
| 21 | Alpiniae Officinari Rhizoma | 고량강 | 高良姜 | リョウキョウ | 高良姜 | 5790 |
| 22 | Amomi Fructus | 사인 | 砂仁 | シュクシャ | 砂仁 | 3244, 3795 |
| 23 | Amomi Fructus Rotundus | 백두구 | 豆蔻 |  | 豆蔻 | 5741, 7678 |
| 24 | Amomi Tsao-ko Fructus | 초과 | 草果 |  | 草果 | 1651 |
| 25 | Andrographis Herba | 천심련 | 穿心莲 |  | 穿心莲 | 5703 |
| 26 | Anemarrhenae Rhizoma | 지모 | 知母 | チモ | 知母 | 1304 |
| 27 | Anemones Raddeanae Rhizoma | 양두첨 | 两头尖 |  | 两头尖 | 2188 |
| 28 | Angelicae Dahuricae Radix | 백지 | 白芷 | ビャクシ | 白芷 | 7294 |
| 29 | Angelicae Gigantis Radix | 당귀 | 当归 |  | 当归 | 2538 |
| 30 | Aquilariae Lignum | 침향 | 沉香 | ジンコウ | 沉香 | 6726 |
| 31 | Arachidis Testa | 화생의 | 花生衣 |  | 花生衣 | 7373 |
| 32 | Araliae Continentalis Radix | 독활 | 独活 | ドクカツ | 独活 | 4449 |
| 33 | Arctii Fructus | 우방자 | 牛蒡子 | ゴボウシ | 牛蒡子 | 3673 |
| 34 | Ardisiae Japonicae Herba | 왜지차 | 矮地茶 |  | 矮地茶 | 7629 |
| 35 | Arecae Pericarpium | 대복피 | 大腹皮 | ダイフクヒ | 大腹皮 | 4986 |
| 36 | Arecae Semen | 빈랑자 | 槟榔 | ビンロウジ | 槟榔 | 2896 |
| 37 | Arisaematis Rhizoma | 천남성 | 天南星 | テンナンショウ | 天南星 | 3811 |
| 38 | Aristolochiae Fructus | 마두령 | 马兜铃 |  | 马兜铃 | 2958 |
| 39 | Aristolochiae Herba | 천선등 | 天仙藤 |  | 天仙藤 | 2199 |
| 40 | Armeniacae Semen | 행인 | 苦杏仁 | キョウニン | 苦杏仁 | 5014, 7419 |
| 41 | Artemisiae Annuae Herba | 청호 | 青蒿 |  | 青蒿 | 7616 |
| 42 | Artemisiae Argyi Folium | 애엽 | 艾叶 | ガイヨウ | 艾叶 | 6218 |
| 43 | Artemisiae Capillaris Herba | 인진호 | 茵陈 | インチンコウ | 茵陈 | 3360, 4413 |
| 44 | Asiasari Radix et Rhizoma | 세신 | 细辛 | サイシン | 细辛 | 3947 |
| 45 | Asparagi Tuber | 천문동 | 天冬 | テンモンドウ | 天冬 | 1110, 1369 |
| 46 | Asteris Radix et Rhizoma | 자완 | 紫菀 | シオン | 紫菀 | 1401 |
| 47 | Astragali Complanati Semen | 사원자 | 沙苑子 |  | 沙苑子 | 1702 |
| 48 | Astragali Radix | 황기 | 黄芪 | オウギ | 黄芪 | 6919 |
| 49 | Atractylodis Rhizoma | 창출 | 苍术 | ソウジュツ | 苍术 | 5258 |
| 50 | Atractylodis Rhizoma Alba | 백출 | 白术 | ビャクジュツ | 白术 | 7301 |
| 51 | Aucklandiae Radix | 목향 | 木香 | モッコウ | 木香 | 5151 |
| 52 | Aurantii Fructus Immaturus | 지각 | 枳壳 |  | 枳壳 | 3567, 5777 |
| 53 | Belamcandae Rhizoma | 사간 | 射干 |  | 射干 | 5207 |
| 54 | Benincasae Semen | 동과자 | 冬瓜子 | トウガシ | 冬瓜子 | 5264 |
| 55 | Benzoinum | 안식향 | 安息香 | アンソッコウ | 安息香 | 5347 |
| 56 | Bistortae Rhizoma | 권삼 | 拳参 |  | 拳参 | 4978 |
| 57 | Bletillae Rhizoma | 백급 | 白及 |  | 白及 | 2447 |
| 58 | Boehmeriae Radix | 저마근 | 苎麻根 |  | 苎麻根 | 3731 |
| 59 | Bolbostemmatis Rhizoma | 토패모 | 土贝母 |  | 土贝母 | 4735 |
| 60 | Bomeolum | 용뇌 | 天然冰片 |  | 冰片 | 1932 |
| 61 | Bovis Calculus | 우황 | 牛黄 | ゴオウ | 牛黄 | 4459 |
| 62 | Brassicae Semen | 개자 | 芥子 |  | 芥子 | 1399, 2909 |
| 63 | Breeae Herba | 소계 | 小蓟 |  | 小蓟 | 1801 |
| 64 | Bruceae Fructus | 아담자 | 鸦胆子 |  | 鸦胆子 | 5194 |
| 65 | Buddlejae Flos | 밀몽화 | 密蒙花 |  | 密蒙花 | 5755 |
| 66 | Bupleuri Radix | 시호 | 柴胡 | サイコ | 柴胡 | 1663, 3396 |
| 67 | Campsitis Flos | 능소화 | 凌霄花 |  | 凌霄花 | 3073 |
| 68 | Canavaliae Semen | 도두 | 刀豆 |  | 刀豆 | 7740 |
| 69 | Cannabis Semen | 마인 | 火麻仁 | マシニン | 火麻仁 | 6070 |
| 70 | Capsici Fructus | 고추 | 辣椒 | トウガラシ | 辣椒 | 4112 |
| 71 | Carotae Fructus | 남학슬 | 南鹤虱 |  | 南鹤虱 | 7164 |
| 72 | Carpesii Fructus | 학슬 | 鹤虱 |  | 鹤虱 | 3682 |
| 73 | Carthami Flos | 홍화 | 红花 | コウカ | 红花 | 2474 |
| 74 | Cassiae Semen | 결명자 | 决明子 | ケツメイシ | 决明子 | 1509 |
| 75 | Catechu | 아차 | 儿茶 |  | 儿茶 | 3402 |
| 76 | Celosiae Cristatae Flos | 계관화 | 鸡冠花 |  | 鸡冠花 | 2852 |
| 77 | Centellae Herba | 적설초 | 积雪草 |  | 积雪草 | 4738 |
| 78 | Centipedae Herba | 아불식초 | 鹅不食草 |  | 鹅不食草 | 1267 |
| 79 | Chaenomelis Fructus | 모과 | 木瓜 | モッカ | 木瓜 | 4889 |
| 80 | Chelidonii Herba | 백굴채 | 白屈菜 |  | 白屈菜 | 2785 |
| 81 | Choerospondiatis Fructus | 광조 | 广枣 |  | 广枣 | 1671 |
| 82 | Chrysanthemi Indici Flos | 감국 | 野菊花 |  | 野菊花 | 1050 |
| 83 | Chrysanthmi Flos | 국화 | 菊花 | キクカ | 菊花 | 7682 |
| 84 | Cicadidae Periostracum | 선퇴 | 蝉蜕 | センタイ | 蝉蜕 | 2179 |
| 85 | Cichorii Radix | 국거 | 菊苣 |  | 菊苣 | 5053 |
| 86 | Cimicifugae Rhizoma | 승마 | 升麻 | ショウマ | 升麻 | 3006 |
| 87 | Cinnamomi Cortex | 육계 | 肉桂 | ケイヒ | 肉桂 | 7524 |
| 88 | Cinnamomi Ramulus | 계지 | 桂枝 | ケイシ | 桂枝 | 3977 |
| 89 | Cirsii Herba | 대계 | 大蓟 |  | 大蓟 | 2152 |
| 90 | Cistanchis Herba | 육종용 | 肉苁蓉 | ニクジュヨウ | 肉苁蓉 | 1883 |
| 91 | Citri Grandis Exocarpium | 화귤홍 | 化橘红 |  | 化橘红 | 7145 |
| 92 | Citri Sarcodactylis Fructus | 불수 | 佛手 |  | 佛手 | 4634 |
| 93 | Citri Semen | 귤핵 | 橘核 |  | 橘核 | 4320 |
| 94 | Citri Unshius Pericarpium | 진피 | 陈皮 | チンピ | 陈皮 | 1849, 5295 |
| 95 | Citri Unshius Pericarpium Immaturus | 청피 | 青皮 | セイヒ | 青皮 | 6196 |
| 96 | Clematidis Armandii Caulis | 천목통 | 川木通 |  | 川木通 | 8009 |
| 97 | Clematidis Radix | 위령선 | 威灵仙 | イレイセン | 威灵仙 | 2623 |
| 98 | Clinopodii Herba | 단혈류 | 断血流 |  | 断血流 | 3711 |
| 99 | Cnidi Fructus | 사상자 | 蛇床子 | ジャショウシ | 蛇床子 | 7399 |
| 100 | Cnidii Rhizoma | 천궁 | 川芎 | センキュウ | 川芎 | 5926 |
| 101 | Codonopsis Pilosulae Radix | 당삼 | 党参 | トウジン | 党参 | 1345 |
| 102 | Coicis Semen | 의이인 | 薏苡仁 | ヨクイニン | 薏苡仁 | 2186 |
| 103 | Commelinae Herba | 압척초 | 鸭跖草 |  | 鸭跖草 | 7004 |
| 104 | Coptidis Rhizoma | 황련 | 黄连 | オウレン | 黄连 | 7648 |
| 105 | Cordyceps | 동충하초 | 冬虫夏草 |  | 冬虫夏草 | 6269 |
| 106 | Coriolus | 운지 | 云芝 |  | 云芝 | 1069 |
| 107 | Corni Fructus | 산수유 | 山茱萸 | サンシュユ | 山茱萸 | 6859 |
| 108 | Corydalis Bungeanae Herba | 고지정 | 苦地丁 |  | 苦地丁 | 1308 |
| 109 | Corydalis Decumbentis Rhizoma | 하천무 | 夏天无 |  | 夏天无 | 6350 |
| 110 | Corydalis Tuber | 현호색 | 延胡索 | エンゴサク | 延胡索 | 3993 |
| 111 | Crataegi Folium | 산사엽 | 山楂叶 |  | 山楂叶 | 1978 |
| 112 | Cremastrae Tuber | 산자고 | 山慈菇 |  | 山慈菇 | 7523 |
| 113 | Crocus | 번홍화 | 西红花 | サフラン | 西红花 | 1784 |
| 114 | Crotonis Semen | 파두 | 巴豆 |  | 巴豆 | 4179 |
| 115 | Cubebae Fructus | 필징가 | 荜澄茄 |  | 荜澄茄 | 4954, 7034 |
| 116 | Curculiginis Rhizoma | 선모 | 仙茅 |  | 仙茅 | 7589 |
| 117 | Curcumae Longae Rhizoma | 강황 | 姜黄 | ウコン | 姜黄 | 6033 |
| 118 | Curcumae Radix | 울금 | 郁金 |  | 郁金 | 4926 |
| 119 | Curcumae Rhizoma | 아출 | 莪术 片姜黄 | ガジュツ | 莪术 | 5869, 7438 |
| 120 | Cuscutae Semen | 토사자 | 菟丝子 |  | 菟丝子 | 5404 |
| 121 | Cyathulae Radix | 천우슬 | 川牛膝 |  | 川牛膝 | 3068 |
| 122 | Cynanchi Atrati Radix et Rhizoma | 백미 | 白薇 |  | 白薇 | 2226 |
| 123 | Cynanchi Paniculati Radix et Rhizoma | 서장경 | 徐长卿 |  | 徐长卿 | 6191 |
| 124 | Cynanchi Stauntonii Rhizoma | 백전 | 白前 |  | 白前 | 6108 |
| 125 | Cyperi Rhizoma | 향부자 | 香附 | コウブシ | 香附 | 7520 |
| 126 | Dalbergiae Odoriferae Lignum | 강향 | 降香 |  | 降香 | 7370 |
| 127 | Daturae Flos | 양금화 | 洋金花 |  | 洋金花 | 6237 |
| 128 | Dianthi Herba | 구맥 | 瞿麦 |  | 瞿麦 | 2683 |
| 129 | Dichroae Radix | 상산 | 常山 |  | 常山 | 7342 |
| 130 | Dictamni Radicis Cortex | 백선피 | 白鲜皮 |  | 白鲜皮 | 3445 |
| 131 | Dioscorea Bulbifera Rhizoma | 황약자 | 黄药子 |  | 黄药子 | 4200 |
| 132 | Dioscoreae Hypoglaucae Rhizoma | 분비해 | 粉萆薢 |  | 粉萆薢 | 4269, 4981 |
| 133 | Dioscoreae Nipponicae Rhizoma | 천산룡 | 穿山龙 |  | 穿山龙 | 3154 |
| 134 | Dioscoreae Rhizoma | 산약 | 山药 | サンヤク | 山药 | 6311 |
| 135 | Dioscoreae Spongiosae Rhizoma | 면비해 | 绵萆薢 |  | 绵萆薢 | 4939 |
| 136 | Dipsaci Radix | 속단 | 续断 |  | 续断 | 2428 |
| 137 | Dolichoris Semen | 백편두 | 白扁豆 | ヘンズ | 白扁豆 | 2519 |
| 138 | Drynariae Rhizoma | 골쇄보 | 骨碎补 |  | 骨碎补 | 4192 |
| 139 | Dryopteridis Crassirhizomatis Rhizoma | 관중 | 绵马贯众 |  | 贯众 | 2994 |
| 140 | Echinopsis Radix | 우주누로 | 禹州漏芦 |  | 禹州漏芦 | 5643 |
| 141 | Ecliptae Herba | 한련초 | 墨旱莲 |  | 墨旱莲 | 4879, 6955 |
| 142 | Elsholtziae Herba | 향유 | 香薷 |  | 香薷 | 5284 |
| 143 | Ephedrae Herba | 마황 | 麻黄 | マオウ | 麻黄 | 1660 |
| 144 | Ephedrae Radix | 마황근 | 麻黄根 |  | 麻黄根 | 3075 |
| 145 | Epimedii Herba | 음양곽 | 淫羊藿 巫山淫羊藿 | インヨウカク | 淫羊藿 | 2982 |
| 146 | Equiseti Herba | 목적 | 木贼 |  | 木贼 | 6886 |
| 147 | Eriobotryae Folium | 비파엽 | 枇杷叶 | ビワヨウ | 枇杷叶 | 7019 |
| 148 | Eriocauli Flos | 곡정초 | 谷精草 |  | 谷精草 | 1881 |
| 149 | Erycibae Caulis | 정공등 | 丁公藤 |  | 丁公藤 | 3245 |
| 150 | Eucommiae Cortex | 두충 | 杜仲 | トチュウ | 杜仲 | 6430 |
| 151 | Eucommiae Folium | 두충엽 | 杜仲叶 |  | 杜仲叶 | 5621 |
| 152 | Euonymi Ramuli Suberalatum | 귀전우 | 鬼箭羽 |  | 鬼箭羽 | 7075 |
| 153 | Euphorbiae Humifusae Herba | 지금초 | 地锦草 |  | 地锦草 | 7880 |
| 154 | Euphorbiae Kansui Radix | 감수 | 甘遂 |  | 甘遂 | 3336 |
| 155 | Euphorbiae Lathyridis Semen | 속수자 | 千金子 |  | 千金子 | 1106 |
| 156 | Euphorbiae Pekinensis Radix | 대극 | 京大戟 |  | 京大戟 | 2153, 7506 |
| 157 | Euryales Semen | 검인 | 芡实 |  | 芡实 | 6510 |
| 158 | Evodiae Fructus | 오수유 | 吴茱萸 | ゴシュユ | 吴茱萸 | 7935 |
| 159 | Fagopyri Dibotryis Rhizoma | 금교맥 | 金荞麦 |  | 金荞麦 | 3272, 5271 |
| 160 | Foeniculi Fructus | 회향 | 小茴香 | ウイキョウ | 小茴香 | 4943, 6331 |
| 161 | Forsythiae Fructus | 연교 | 连翘 | レンギョウ | 连翘 | 4518 |
| 162 | Fraxini Cortex | 진피 | 秦皮 |  | 秦皮 | 6789 |
| 163 | Fritillariae Cirrhosae Bulbus | 천패모 | 川贝母 |  | 川贝母 | 6371 |
| 164 | Fritillariae Pallidiflorae Bulbus | 이패모 | 伊贝母 |  | 伊贝母 | 2514 |
| 165 | Fritillariae Thunbergii Bulbus | 절패모 | 浙贝母 |  | 浙贝母 | 5615 |
| 166 | Fritillariae Ussuriensis Bulbus | 평패모 | 平贝母 |  | 平贝母 | 7995 |
| 167 | Galla Rhois | 오배자 | 五倍子 |  | 五倍子 | 6584 |
| 168 | Ganoderma | 영지 | 灵芝 |  | 灵芝 | 1006 |
| 169 | Gardeniae Fructus | 치자 | 栀子 | サンシシ | 栀子 | 2221 |
| 170 | Genkwae Flos | 원화 | 芫花 |  | 芫花 | 7504 |
| 171 | Gentianae Macrophyllae Radix | 진교 | 秦艽 | ジンギョウ | 秦艽 | 6353 |
| 172 | Gentianae Scabrae Radix et Rhizoma | 용담 | 龙胆 | リュウタン | 龙胆 | 1746 |
| 173 | Geranii Herba | 현초 | 老鹳草 | ゲンノショウコ | 老鹳草 | 1592 |
| 174 | Ginkgo Folium | 은행엽 | 银杏叶 |  | 银杏叶 | 1670, 2738 |
| 175 | Ginkgonis Semen | 백과 | 白果 |  | 白果 | 7069 |
| 176 | Ginseng Folium | 인삼엽 | 人参叶 |  | 人参叶 | 7227 |
| 177 | Ginseng Radix | 인삼 | 人参 | ニンジン | 人参 | 3861 |
| 178 | Ginseng Radix Rubra | 홍삼 | 红参 | コウジン | 红参 | 8051 |
| 179 | Glechomae Herba | 연전초 | 连钱草 |  | 连钱草 | 1891 |
| 180 | Gleditsiae Fructus | 조협 | 猪牙皂 大皂角 |  | 猪牙皂 | 2582 |
| 181 | Gleditsiae Spina | 조각자 | 皂角刺 |  | 皂角刺 | 2751 |
| 182 | Glehniae Radix | 해방풍 | 北沙参 | ハマボウフウ | 北沙参 | 1328 |
| 183 | Glycine Semen Nigra | 흑두 | 黑豆 |  | 黑豆 | 4090 |
| 184 | Glycine Semen Preparata | 두시 | 淡豆豉 |  | 淡豆豉 | 1297, 2150 |
| 185 | Glycyrrhizae Radix et Rhizoma | 감초 | 甘草 | カンゾウ | 甘草 | 6801 |
| 186 | Granati Cortex | 석류피 | 石榴皮 |  | 石榴皮 | 8158 |
| 187 | Hedysari Radix | 홍기 | 红芪 | シンギ | 红芪 | 8028 |
| 188 | Helwingiae Medulla | 소통초 | 小通草 |  | 小通草 | 7567 |
| 189 | Hemerocallidis Radix et Rhizoma | 훤초근 |  |  | 萱草根 | 1887 |
| 190 | Hibisci Cortex | 목근피 |  |  | 川槿皮 | 2108 |
| 191 | Hippophae Fructus | 사극 | 沙棘 |  | 沙棘 | 6230 |
| 192 | Homalomenae Rhizoma | 천년건 | 千年健 |  | 千年健 | 5656 |
| 193 | Hordei Fructus Germinatus | 맥아 | 麦芽 | バクガ | 麦芽 | 7659 |
| 194 | Houttuyniae Herba | 어성초 | 鱼腥草 | ジュウヤク | 鱼腥草 | 3847 |
| 195 | Humuli Herba | 율초 | 葎草 |  | 葎草 | 5995 |
| 196 | Hyoscyami Semen | 천선자 | 天仙子 |  | 天仙子 | 2941 |
| 197 | Ilicis Chinensis Folium | 사계청 | 四季青 |  | 四季青 | 8168 |
| 198 | Ilicis Cornutae Folium | 구골엽 | 枸骨叶 |  | 枸骨叶 | 3895 |
| 199 | Illici Veri Fructus | 팔각회향 | 八角茴香 |  | 八角茴香 | 4150 |
| 200 | Illicii Cortex | 지풍피 | 地枫皮 |  | 地枫皮 | 6143 |
| 201 | Impatientis Caulis | 봉선투골초 | 鲜凤仙透骨草 |  | 凤仙透骨草 | 1686 |
| 202 | Impatientis Semen | 급성자 | 急性子 |  | 急性子 | 7644 |
| 203 | Imperatae Rhizoma | 모근 | 白茅根 | ボウコン | 白茅根 | 6014 |
| 204 | Indigo Pulverata Levis | 청대 | 青黛 |  | 青黛 | 7058 |
| 205 | Inulae Flos | 선복화 | 旋覆花 |  | 旋覆花 | 7446 |
| 206 | Inulae Herba | 금비초 | 金沸草 |  | 金沸草 | 4469 |
| 207 | Isatidis Folium | 대청엽 | 大青叶 蓼大青叶 |  | 大青叶, 蓼大青叶 | 3416, 7869 |
| 208 | Isatidis Radix | 판람근 | 板蓝根 |  | 板蓝根 | 3370 |
| 209 | Juglandis Semen | 호도 | 核桃仁 |  | 核桃仁 | 1164, 7482 |
| 210 | Junci Medulla | 등심초 | 灯心草 | トウシンソウ | 灯心草 | 4163 |
| 211 | Kaempferiae Rhizoma | 산내 | 山柰 |  | 山柰 | 5505 |
| 212 | Kaki Calyx | 시체 | 柿蒂 | シテイ | 柿蒂 | 2910 |
| 213 | Kalopanacis Cortex | 해동피 | 海桐皮 |  | 海桐皮 | 3519 |
| 214 | Kochiae Fructus | 지부자 | 地肤子 |  | 地肤子 | 1712 |
| 215 | Laminariae Japonicae Thallus | 곤포 | 昆布 |  | 昆布 | 2156 |
| 216 | Lasiosphaera seu Calvatia | 마발 | 马勃 |  | 马勃 | 4551 |
| 217 | Leonuri Herba | 익모초 | 益母草 | ヤクモソウ | 益母草 | 7382 |
| 218 | Leonuri Semen | 충위자 | 茺蔚子 |  | 茺蔚子 | 1844 |
| 219 | Lepidii seu Descurainiae Semen | 정력자 | 葶苈子 |  | 葶苈子 | 4221 |
| 220 | Ligustici Tenuissimi Rhizoma et Radix | 고본 | 蒿本 | コウホン | 蒿本 | 3147, 8081 |
| 221 | Ligustri Fructus | 여정실 | 女贞子 |  | 女贞子 | 6651 |
| 222 | Lilii Bulbus | 백합 | 百合 | ビャクゴウ | 百合 | 2215 |
| 223 | Linderae Radix | 오약 | 乌药 | ウヤク | 乌药 | 6681 |
| 224 | Lini Semen | 아마인 | 亚麻子 |  | 亚麻子 | 6529 |
| 225 | Liquidambaris Fructus | 노로통 | 路路通 |  | 路路通 | 1692 |
| 226 | Liquidambaris Storax | 소합향 | 苏合香 |  | 苏合香 | 5422 |
| 227 | Litchi Semen | 여지핵 | 荔枝核 |  | 荔枝核 | 4506 |
| 228 | Lithospermi Radix | 자근 | 紫草 | シコン | 紫草 | 3969 |
| 229 | Lobeliae Chinensis Herba | 반변련 | 半边莲 |  | 半边莲 | 7608 |
| 230 | Lonicerae Japonicae Flos | 금은화 | 金银花 | キンギンカ | 金银花 | 2475 |
| 231 | Loranthi Ramulus et Folium | 상기생 | 桑寄生 |  | 桑寄生 | 6690 |
| 232 | Lycii Fructus | 구기자 | 枸杞子 | クコシ | 枸杞子 | 7307 |
| 233 | Lycii Radicis Cortex | 지골피 | 地骨皮 | ジコッピ | 地骨皮 | 5106 |
| 234 | Lycopi Herba | 택란 | 泽兰 |  | 泽兰 | 1123 |
| 235 | Lycopodii Herba | 신근초 | 伸筋草 |  | 伸筋草 | 2920 |
| 236 | Lysimachiae Herba | 금전초 | 金钱草 |  | 金钱草 | 7238 |
| 237 | Magnoliae Cortex | 후박 | 厚朴 | コウボク | 厚朴 | 3867, 5050 |
| 238 | Magnoliae Flos | 신이 | 辛夷 | シンイ | 辛夷 | 1602 |
| 239 | Mahoniae Caulis | 공로목 | 功劳木 |  | 功劳木 | 2540 |
| 240 | Malvae Fructus | 동규자 | 冬葵果 |  | 冬葵果 | 1412, 4788 |
| 241 | Maydis Stigma | 옥촉서예 | 玉米须 |  | 玉米须 | 3197, 7789 |
| 242 | Melandrii Herba | 왕불류행 | 王不留行 |  | 王不留行 | 1472 |
| 243 | Meliae Cortex | 고련피 | 苦楝皮 |  | 苦楝皮 | 3364 |
| 244 | Meliae Fructus | 천련자 | 川楝子 | センレンシ | 川楝子 | 2502 |
| 245 | Melo Semen | 첨과자 | 甜瓜子 |  | 甜瓜子 | 7586 |
| 246 | Menispermi Rhizoma | 북두근 | 北豆根 |  | 北豆根 | 3675 |
| 247 | Menthae Herba | 박하 | 薄荷 | ハッカ | 薄荷 | 5534 |
| 248 | Mori Folium | 상엽 | 桑叶 |  | 桑叶 | 2356 |
| 249 | Mori Fructus | 상심자 | 桑椹 |  | 桑椹 | 7298 |
| 250 | Mori Radicis Cortex | 상백피 | 桑白皮 | ソウハクヒ | 桑白皮 | 5292, 7253 |
| 251 | Mori Ramulus | 상지 | 桑枝 |  | 桑枝 | 5020 |
| 252 | Morindae Radix | 파극천 | 巴戟天 |  | 巴戟天 | 6802 |
| 253 | Moutan Radicis Cortex | 목단피 | 牡丹皮 | ボタンピ | 牡丹皮 | 5813 |
| 254 | Mume Flos | 매화 | 梅花 |  | 梅花 | 5702 |
| 255 | Mume Fructus | 오매 | 乌梅 | ウバイ | 乌梅 | 5279 |
| 256 | Myristicae Semen | 육두구 | 肉豆蔻 | ニクズク | 肉豆蔻 | 6668 |
| 257 | Myrrha | 몰약 | 没药 |  | 没药 | 4741 |
| 258 | Nardostachyos Radix et Rhizoma | 감송향 | 甘松 |  | 甘松 | 7662 |
| 259 | Nelumbinis Folium | 하엽 | 荷叶 |  | 荷叶 | 4171 |
| 260 | Nelumbinis Plumula | 연자심 | 莲子心 |  | 莲子心 | 5124 |
| 261 | Nelumbinis Receptaculum | 연방 | 莲房 |  | 莲房 | 8086 |
| 262 | Nelumbinis Rhizomatis Nodus | 우절 | 藕节 |  | 藕节 | 2185, 4264 |
| 263 | Nelumbinis Stamen | 연수 | 莲须 |  | 莲须 | 6344 |
| 264 | Notoginseng Radix et Rhizoma | 삼칠 | 三七 | サンシチニンジン | 三七 | 2443 |
| 265 | Oldenlandiae Diffusae Herba | 백화사설초 | 白花蛇舌草 |  | 白花蛇舌草 | 6341 |
| 266 | Olibanum | 유향 | 乳香 |  | 乳香 | 2834 |
| 267 | Omphalia | 뇌환 | 雷丸 |  | 雷丸 | 5308 |
| 268 | Oroxyli Semen | 목호접 | 木蝴蝶 |  | 木蝴蝶 | 1780 |
| 269 | Oryzae Rhizoma et Radix | 나도근 |  |  | 糯稻根 | 6667 |
| 270 | Osterici seu Notopterygii Radix et Rhizoma | 강활 | 羌活 | キョウカツ | 羌活 | 4776 |
| 271 | Paeoniae Radix | 작약 | 白芍 赤芍 | シャクヤク | 白芍, 赤芍 | 1957, 3997, 6926 |
| 272 | Panacis Quinquefolii Radix | 서양삼 | 西洋参 |  | 西洋参 | 2564 |
| 273 | Papaveris Pericarpium | 앵속각 | 罂粟壳 |  | 罂粟壳 | 6842 |
| 274 | Patriniae Radix | 패장 | 败酱草 |  | 败酱草 | 3973, 5328 |
| 275 | Perillae Folium | 자소엽 | 紫苏叶 | ソヨウ | 紫苏 | 4762, 7128 |
| 276 | Perillae Fructus | 자소자 | 紫苏子 | シソシ | 紫苏子 | 1464, 3604 |
| 277 | Persicae Semen | 도인 | 桃仁 | トウニン | 桃仁 | 6079 |
| 278 | Peucedani Radix | 전호 | 前胡 紫花前胡 | ゼンコ | 前胡, 紫花前胡 | 3172, 6338 |
| 279 | Pharbitidis Semen | 견우자 | 牵牛子 | ケンゴシ | 牵牛子 | 4848 |
| 280 | Phellodendri Cortex | 황백 | 黄柏 关黄柏 | オウバク | 黄柏, 关黄柏 | 8114 |
| 281 | Phragmitis Rhizoma | 노근 | 芦根 |  | 芦根 | 5927 |
| 282 | Phyllanthi Fructus | 여감자 | 余甘子 |  | 余甘子 | 5953 |
| 283 | Physochlainae Radix | 화산삼 | 华山参 |  | 华山参 | 3013 |
| 284 | Phytolaccae Radix | 상륙 | 商陆 |  | 商陆 | 3414 |
| 285 | Picrorhizae Rhizoma | 호황련 | 胡黄连 |  | 胡黄连 | 3706 |
| 286 | Pinelliae Tuber | 반하 | 半夏 | ハンゲ | 半夏 | 5800 |
| 287 | Pini Pollen | 송화분 | 松花粉 |  | 松花粉 | 4492 |
| 288 | Piperis Kadsurae Caulis | 해풍등 | 海风藤 |  | 海风藤 | 7221 |
| 289 | Piperis Longi Fructus | 필발 | 荜茇 |  | 荜茇 | 3043 |
| 290 | Piperis Nigri Fructus | 후추 | 胡椒 |  | 胡椒 | 1970 |
| 291 | Plantaginis Herba | 차전초 | 车前草 | シャゼンソウ | 车前草 | 6593 |
| 292 | Plantaginis Semen | 차전자 | 车前子 | シャゼンシ | 车前子 | 3760 |
| 293 | Platycodonis Radix | 길경 | 桔梗 | キキョウ | 桔梗 | 3507 |
| 294 | Pogostemonis Herba | 광곽향 | 广藿香 | カッコウ | 广藿香 | 7983 |
| 295 | Polygonati Odorati Rhizoma | 옥죽 | 玉竹 |  | 玉竹 | 8088 |
| 296 | Polygonati Rhizoma | 황정 | 黄精 | オウセイ | 黄精 | 6854 |
| 297 | Polygoni Avicularis Herba | 편축 | 萹蓄 |  | 萹蓄 | 7426 |
| 298 | Polygoni Cuspidati Rhizoma et Radix | 호장근 | 虎杖 |  | 虎杖 | 4306 |
| 299 | Polygoni Orientalis Fructus | 수홍화자 | 水红花子 |  | 水红花子 | 1532 |
| 300 | Polyporus | 저령 | 猪苓 | チョレイ | 猪苓 | 4883 |
| 301 | Ponciri Fructus Immaturus | 지실 | 枳实 | キジツ | 枳实 | 3379 |
| 302 | Poria Sclerotium | 복령 | 茯苓 | ブクリョウ | 茯苓 | 7870 |
| 303 | Portulacae Herba | 마치현 | 马齿苋 |  | 马齿苋 | 1434 |
| 304 | Potentillae Discoloris Herba | 번백초 | 翻白草 |  | 翻白草 | 7343 |
| 305 | Potentillae Herba | 위릉채 | 委陵菜 |  | 委陵菜 | 4389 |
| 306 | Prunellae Spica | 하고초 | 夏枯草 | カゴソウ | 夏枯草 | 8202 |
| 307 | Pruni Japonicae Semen | 욱리인 | 郁李仁 |  | 郁李仁 | 7422 |
| 308 | Pseudostellariae Radix | 태자삼 | 太子参 |  | 太子参 | 4211 |
| 309 | Puerariae Flos | 갈화 |  |  | 葛花 | 5889 |
| 310 | Puerariae Radix | 갈근 | 葛根 | カッコン | 葛根 | 6205 |
| 311 | Pulsatillae Radix | 백두옹 | 白头翁 |  | 白头翁 | 6999 |
| 312 | Pyrolae Herba | 녹제초 | 鹿衔草 |  | 鹿衔草 | 1393 |
| 313 | Quisqualis Fructus | 사군자 | 使君子 |  | 使君子 | 2202 |
| 314 | Ranunculi Ternati Radix | 묘조초 | 猫爪草 |  | 猫爪草 | 2422 |
| 315 | Raphani Semen | 내복자 | 莱菔子 |  | 莱菔子 | 7954 |
| 316 | Rehmanniae Radix Preparata | 숙지황 | 熟地黄 |  | 熟地黄 | 5894 |
| 317 | Rhapontici Radix | 누로 | 漏芦 |  | 漏芦 | 8021 |
| 318 | Rhei Radix et Rhizoma | 대황 | 大黄 | ダイオウ | 大黄 | 7047 |
| 319 | Rhododendri Daurici Folium | 만산홍 | 满山红 |  | 满山红 | 4547 |
| 320 | Rhododendri Mollis Flos | 요양화 | 闹羊花 |  | 闹羊花 | 5827 |
| 321 | Ricini Semen | 피마자 | 蓖麻子 |  | 蓖麻子 | 5699 |
| 322 | Rosae Chinensis Flos | 월계화 | 月季花 |  | 月季花 | 1370 |
| 323 | Rosae Laevigatae Fructus | 금앵자 | 金樱子 |  | 金樱子 | 1630 |
| 324 | Rosae Rugosae Flos | 매괴화 | 玫瑰花 |  | 玫瑰花 | 2716 |
| 325 | Rubi Fructus | 복분자 | 覆盆子 |  | 覆盆子 | 5009 |
| 326 | Rubiae Radix | 천초근 | 茜草 |  | 茜草 | 3696 |
| 327 | Salviae Miltiorrhizae Radix | 단삼 | 丹参 | タンジン | 丹参 | 7296 |
| 328 | Santali Albi Lignum | 백단향 | 檀香 |  | 檀香 | 3390 |
| 329 | Saposhnikoviae Radix | 방풍 | 防风 | ボウフウ | 防风 | 7847 |
| 330 | Sarcandrae Herba | 종절풍 | 肿节风 |  | 肿节风 | 6418 |
| 331 | Sargassum | 해조 | 海藻 |  | 海藻 | 3646 |
| 332 | Sargentodoxae Caulis | 대혈등 | 大血藤 |  | 大血藤 | 7141 |
| 333 | Saururi Herba | 삼백초 | 三白草 |  | 三白草 | 3085 |
| 334 | Saxifragae Herba | 호이초 |  |  | 虎耳草 | 5727 |
| 335 | Scapharcae seu Tegillarcae Concha | 와릉자 | 瓦楞子 |  | 瓦楞子 | 2915 |
| 336 | Schisandrae Fructus | 오미자 | 五味子 | ゴミシ | 北五味子 | 1518 |
| 337 | Schizonepetae Spica | 형개 | 荆芥 荆芥穗 | ケイガイ | 荆芥, 荆芥穗 | 1287, 4526 |
| 338 | Scrophulariae Radix | 현삼 | 玄参 | ゲンジン | 玄参 | 5034 |
| 339 | Scutellariae Barbatae Herba | 반지련 | 半枝莲 |  | 半枝莲 | 5541 |
| 340 | Scutellariae Radix | 황금 | 黄芩 | オウゴン | 黄芩 | 6700 |
| 341 | Sedi Herba | 수분초 | 垂盆草 |  | 垂盆草 | 1923 |
| 342 | Selaginellae Herba | 권백 | 卷柏 |  | 卷柏 | 1346 |
| 343 | Sennae Folium | 번사엽 | 番泻叶 | センナ | 番泻叶 | 7254 |
| 344 | Sesami Semen Nigra | 흑지마 | 黑芝麻 | ゴマ | 黑芝麻 | 6841 |
| 345 | Silybi Fructus | 수비계 | 水飞蓟 |  | 水飞蓟 | 5134 |
| 346 | Sinomeni Caulis et Rhizoma | 방기 | 防己 青风藤 | ボウイ | 防己, 青风藤 | 4696 |
| 347 | Siphonostegiae Herba | 북유기노 | 北刘寄奴 |  | 北刘寄奴 | 1889 |
| 348 | Siraitiae Fructus | 나한과 | 罗汉果 |  | 罗汉果 | 3029 |
| 349 | Smilacis Rhizoma | 토복령 | 土茯苓 菝葜 | サンキライ | 土茯苓, 菝葜 | 1626, 2756 |
| 350 | Solani Nigri Herba | 용규 | 龙葵 |  | 龙葵 | 5645 |
| 351 | Sophorae Flos | 괴화 | 槐花 | カイカ | 槐花 | 5512 |
| 352 | Sophorae Fructus | 괴각 | 槐角 |  | 槐角 | 4893 |
| 353 | Sophorae Radix | 고삼 | 苦参 | クジン | 苦参 | 7741 |
| 354 | Sophorae Tonkinensis Radix et Rhizoma | 산두근 | 山豆根 | サンズコン | 山豆根 | 1419 |
| 355 | Sparganii Rhizoma | 삼릉 | 三棱 |  | 三棱 | 7888 |
| 356 | Spatholobi Caulis | 계혈등 | 鸡血藤 |  | 鸡血藤 | 6136 |
| 357 | Spirodelae Herba | 부평 | 浮萍 |  | 浮萍 | 3768 |
| 358 | Stellariae seu Gypsophilae Radix | 은시호 | 银柴胡 |  | 银柴胡 | 2696 |
| 359 | Stemonae Radix | 백부근 | 百部 |  | 百部 | 3454 |
| 360 | Sterculiae Lychnophorae Semen | 반대해 | 胖大海 |  | 胖大海 | 7502 |
| 361 | Strychni Semen | 마전자 | 马钱子 | ホミカ | 马钱子 | 1421 |
| 362 | Swertiae Mileensis Herba | 청엽담 | 青叶胆 |  | 青叶胆 | 4472 |
| 363 | Syzygii Flos | 정향 | 丁香 | チョウジ | 丁香 | 5374 |
| 364 | Tamarics Cacumen | 정류 | 西河柳 |  | 西河柳 | 5172 |
| 365 | Terminaliae Fructus | 가자 | 诃子 | カシ | 诃子 | 7932 |
| 366 | Thujae Orientalis Folium | 측백엽 | 侧柏叶 |  | 侧柏叶 | 4272 |
| 367 | Tinosporae Radix | 금과람 | 金果榄 |  | 金果揽 | 4665 |
| 368 | Trachelospermi Caulis | 낙석등 | 络石藤 |  | 络石藤 | 4021 |
| 369 | Tribuli Fructus | 질려자 | 蒺藜 | シツリシ | 蒺藜 | 1498 |
| 370 | Trichosanthis Fructus | 과루 | 瓜蒌 |  | 瓜蒌 | 2612 |
| 371 | Trichosanthis Radix | 괄루근 | 天花粉 | カロコン | 天花粉 | 1501 |
| 372 | Trigonellae Semen | 호로파 | 胡芦巴 |  | 胡芦巴 | 1098 |
| 373 | Typhae Pollen | 포황 | 蒲黄 |  | 蒲黄 | 3303 |
| 374 | Uncariae Ramulus cum Uncus | 조구등 | 钩藤 | チョウトウコウ | 钩藤 | 7305 |
| 375 | Verbenae Herba | 마편초 | 马鞭草 |  | 马鞭草 | 7962 |
| 376 | Vignae Angularis Semen | 적소두 | 赤小豆 |  | 赤小豆 | 5082 |
| 377 | Violae Herba | 자화지정 | 紫花地丁 |  | 紫花地丁 | 6597 |
| 378 | Visci Ramulus et Folium | 곡기생 | 槲寄生 |  | 槲寄生 | 4132 |
| 379 | Viticis Fructus | 만형자 | 蔓荆子 | マンケイシ | 蔓荆子 | 7428 |
| 380 | Viticis Negundo Folium | 모형엽 | 牡荆叶 |  | 牡荆叶 | 5403 |
| 381 | Xanthii Fructus | 창이자 | 苍耳子 |  | 苍耳子 | 6163 |
| 382 | Zanthoxyli Pericarpium | 산초 | 花椒 | サンショウ ショクショウ | 花椒 | 4083 |
| 383 | Zanthoxyli Radix | 양면침 | 两面针 |  | 两面针 | 7091 |
| 384 | Zingiberis Rhizoma | 건강 | 干姜 | カンキョウ | 干姜 | 1656 |
| 385 | Zingiberis Rhizoma Recens | 생강 | 生姜 | ショウキョウ | 生姜 | 7484 |
| 386 | Zizyphi Fructus | 대조 | 大枣 | タイソウ | 大枣 | 1076 |
| 387 | Zizyphi Semen | 산조인 | 酸枣仁 | サンソウニン | 酸枣仁 | 4930, 6272 |

**Table S3 List of licorice compounds in TM-MC and their existence in TCMSP and TCMID.**

| No | ID | INCHIKEY | Only TM-MC | TM-MC, TCMSP, and TCMID | TM-MC and TCMSP | TM-MC and TCMID |
| --- | --- | --- | --- | --- | --- | --- |
| 1 | 109 | KSEBMYQBYZTDHS-HWKANZROSA-N |  |  |  | O |
| 2 | 111 | NGSWKAQJJWESNS-ZZXKWVIFSA-N | O |  |  |  |
| 3 | 124 | KZNIFHPLKGYRTM-UHFFFAOYSA-N | O |  |  |  |
| 4 | 132 | REFJWTPEDVJJIY-UHFFFAOYSA-N |  |  | O |  |
| 5 | 135 | IKGXIBQEEMLURG-NVPNHPEKSA-N |  | O |  |  |
| 6 | 136 | IYRMWMYZSQPJKC-UHFFFAOYSA-N |  |  | O |  |
| 7 | 142 | URFCJEUYXNAHFI-ZDUSSCGKSA-N |  |  | O |  |
| 8 | 154 | FTVWIRXFELQLPI-ZDUSSCGKSA-N |  |  | O |  |
| 9 | 157 | TZBJGXHYKVUXJN-UHFFFAOYSA-N | O |  |  |  |
| 10 | 172 | FJKROLUGYXJWQN-UHFFFAOYSA-N | O |  |  |  |
| 11 | 176 | CKLJMWTZIZZHCS-REOHCLBHSA-N | O |  |  |  |
| 12 | 177 | WHUUTDBJXJRKMK-VKHMYHEASA-N | O |  |  |  |
| 13 | 178 | MTCFGRXMJLQNBG-REOHCLBHSA-N | O |  |  |  |
| 14 | 180 | HNDVDQJCIGZPNO-YFKPBYRVSA-N | O |  |  |  |
| 15 | 183 | ODKSFYDXXFIFQN-BYPYZUCNSA-N | O |  |  |  |
| 16 | 189 | COLNVLDHVKWLRT-QMMMGPOBSA-N | O |  |  |  |
| 17 | 191 | ROHFNLRQFUQHCH-YFKPBYRVSA-N | O |  |  |  |
| 18 | 193 | ONIBWKKTOPOVIA-BYPYZUCNSA-N | O |  |  |  |
| 19 | 232 | OIRDTQYFTABQOQ-KQYNXXCUSA-N | O |  |  |  |
| 20 | 264 | FERIUCNNQQJTOY-UHFFFAOYSA-N | O |  |  |  |
| 21 | 265 | PCMORTLOPMLEFB-ONEGZZNKSA-N |  |  |  | O |
| 22 | 290 | WLJVXDMOQOGPHL-UHFFFAOYSA-N | O |  |  |  |
| 23 | 309 | NYHBQMYGNKIUIF-UUOKFMHZSA-N | O |  |  |  |
| 24 | 311 | IQFYYKKMVGJFEH-XLPZGREQSA-N | O |  |  |  |
| 25 | 314 | DRTQHJPVMGBUCF-XVFCMESISA-N | O |  |  |  |
| 26 | 315 | CKTSBUTUHBMZGZ-SHYZEUOFSA-N | O |  |  |  |
| 27 | 316 | UHDGCWIWMRVCDJ-XVFCMESISA-N | O |  |  |  |
| 28 | 320 | UGQMRVRMYYASKQ-KQYNXXCUSA-N | O |  |  |  |
| 29 | 323 | ZYGHJZDHTFUPRJ-UHFFFAOYSA-N | O |  |  |  |
| 30 | 375 | KZJWDPNRJALLNS-VJSFXXLFSA-N |  | O |  |  |
| 31 | 454 | NUJGJRNETVAIRJ-UHFFFAOYSA-N | O |  |  |  |
| 32 | 650 | QSJXEFYPDANLFS-UHFFFAOYSA-N | O |  |  |  |
| 33 | 931 | UFWIBTONFRDIAS-UHFFFAOYSA-N | O |  |  |  |
| 34 | 998 | DTUQWGWMVIHBKE-UHFFFAOYSA-N | O |  |  |  |
| 35 | 1183 | MWOOGOJBHIARFG-UHFFFAOYSA-N | O |  |  |  |
| 36 | 1466 | BSAIUMLZVGUGKX-BQYQJAHWSA-N | O |  |  |  |
| 37 | 1514 | SLEWMHCSJWMRAU-VQTJNVASSA-N | O |  |  |  |
| 38 | 1545 | DFPMSGMNTNDNHN-ZPHOTFPESA-N |  |  | O |  |
| 39 | 1880 | COCYGNDCWFKTMF-UHFFFAOYSA-N | O |  |  |  |
| 40 | 2097 | SLKHLLNCFGPWAZ-UHFFFAOYSA-N |  |  | O |  |
| 41 | 2098 | OCTMMRDISKQYEL-UHFFFAOYSA-N | O |  |  |  |
| 42 | 2099 | WBIAZFIKQRCKAK-XAXWTWDHSA-N | O |  |  |  |
| 43 | 2100 | HWEXTYSAVVMOIR-XAXWTWDHSA-N | O |  |  |  |
| 44 | 2270 | QGPHHWRNFMTVGA-VMPITWQZSA-N | O |  |  |  |
| 45 | 2602 | JZFSMVXQUWRSIW-BTJIZOSBSA-N |  |  | O |  |
| 46 | 2758 | WEEGYLXZBRQIMU-UHFFFAOYSA-N | O |  |  |  |
| 47 | 2940 | LPLVUJXQOOQHMX-IOHDZAKGSA-N |  | O |  |  |
| 48 | 2944 | RMIVRCBSQPCSCQ-BSMXFTQLSA-N |  |  |  | O |
| 49 | 2945 | RMIVRCBSQPCSCQ-BDANYOJNSA-N |  |  |  | O |
| 50 | 3036 | GOQYKNQRPGWPLP-UHFFFAOYSA-N | O |  |  |  |
| 51 | 3230 | MPDGHEJMBKOTSU-PMTKVOBESA-N |  | O |  |  |
| 52 | 3340 | BYSMNFVBLGSCSW-XBXARRHUSA-N | O |  |  |  |
| 53 | 3632 | ZUJZTVOVDKEJKY-KCHHQKCCSA-N | O |  |  |  |
| 54 | 3633 | OBFDSOYVKVRFGY-GKDIEYKBSA-N | O |  |  |  |
| 55 | 3641 | FEJIMIALLVHITA-LTRMMDMMSA-N | O |  |  |  |
| 56 | 3699 | YDBRGXOKOCKUMR-TUTITMQTSA-N | O |  |  |  |
| 57 | 3701 | TZCOLOGTIORRHN-BUVILJDDSA-N | O |  |  |  |
| 58 | 3702 | BATKFWMEEIAGGW-LRXQWTQBSA-N | O |  |  |  |
| 59 | 3713 | GSEPOEIKWTXTHS-WTMNEXLISA-N |  |  | O |  |
| 60 | 3764 | LNIQZRIHAMVRJA-UHFFFAOYSA-N |  |  | O |  |
| 61 | 4007 | NTOPKICPEQUPPH-UHFFFAOYSA-N | O |  |  |  |
| 62 | 4385 | AFCJRSVBONSNTR-UHFFFAOYSA-N | O |  |  |  |
| 63 | 4398 | WDZPZEAQKBBZSM-FPYGCLRLSA-N | O |  |  |  |
| 64 | 4547 | CDOSHBSSFJOMGT-UHFFFAOYSA-N | O |  |  |  |
| 65 | 4551 | ZZAJQOPSWWVMBI-UHFFFAOYSA-N |  |  | O |  |
| 66 | 4730 | YJRGJZBUUOQBFV-UHFFFAOYSA-N | O |  |  |  |
| 67 | 4736 | PQYIMRBOJKGQGX-UTSBKAFOSA-N | O |  |  |  |
| 68 | 4762 | GQHOUVCWXQQNKY-UHFFFAOYSA-N | O |  |  |  |
| 69 | 4768 | YHZDBBUEVZEOIY-BBRMVZONSA-N | O |  |  |  |
| 70 | 4841 | OVSQVDMCBVZWGM-QSOFNFLRSA-N |  |  | O |  |
| 71 | 4895 | IXEZXGJHEPIUPS-GSMWXASISA-N | O |  |  |  |
| 72 | 4979 | SBQBKTSYEKPBJF-UHFFFAOYSA-N |  | O |  |  |
| 73 | 4980 | ACDSUMGMZHXCRO-UHFFFAOYSA-N | O |  |  |  |
| 74 | 5109 | HLDYLAJAWSKPFZ-QDPIGISRSA-N | O |  |  |  |
| 75 | 5690 | FLLVBTCXQPHZJB-UHFFFAOYSA-N | O |  |  |  |
| 76 | 5912 | SCZVLDHREVKTSH-UHFFFAOYSA-N | O |  |  |  |
| 77 | 6054 | WRMNZCZEMHIOCP-UHFFFAOYSA-N | O |  |  |  |
| 78 | 6184 | JARKCYVAAOWBJS-UHFFFAOYSA-N | O |  |  |  |
| 79 | 6299 | UCHYSPNEUSDFQR-UHFFFAOYSA-N |  |  | O |  |
| 80 | 6361 | KBEFFXBSHFUQLT-KRWDZBQOSA-N |  |  | O |  |
| 81 | 6401 | UCJQQMAJZXXGTH-ULSDOHGRSA-N | O |  |  |  |
| 82 | 6508 | AAWZDTNXLSGCEK-LNVDRNJUSA-N | O |  |  |  |
| 83 | 6638 | BMADVHDZKAZTNF-UHFFFAOYSA-N | O |  |  |  |
| 84 | 6646 | LCAWNFIFMLXZPQ-UHFFFAOYSA-N |  |  | O |  |
| 85 | 6714 | JZOYQACMFLYEJG-UHFFFAOYSA-N | O |  |  |  |
| 86 | 6855 | SGEWCQFRYRRZDC-VPRICQMDSA-N |  |  | O |  |
| 87 | 6874 | XJUDJFVOICLOMY-SFHVURJKSA-N |  |  | O |  |
| 88 | 6875 | GRZCOESTZFGKMN-UHFFFAOYSA-N | O |  |  |  |
| 89 | 6876 | KIQQFVJHWNCGAU-UHFFFAOYSA-N | O |  |  |  |
| 90 | 6989 | MGSRCZKZVOBKFT-UHFFFAOYSA-N | O |  |  |  |
| 91 | 7284 | BYGQBDHUGHBGMD-UHFFFAOYSA-N | O |  |  |  |
| 92 | 7710 | OALYTRUKMRCXNH-UHFFFAOYSA-N | O |  |  |  |
| 93 | 8079 | HGCIXCUEYOPUTN-UHFFFAOYSA-N | O |  |  |  |
| 94 | 8175 | KSMVZQYAVGTKIV-UHFFFAOYSA-N | O |  |  |  |
| 95 | 8314 | WLAMNBDJUVNPJU-UHFFFAOYSA-N | O |  |  |  |
| 96 | 8433 | NZZIMKJIVMHWJC-UHFFFAOYSA-N |  |  | O |  |
| 97 | 8641 | GAARAMNTPQAIOT-JSYAWONVSA-N | O |  |  |  |
| 98 | 8697 | KSDSYIXRWHRPMN-JAJIQNMNSA-N |  |  | O |  |
| 99 | 8815 | ZFMSMUAANRJZFM-UHFFFAOYSA-N | O |  |  |  |
| 100 | 8892 | FUZZWVXGSFPDMH-UHFFFAOYSA-N | O |  |  |  |
| 101 | 8907 | XUGNVMKQXJXZCD-UHFFFAOYSA-N | O |  |  |  |
| 102 | 9395 | YWINIKYRUNUZAA-UHFFFAOYSA-N | O |  |  |  |
| 103 | 9449 | ZQSIJRDFPHDXIC-UHFFFAOYSA-N | O |  |  |  |
| 104 | 9626 | UACNRZUVCUEUPY-UHFFFAOYSA-N | O |  |  |  |
| 105 | 10114 | MPDGHEJMBKOTSU-YKLVYJNSSA-N |  | O |  |  |
| 106 | 10394 | NMHMNPHRMNGLLB-UHFFFAOYSA-N | O |  |  |  |
| 107 | 10430 | GWYFCOCPABKNJV-UHFFFAOYSA-N | O |  |  |  |
| 108 | 10494 | MIJYXULNPSFWEK-GTOFXWBISA-N |  |  | O |  |
| 109 | 10624 | HKQYGTCOTHHOMP-UHFFFAOYSA-N |  | O |  |  |
| 110 | 11213 | AOVQEAUIBICOLQ-UHFFFAOYSA-N | O |  |  |  |
| 111 | 11214 | SFIMWOHAIWWSQJ-UHFFFAOYSA-N | O |  |  |  |
| 112 | 11215 | COLMVFWKLOZOOP-UHFFFAOYSA-N |  |  | O |  |
| 113 | 11216 | SJIZTMNAKZAOTA-UHFFFAOYSA-N |  |  | O |  |
| 114 | 11237 | UCUBMAVPVJYHIR-OVGRPOLTSA-N |  |  |  | O |
| 115 | 11253 | FIYVYQFNWRYZHD-UHFFFAOYSA-N | O |  |  |  |
| 116 | 11254 | GNCVLCAWOLDBIZ-UHFFFAOYSA-N | O |  |  |  |
| 117 | 11279 | NZYSZZDSYIBYLC-UHFFFAOYSA-N |  | O |  |  |
| 118 | 11300 | JWYWPLOBJMBPAH-UHFFFAOYSA-N | O |  |  |  |
| 119 | 11305 | JOQWUUJQWPZLAT-UHFFFAOYSA-N |  |  |  | O |
| 120 | 11318 | VXOZFACLCCVUMV-UHFFFAOYSA-N | O |  |  |  |
| 121 | 11420 | HMSWAIKSFDFLKN-UHFFFAOYSA-N | O |  |  |  |
| 122 | 11552 | YGHRJJRRZDOVPD-UHFFFAOYSA-N | O |  |  |  |
| 123 | 11660 | YUSWMAULDXZHPY-UHFFFAOYSA-N | O |  |  |  |
| 124 | 11719 | RNBLSJGPSGNSIN-UHFFFAOYSA-N |  |  | O |  |
| 125 | 11876 | YNWXJFQOCHMPCK-LXGDFETPSA-N |  | O |  |  |
| 126 | 12086 | CJUFYKORDZSOLF-INIZCTEOSA-N |  | O |  |  |
| 127 | 12254 | JPUKWEQWGBDDQB-QSOFNFLRSA-N |  | O |  |  |
| 128 | 12282 | RTATXGUCZHCSNG-KYGWAIEOSA-N |  | O |  |  |
| 129 | 12391 | YCOZIPAWZNQLMR-UHFFFAOYSA-N | O |  |  |  |
| 130 | 12405 | HOWGUJZVBDQJKV-UHFFFAOYSA-N | O |  |  |  |
| 131 | 12574 | KRWXHCWICDLYOY-UHFFFAOYSA-N |  | O |  |  |
| 132 | 12577 | KAZSKMJFUPEHHW-DHZHZOJOSA-N |  | O |  |  |
| 133 | 12578 | DRDRYGIIYOPBBZ-XBXARRHUSA-N |  |  | O |  |
| 134 | 12579 | HJGURBGBPIKRER-UHFFFAOYSA-N |  | O |  |  |
| 135 | 12580 | JNDPLDZUOFZXIG-UHFFFAOYSA-N |  | O |  |  |
| 136 | 12584 | BCNKILSUUHWRTG-QVOQZYHNSA-N |  | O |  |  |
| 137 | 12588 | PLPHOQSHVPRIJE-WOXPWJABSA-N |  |  |  | O |
| 138 | 12589 | NUXMXYUOCFBORQ-BHOOJSBVSA-N |  | O |  |  |
| 139 | 12590 | SLWCVFLNZDOMEZ-QOXOVGTHSA-N |  | O |  |  |
| 140 | 12595 | IPBGQSWRSQPGCF-NMLFLIHHSA-N |  | O |  |  |
| 141 | 12597 | KTGMWPWFLCUDMK-FROTZJPNSA-N | O |  |  |  |
| 142 | 12601 | GBRZTUJCDFSIHM-QGZVFWFLSA-N |  | O |  |  |
| 143 | 12604 | NIZFPXZQERMCLE-KVFWHIKKSA-N |  |  | O |  |
| 144 | 12648 | FTVKHUHJWDMWIR-DWMQJYMWSA-N |  |  | O |  |
| 145 | 12655 | FURUXTVZLHCCNA-AWEZNQCLSA-N |  | O |  |  |
| 146 | 12676 | DEMKZLAVQYISIA-ZRWXNEIDSA-N |  | O |  |  |
| 147 | 12756 | JBFHTYHTHYHCDJ-UHFFFAOYSA-N | O |  |  |  |
| 148 | 13558 | KSDSYIXRWHRPMN-RGHIGTIISA-N |  |  | O |  |
| 149 | 13559 | MFQIWHVVFBCURA-LKBAIHPRSA-N | O |  |  |  |
| 150 | 13633 | URAXFHQPJFAYDZ-UHFFFAOYSA-N | O |  |  |  |
| 151 | 14982 | LPLVUJXQOOQHMX-QWBHMCJMSA-N |  | O |  |  |
| 152 | 14983 | LPLVUJXQOOQHMX-IMQZSDMHSA-N |  | O |  |  |
| 153 | 15002 | LPLVUJXQOOQHMX-GDVJMDODSA-N |  | O |  |  |
| 154 | 15003 | UESGIOZUTMALSQ-CPRSUJDVSA-N | O |  |  |  |
| 155 | 15004 | DDTSMRSWKZCDKP-KRSSWZRASA-N | O |  |  |  |
| 156 | 15006 | XRVFNNUXNVWYTI-LLVKDONJSA-N |  |  | O |  |
| 157 | 15007 | BCNKILSUUHWRTG-DROJETJMSA-N |  | O |  |  |
| 158 | 15011 | XKTVQMDIEGLWQD-YYZSXABUSA-N | O |  |  |  |
| 159 | 15012 | IVCITHFPTPZRQC-VNBKKLSBSA-N |  |  | O |  |
| 160 | 15013 | BXFDBOZJOZAQPB-UHFFFAOYSA-N | O |  |  |  |
| 161 | 15014 | YNEXNFNJHSGNFZ-ISFAVVKVSA-N | O |  |  |  |
| 162 | 15015 | RECUKUPTGUEGMW-UHFFFAOYSA-N | O |  |  |  |
| 163 | 15016 | YCYGGFHFSJXFQP-XCIACXCLSA-N | O |  |  |  |
| 164 | 15017 | WBQVRPYEEYUEBQ-CMLHMLJASA-N |  | O |  |  |
| 165 | 15018 | HZLNWXNRNNSNAZ-CUKUBIBCSA-N | O |  |  |  |
| 166 | 15020 | QZQBDAOUQQTONF-MIUWDCFGSA-N | O |  |  |  |
| 167 | 15021 | XHTOWVVXFDSBGH-XBXARRHUSA-N |  |  | O |  |
| 168 | 15022 | XSXCXNLQJOCJTM-UHFFFAOYSA-N | O |  |  |  |
| 169 | 15023 | ILRKKHJEINIICQ-OOFFSTKBSA-N |  |  |  | O |
| 170 | 15026 | WGNIVAMNAWBYRO-UHFFFAOYSA-N |  |  | O |  |
| 171 | 15027 | RMIHCTUWJGVJQB-HNNXBMFYSA-N |  |  | O |  |
| 172 | 15028 | UESGIOZUTMALSQ-FNDKVILYSA-N | O |  |  |  |
| 173 | 15029 | ZLVCXXUAUGUIME-UHFFFAOYSA-N | O |  |  |  |
| 174 | 15030 | HLDYLAJAWSKPFZ-HBKAJLBFSA-N | O |  |  |  |
| 175 | 15031 | MVUIYQOCXQCYMU-NQARLCQHSA-N | O |  |  |  |
| 176 | 15032 | OAXGEVHOTKTLTP-UHFFFAOYSA-N | O |  |  |  |
| 177 | 15034 | OVLUQDOJWGTPHA-UHFFFAOYSA-N |  | O |  |  |
| 178 | 15035 | BLRLIWINXKVPGS-SPTXWYHOSA-N | O |  |  |  |
| 179 | 15037 | BVGZTRWADYNRBE-RWGOFXMDSA-N | O |  |  |  |
| 180 | 15038 | AQSVWWUHJNLLGU-PDWOELFDSA-N | O |  |  |  |
| 181 | 15039 | PVEMGMOWXQUWRD-NJAOXFEXSA-N |  |  | O |  |
| 182 | 15042 | NSRJSISNDPOJOP-CZUORRHYSA-N |  |  | O |  |
| 183 | 15043 | HKUBLIRXXFRGKE-DNNBANOASA-N |  |  | O |  |
| 184 | 15044 | AYHOUUNTAVCXBN-ZDUSSCGKSA-N | O |  |  |  |
| 185 | 15052 | XRVFNNUXNVWYTI-NSHDSACASA-N |  |  | O |  |
| 186 | 15053 | FFDNYMAHNWBKCH-NSHDSACASA-N | O |  |  |  |
| 187 | 15054 | YLYJXNTZVUEFJZ-DODNOZFWSA-N | O |  |  |  |
| 188 | 15055 | WTXMHYXTGODDJX-PUIBNRJISA-N | O |  |  |  |
| 189 | 15056 | AAKHRTZXSZBLFQ-UHFFFAOYSA-N |  |  | O |  |
| 190 | 15057 | AIYQJOLHYJBHDM-UHFFFAOYSA-N | O |  |  |  |
| 191 | 15060 | WCVUIHQUPRXYKT-RGCIIANRSA-N | O |  |  |  |
| 192 | 15062 | ICKWICRCANNIBI-UHFFFAOYSA-N | O |  |  |  |
| 193 | 15088 | DKIYWPRXYDNQFG-XMMPIXPASA-N |  |  | O |  |
| 194 | 15361 | DXDRHHKMWQZJHT-FPYGCLRLSA-N |  | O |  |  |
| 195 | 15362 | IDTLJMKBOGZHMH-WOLDIFTDSA-N | O |  |  |  |
| 196 | 15718 | LWZACZCRAUQSLH-UHFFFAOYSA-N |  |  | O |  |
| 197 | 16772 | BLLBGEGVACQFPS-XGEKIMHTSA-N | O |  |  |  |
| 198 | 16955 | TZNGGQHGAUXOJZ-ADYZCQOVSA-N | O |  |  |  |
| 199 | 16957 | SZHUMUJGHFQABW-LJWJVLCMSA-N | O |  |  |  |
| 200 | 16958 | TZNGGQHGAUXOJZ-PMAXACJDSA-N | O |  |  |  |
| 201 | 16961 | AJNHVDTWPCYIJI-WHWLRNRUSA-N | O |  |  |  |
| 202 | 17169 | MPDGHEJMBKOTSU-IDZWEYSVSA-N |  | O |  |  |
| 203 | 17170 | MPDGHEJMBKOTSU-UPUJGSTDSA-N |  | O |  |  |
| 204 | 18635 | CLUWOWRTHNNBBU-UHFFFAOYSA-N | O |  |  |  |
| 205 | 19309 | INAXVXBDKKUCGI-UHFFFAOYSA-N | O |  |  |  |
| 206 | 19310 | YWHLKYXPLRWGSE-UHFFFAOYSA-N | O |  |  |  |
| 207 | 20002 | VVGOCOMZRGWHPI-ARJAWSKDSA-N | O |  |  |  |
| 208 | 20003 | DQBQWWSFRPLIAX-UHFFFAOYSA-N | O |  |  |  |
| 209 | 20004 | HEQOJEGTZCTHCF-UHFFFAOYSA-N | O |  |  |  |
| 210 | 20007 | HMNKTRSOROOSPP-UHFFFAOYSA-N | O |  |  |  |
| 211 | 20008 | UNYNVICDCJHOPO-UHFFFAOYSA-N | O |  |  |  |
| 212 | 20009 | BGVBGAIWXAXBLP-UHFFFAOYSA-N | O |  |  |  |
| 213 | 20011 | PFQMUQWFRINBBG-UHFFFAOYSA-N |  |  | O |  |
| 214 | 20013 | NUGPQONICGTVNA-UHFFFAOYSA-N | O |  |  |  |
| 215 | 20016 | RIDRQWKYWXHAOD-UHFFFAOYSA-N | O |  |  |  |
| 216 | 20254 | JPMYFOBNRRGFNO-UHFFFAOYSA-N | O |  |  |  |
| 217 | 21298 | JYRFVDHHGPHQBG-UHFFFAOYSA-N | O |  |  |  |
| 218 | 23203 | ZHHYXNZJDGDGPJ-BSWSSELBSA-N | O |  |  |  |
| 219 | 25913 | PJLHTVIBELQURV-UHFFFAOYSA-N | O |  |  |  |
| 220 | 25916 | WHMWOHBXYIZFPF-UHFFFAOYSA-N | O |  |  |  |
| 221 | 26334 | JZBCTZLGKSYRSF-UHFFFAOYSA-N | O |  |  |  |
| 222 | 30084 | WGPCZPLRVAWXPW-UHFFFAOYSA-N | O |  |  |  |
| 223 | 30260 | PSJNLMVDUBCKQD-VBKZILBWSA-N | O |  |  |  |
| 224 | 30282 | KLTVSWGXIAYTHO-UHFFFAOYSA-N | O |  |  |  |
| 225 | 31289 | GYHFUZHODSMOHU-UHFFFAOYSA-N | O |  |  |  |
| 226 | 32594 | UXFSPRAGHGMRSQ-UHFFFAOYSA-N | O |  |  |  |
| 227 | 38896 | VOSRTXILOAOMLP-UHFFFAOYSA-N | O |  |  |  |
| 228 | 40063 | NCFVZZZXSIOSQB-UHFFFAOYSA-N |  |  | O |  |
| 229 | 40095 | YPWHZCPMOQGCDQ-HMGRVEAOSA-N | O |  |  |  |
| 230 | 40116 | BMIMEYWWZBBDCM-QHCPKHFHSA-N | O |  |  |  |
| 231 | 40119 | TVUGLERLRIQATC-BJMVGYQFSA-N |  |  | O |  |
| 232 | 40120 | HJFOOTRGDAPZMV-IJHZUMQISA-N |  |  |  | O |
| 233 | 40121 | ACCYCJOHUMRMMV-UOTUCCISSA-N |  |  |  | O |
| 234 | 40122 | WBQVRPYEEYUEBQ-JCJVTXLOSA-N |  | O |  |  |
| 235 | 40125 | KEABDZDFSMGRQX-RFYQBSPCSA-N | O |  |  |  |
| 236 | 40354 | RDUAJIJVNHKTQC-IROKYFGSSA-N | O |  |  |  |
| 237 | 40512 | HIOMEXREAUSUBP-MLXLLJIISA-N | O |  |  |  |
| 238 | 40532 | RSJKGSCJYJTIGS-UHFFFAOYSA-N | O |  |  |  |
| 239 | 46166 | KZTSESJJLOEXBX-SFHVURJKSA-N |  |  | O |  |
| 240 | 50138 | IKGXIBQEEMLURG-IEBISRBZSA-N |  |  | O |  |
| 241 | 50360 | QJTYCCFDQWFJHU-HMGRVEAOSA-N | O |  |  |  |
| 242 | 50490 | QGUHLWRUVKVMMA-LBPRGKRZSA-N | O |  |  |  |
| 243 | 50517 | RMBGWCMOTZYXDQ-UHFFFAOYSA-N | O |  |  |  |
| 244 | 57077 | VDYSHUXENHRSOO-XBXARRHUSA-N |  |  |  | O |
| 245 | 60253 | GDAAEAXMNLVRCZ-SFHVURJKSA-N |  |  | O |  |
| 246 | 60265 | CFCUNFSHJIQKLS-UHFFFAOYSA-N |  |  | O |  |
| 247 | 64832 | NLTOTZSPOYWSSP-UHFFFAOYSA-N |  |  | O |  |
| 248 | 70330 | YECJZKWIYHGDBM-SMRUOVSBSA-N | O |  |  |  |
| 249 | 70331 | UICKYSVCOIQHBZ-UGRLDDKXSA-N | O |  |  |  |
| 250 | 70334 | JUYBMOHJXUXKDN-AXCQTIHMSA-N | O |  |  |  |
| 251 | 70335 | FKRCODPIKNYEAC-UHFFFAOYSA-N | O |  |  |  |
| 252 | 70385 | JXJNPXPDKYRHJT-UHFFFAOYSA-N | O |  |  |  |
| 253 | 70387 | GTGRVHGFAZKYCM-UHFFFAOYSA-N | O |  |  |  |
| 254 | 70389 | DDTSMRSWKZCDKP-WLJGPMBKSA-N | O |  |  |  |
| 255 | 70390 | BDGGZGCEYOCZKY-CDCFCDKNSA-N | O |  |  |  |
| 256 | 70393 | BDGGZGCEYOCZKY-ZFDNWXCTSA-N | O |  |  |  |
| 257 | 70397 | WNIFXKPDILJURQ-JKPOUOEOSA-N | O |  |  |  |
| 258 | 70398 | CBGDCCSHOGQUSW-MDWZMJQESA-N |  | O |  |  |
| 259 | 70399 | NCPWFIVLKCFWSP-BSWSSELBSA-N | O |  |  |  |
| 260 | 70403 | SEYCNWBTCQVUFN-UHFFFAOYSA-N | O |  |  |  |
| 261 | 70412 | RZNUULHKDQNOPC-WMCRFIKSSA-N | O |  |  |  |
| 262 | 70413 | HUKRQFYAGWETIT-QSTOMECMSA-N | O |  |  |  |
| 263 | 70416 | CWRRYAWFDXCSDM-ASEDPRHOSA-N | O |  |  |  |
| 264 | 70417 | DSOHPFKCOCWTHP-UHFFFAOYSA-N | O |  |  |  |
| 265 | 70426 | ZHPPISIUKDGCPJ-UHFFFAOYSA-N | O |  |  |  |
| 266 | 73205 | SFQIGPZCFNTPOD-KRWDZBQOSA-N |  | O |  |  |
| 267 | 84995 | OVMFOVNOXASTPA-VYUBKLCTSA-N |  | O |  |  |
| 268 | 91047 | NPNUFJAVOOONJE-GFUGXAQUSA-N | O |  |  |  |
| 269 | 91937 | ILEIUTCVWLYZOM-UHFFFAOYSA-N | O |  |  |  |
| 270 | 91947 | PETRWTHZSKVLRE-UHFFFAOYSA-N | O |  |  |  |
| 271 | 92044 | PVFGJHYLIHMCQD-UHFFFAOYSA-N | O |  |  |  |
| 272 | 92059 | VEIYJWQZNGASMA-UHFFFAOYSA-N | O |  |  |  |
| 273 | 92078 | FPIOBTBNRZPWJW-UHFFFAOYSA-N | O |  |  |  |
| 274 | 92208 | WUADCCWRTIWANL-UHFFFAOYSA-N | O |  |  |  |
| 275 | 92244 | BERKLMUSNDCVHA-KSUOADGHSA-N | O |  |  |  |
| 276 | 92384 | RTATXGUCZHCSNG-QHWHWDPRSA-N |  | O |  |  |
| 277 | 92495 | OEIIVGQLDAYZNT-UHFFFAOYSA-N | O |  |  |  |
| 278 | 92910 | NVEQFIOZRFFVFW-RGCMKSIDSA-N | O |  |  |  |
| 279 | 93425 | JHJRJJRSKZLTFQ-AEZYHTSRSA-N | O |  |  |  |
| 280 | 93426 | WBQVRPYEEYUEBQ-BAWWYIFISA-N |  | O |  |  |
| 281 | 93427 | ACCYCJOHUMRMMV-HUWKTHDESA-N |  |  |  | O |
| 282 | 93428 | RMNUYAJCFQKSFY-CVZCIHLESA-N | O |  |  |  |
| 283 | 93429 | OEFZCYUPYXIEHQ-VLFNMTORSA-N | O |  |  |  |
| 284 | 93430 | KLASAGYILRZOSA-NPIIZBNNSA-N | O |  |  |  |
| 285 | 93431 | KLASAGYILRZOSA-CJDDOTGPSA-N | O |  |  |  |
| 286 | 93432 | WBQVRPYEEYUEBQ-ZZRFBVNQSA-N |  | O |  |  |
| 287 | 93567 | ICLVCWSZHUZEFT-QNDFHXLGSA-N | O |  |  |  |
| 288 | 94465 | KMOUJOKENFFTPU-QNDFHXLGSA-N | O |  |  |  |
| 289 | 94570 | FIAAVMJLAGNUKW-VQVVXJKKSA-N |  |  | O |  |
| 290 | 94609 | RTHZSGZAVUIROF-UHFFFAOYSA-N | O |  |  |  |
| 291 | 94714 | BJBUTJQYZDYRMJ-UHFFFAOYSA-N |  |  | O |  |
| 292 | 94715 | AEMOLEFTQBMNLQ-AQKNRBDQSA-N |  |  | O |  |
| 293 | 96542 | IHPKGUQCSIINRJ-CSKARUKUSA-N | O |  |  |  |
| 294 | 97063 | RUVINXPYWBROJD-ONEGZZNKSA-N |  |  | O |  |
| 295 | 97769 | HUMNYLRZRPPJDN-UHFFFAOYSA-N | O |  |  |  |
| 296 | 98191 | LHGVFZTZFXWLCP-UHFFFAOYSA-N | O |  |  |  |
| 297 | 98247 | UIDGLYUNOUKLBM-GEBJFKNCSA-N |  | O |  |  |
| 298 | 98270 | RFPGFYAPDRVXAE-UHFFFAOYSA-N | O |  |  |  |
| 299 | 98436 | RRAFCDWBNXTKKO-UHFFFAOYSA-N | O |  |  |  |
| 300 | 99219 | UOAVLLDKKZQJMK-RPBIRKHYSA-N | O |  |  |  |
| 301 | 109594 | QHJJASRUTXHRAL-UHFFFAOYSA-N | O |  |  |  |
| 302 | 111496 | CIJATEIGJFIOPE-OUOXKOSGSA-N |  |  |  | O |
| 303 | 113479 | BIVBRWYINDPWKA-VLQRKCJKSA-L | O |  |  |  |
| 304 | 119815 | DGKSRSQXQWIQTH-UHFFFAOYSA-N | O |  |  |  |
| 305 | 122851 | MOBCUWLJOZHPQL-UHFFFAOYSA-N |  | O |  |  |
| 306 | 124049 | DAWSYIQAGQMLFS-SFHVURJKSA-N |  |  | O |  |
| 307 | 124050 | CFWLRXJPRRCJTI-UHFFFAOYSA-N |  | O |  |  |
| 308 | 124052 | LBQIJVLKGVZRIW-ZDUSSCGKSA-N |  |  | O |  |
| 309 | 125777 | WECAUVIWKBEGRA-DHZHZOJOSA-N | O |  |  |  |
| 310 | 149821 | LPLVUJXQOOQHMX-MOGLOQIBSA-N |  | O |  |  |
| 311 | 153611 | MTFDQDUGEXYFJQ-QVBWJUSMSA-N | O |  |  |  |
| 312 | 163744 | KEIFOIQHFNUSQE-HDENPQJWSA-N |  | O |  |  |
| 313 | 173993 | HFTWTHSIMCSLFQ-CMLLUXCUSA-N | O |  |  |  |
| 314 | 176907 | JHYXBPPMXZIHKG-UHFFFAOYSA-N | O |  |  |  |
| 315 | 187587 | PHHAXWBLJNBVNS-UHFFFAOYSA-N |  |  | O |  |
| 316 | 190416 | LCRIQVFKVCYUAO-UHFFFAOYSA-N |  |  | O |  |
| 317 | 195343 | RETHOWGCGNZYSL-NNWQLOHHSA-N |  |  | O |  |
| 318 | 197678 | NEIURIYDQMKXIG-QHCPKHFHSA-N |  |  | O |  |
| 319 | 200215 | FYJCXNYYSACEJK-MRYIOYKESA-N | O |  |  |  |
| 320 | 200216 | JELPWVHINQKKQW-QGNLIWKUSA-N | O |  |  |  |
| 321 | 200217 | BOAZHQJZXAGAGU-DZHTZZIMSA-N | O |  |  |  |
| 322 | 200219 | CELHHQQOUSEVKY-ZAKIEJRYSA-N | O |  |  |  |
| 323 | 209991 | SWPKMTGYQGHLJS-DNGMOHDESA-N | O |  |  |  |
| 324 | 218027 | CGKWSLSAYABZTL-UHFFFAOYSA-N | O |  |  |  |
| 325 | 225689 | JFSHUTJDVKUMTJ-QHPUVITPSA-N | O |  |  |  |
| 326 | 237659 | VHWHNZUORPFYQI-UHFFFAOYSA-N | O |  |  |  |
| 327 | 237660 | WTNXJYOYGPGIJK-UHFFFAOYSA-N | O |  |  |  |
| 328 | 238782 | URFCJEUYXNAHFI-UHFFFAOYSA-N |  |  | O |  |
| 329 | 238934 | FXJPTJQFJYNFKC-UHFFFAOYSA-N | O |  |  |  |
| 330 | 246505 | MEHHCBRCXIDGKZ-UHFFFAOYSA-N | O |  |  |  |
| 331 | 257572 | GSZUGBAEBARHAW-YMQHIKHWSA-N | O |  |  |  |
| 332 | 275521 | SZNLKILVMCHHSD-OZFNKYQOSA-N | O |  |  |  |
| 333 | 278342 | OIHAASBKFLBOND-UMAQUBEUSA-N | O |  |  |  |
| 334 | 278344 | ZFSRTFFNWLQWAO-UEFFDLHQSA-N | O |  |  |  |
| 335 | 278364 | WPDHECQXVOACTR-RSNVNIAASA-N |  | O |  |  |
| 336 | 278365 | BBTZPUQIJXVLFI-HXBRCTIJSA-N | O |  |  |  |
| 337 | 278367 | XVYINQOBIANOMV-KEPWKZLGSA-N | O |  |  |  |
| 338 | 280862 | VJJZJBUCDWKPLC-UHFFFAOYSA-N | O |  |  |  |
| 339 | 281255 | DUWPGRAKHMEPCM-IZZDOVSWSA-N |  | O |  |  |
| 340 | 281417 | XHCADAYNFIFUHF-LJNLQMBESA-N | O |  |  |  |
| 341 | 281426 | ORHBXUUXSCNDEV-UHFFFAOYSA-N |  |  |  | O |
| 342 | 281797 | MMPVAPMCVABQPS-UHFFFAOYSA-N | O |  |  |  |
| 343 | 281814 | KIMDVVKVNNSHGZ-UHFFFAOYSA-N | O |  |  |  |
| 344 | 281855 | AFSDNFLWKVMVRB-UHFFFAOYSA-N | O |  |  |  |
| 345 | 283349 | JZQKTMZYLHNFPL-UHFFFAOYSA-N | O |  |  |  |
| 346 | 291875 | NRUOYYDQBWDRKE-UHFFFAOYSA-N | O |  |  |  |
| 347 | 300793 | YKUCHDXIBAQWSF-UHFFFAOYSA-N | O |  |  |  |
| 348 | 301249 | LXCMYLCNDJHAFX-UHFFFAOYSA-N | O |  |  |  |
| 349 | 301407 | YEROQRRJBUNBOY-HSZRJFAPSA-N | O |  |  |  |
| 350 | 301665 | RDYZHQQZLIBKBP-WEVVVXLNSA-N | O |  |  |  |
| 351 | 301770 | ILEDWLMCKZNDJK-UHFFFAOYSA-N | O |  |  |  |
| 352 | 301858 | DLIKSSGEMUFQOK-SFTVRKLSSA-N | O |  |  |  |
| 353 | 302012 | FNSFANUGPIQSTR-UHFFFAOYSA-N | O |  |  |  |
| 354 | 302525 | BICPGUILWBQAEY-GORDUTHDSA-N | O |  |  |  |
| 355 | 310283 | JCGXIYQLRYPHDG-DQOTWGJISA-N |  | O |  |  |
| 356 | 315125 | CQVDOHIEYGNTQX-UHFFFAOYSA-N |  |  |  | O |
| 357 | 315126 | WOMWVGHYSNATOB-UHFFFAOYSA-N |  | O |  |  |
| 358 | 316952 | GUWHMEMJBCLEBP-WJTDDFOZSA-N |  |  | O |  |
| 359 | 317480 | YGCCASGFIOIXIN-UHFFFAOYSA-N |  | O |  |  |
| 360 | 317481 | UCKSAYIMWMIZQJ-QDEBKDIKSA-N |  | O |  |  |
| 361 | 317482 | YURHIASRSSMERJ-UHFFFAOYSA-N |  |  |  | O |
| 362 | 317762 | OTJMSWBNEUNNEW-UHFFFAOYSA-N |  |  |  | O |
| 363 | 317777 | GRMSSCUVELGNHC-UHFFFAOYSA-N |  | O |  |  |
| 364 | 318585 | PGCKDCPTJAQQSQ-UHFFFAOYSA-N |  | O |  |  |
| 365 | 318619 | YAYJWUXAGVZHGX-MIUGBVLSSA-N |  | O |  |  |
| 366 | 318991 | FMKHMNBODOXQLQ-UXBLZVDNSA-N | O |  |  |  |
| 367 | 319001 | GAUFLNQQCSXBPK-UHFFFAOYSA-N |  | O |  |  |
| 368 | 319013 | GGWMNTNDTRKETA-UHFFFAOYSA-N |  | O |  |  |
| 369 | 319664 | ZZAIPFIGEGQNHP-UHFFFAOYSA-N |  | O |  |  |
| 370 | 320083 | LWESBHWAOZORCQ-UHFFFAOYSA-N |  | O |  |  |
| 371 | 320238 | LTSJTDDQUOUKJT-XTQSDGFTSA-N | O |  |  |  |
| 372 | 338211 | PPBISUGOQDBBEL-UHFFFAOYSA-N |  |  | O |  |
| 373 | 349817 | GLDVIKFETPAZNV-UHFFFAOYSA-N |  |  | O |  |
| 374 | 361658 | SSDIPYMSXRNGMZ-UHFFFAOYSA-N |  |  | O |  |
| 375 | 366074 | POIARNZEYGURDG-FNORWQNLSA-N | O |  |  |  |
| 376 | 422758 | TWBWSPDILHVKEV-RSPRXDBDSA-N |  |  | O |  |
| 377 | 423265 | DBMJMQXJHONAFJ-UHFFFAOYSA-M | O |  |  |  |
| 378 | 427657 | QOWFWSZIEKUOSQ-UHFFFAOYSA-N | O |  |  |  |
| 379 | 438092 | BWFSBUVPIAIXKJ-QHHAFSJGSA-N | O |  |  |  |
| 380 | 441251 | AZPLXDBZIQMMMT-UHFFFAOYSA-N | O |  |  |  |
| 381 | 442342 | UDGKKUWYNITJRX-UHFFFAOYSA-N | O |  |  |  |
| 382 | 442411 | JSHJOTDLYIHRKJ-VQTJNVASSA-N |  |  | O |  |
| 383 | 442433 | VMMVZVPAYFZNBM-KVFWHIKKSA-N | O |  |  |  |
| 384 | 442665 | MVOUGOXRXQDXDC-RSPRXDBDSA-N |  |  | O |  |
| 385 | 442774 | HZHXMXSXYQCAIG-KRWDZBQOSA-N |  | O |  |  |
| 386 | 442813 | MGJLSBDCWOSMHL-MIUGBVLSSA-N |  | O |  |  |
| 387 | 442822 | AALISTBXLBQUEH-UHFFFAOYSA-N | O |  |  |  |
| 388 | 450959 | MEADLGUPYQNUNF-BIIKFXOESA-N |  | O |  |  |
| 389 | 452864 | WPDHECQXVOACTR-KSHPUXFISA-N |  | O |  |  |
| 390 | 454316 | MMDUKUSNQNWVET-VYUBKLCTSA-N |  | O |  |  |
| 391 | 463991 | CQLRUIIRRZYHHS-OMUJUXNISA-N | O |  |  |  |
| 392 | 473311 | RETRVWFVEFCGOK-RMKNXTFCSA-N |  |  | O |  |
| 393 | 479675 | SSHDNSCEQSPWIM-FVTWEACWSA-N |  | O |  |  |
| 394 | 480770 | HCBKENVWCDLQOA-UHFFFAOYSA-N |  | O |  |  |
| 395 | 480774 | NGGYSPUAKQMTNP-UHFFFAOYSA-N |  |  | O |  |
| 396 | 480777 | DKVBYQAVNNRVNN-UHFFFAOYSA-N |  | O |  |  |
| 397 | 480780 | WLPHLDLTTPUDSI-UHFFFAOYSA-N |  |  | O |  |
| 398 | 480786 | RIWDYFGEQJAMKI-UHFFFAOYSA-N |  |  |  | O |
| 399 | 480787 | FWWGXZYUURXJLK-UHFFFAOYSA-N |  | O |  |  |
| 400 | 480815 | QFAPONVNJTUMHF-UHFFFAOYSA-N |  |  | O |  |
| 401 | 480854 | LAQLCZKPJGMFRM-BJKOFHAPSA-N |  | O |  |  |
| 402 | 480859 | RCZMWVKBVFOCEE-ZDUSSCGKSA-N |  |  | O |  |
| 403 | 480860 | DDMAUIOCNQXFHL-AWEZNQCLSA-N |  |  | O |  |
| 404 | 480863 | JRVDUBFSQWHYRJ-INIZCTEOSA-N |  |  | O |  |
| 405 | 480872 | WIEKYGJSRGBBTQ-AFMDSPMNSA-N |  |  | O |  |
| 406 | 481234 | KIZPADOTOCPASX-UHFFFAOYSA-N |  | O |  |  |
| 407 | 481235 | QQUXNFZAFOMGTQ-UHFFFAOYSA-N | O |  |  |  |
| 408 | 481949 | CCUZOQMNQONIMX-UHFFFAOYSA-N |  |  | O |  |
| 409 | 481963 | XHCCZOWAHHBCAK-UHFFFAOYSA-N |  |  | O |  |
| 410 | 481964 | TVMHBSODLWMMMV-UHFFFAOYSA-N |  |  | O |  |
| 411 | 481965 | VVXGFIWLFZMBCJ-UHFFFAOYSA-N | O |  |  |  |
| 412 | 487298 | PYVPKOSKOWDDSV-UHFFFAOYSA-N | O |  |  |  |
| 413 | 503731 | CNPMAFLUEHEXRE-UHFFFAOYSA-N |  | O |  |  |
| 414 | 509245 | LPEPZZAVFJPLNZ-UHFFFAOYSA-N | O |  |  |  |
| 415 | 524410 | XQWFHGOIUZFQPJ-LXGDFETPSA-N |  | O |  |  |
| 416 | 524411 | HJBUYKZTEBZNSH-ZRWXNEIDSA-N |  | O |  |  |
| 417 | 531730 | NNCFAUGCNTZUIW-UHFFFAOYSA-N |  |  | O |  |
| 418 | 547113 | ZCVDLJXIVVGRBZ-UHFFFAOYSA-N | O |  |  |  |
| 419 | 550385 | YKYNYMQDXOWMDT-UHFFFAOYSA-N | O |  |  |  |
| 420 | 591148 | QNLGNISMYMFVHP-UHFFFAOYSA-N | O |  |  |  |
| 421 | 591149 | LXLBPEZCZLGOGJ-UHFFFAOYSA-N | O |  |  |  |
| 422 | 595178 | IEJGJZLXEWWZHI-UHFFFAOYSA-N | O |  |  |  |
| 423 | 596309 | CUFAXDWQDQQKFF-DEOSSOPVSA-N |  |  | O |  |
| 424 | 599072 | DTMJARJUCFBMFR-ZHGMGIEYSA-N |  |  | O |  |
| 425 | 600135 | WLDXQYSLYHUZTM-UHFFFAOYSA-N | O |  |  |  |
| 426 | 604077 | WSOHPJFMARQRFD-UHFFFAOYSA-N |  |  | O |  |
| 427 | 604078 | DDLPIQXHEKZHQX-UHFFFAOYSA-N |  |  | O |  |
| 428 | 604080 | STFVTZQCNYBLNE-UHFFFAOYSA-N |  |  | O |  |
| 429 | 607541 | WRNYEZGVIHDIGH-YRNVUSSQSA-N | O |  |  |  |
| 430 | 607642 | FFPFIAFZJXSGDK-XKZIYDEJSA-N |  |  | O |  |
| 431 | 607807 | KDXWZGOCBQGWEB-ISCAJFQMSA-N | O |  |  |  |
| 432 | 607808 | KDXWZGOCBQGWEB-QZWSAEFYSA-N | O |  |  |  |
| 433 | 607809 | GSIREHLZHMQJNR-WAGMDUJWSA-N | O |  |  |  |
| 434 | 607810 | GSIREHLZHMQJNR-YYYDHYCISA-N | O |  |  |  |
| 435 | 607811 | GNJDLKNJCWOSBO-LKYFMKHGSA-N |  |  | O |  |
| 436 | 607996 | NMDAXWXNNIQNFH-ISAWABDASA-N | O |  |  |  |
| 437 | 618087 | CWRRYAWFDXCSDM-RGBHSECDSA-N | O |  |  |  |
| 438 | 630492 | JOMJKDWBAPDZIF-UHFFFAOYSA-N | O |  |  |  |
| 439 | 643731 | HZYHMHHBBBSGHB-ODYTWBPASA-N | O |  |  |  |
| 440 | 644267 | QJKMIJNRNRLQSS-WEVVVXLNSA-N |  |  | O |  |
| 441 | 664151 | KKFAKKIIFUFASS-UHFFFAOYSA-N | O |  |  |  |
| 442 | 666840 | SVHCNENPWOPFOI-AVRWGWEMSA-N |  |  | O |  |
| 443 | 742117 | VAWLLIOUAFRMHN-UHFFFAOYSA-N | O |  |  |  |
| 444 | 742118 | VDOHBGQSFOWYTB-UHFFFAOYSA-N | O |  |  |  |
| 445 | 751571 | NGWKGSCSHDHHAJ-YPFQVHCOSA-N |  | O |  |  |
| 446 | 752447 | MKDSBDQLSLPNOQ-OZFAJWBBSA-N |  | O |  |  |
| 447 | 783899 | BNTYXDBRBRYHOP-UHFFFAOYSA-N |  |  |  | O |
| 448 | 818598 | AEAIWNGAMDGGNB-UHFFFAOYSA-N |  |  |  | O |
| 449 | 818599 | DPLWUTYEBRKBLI-UHFFFAOYSA-N |  |  |  | O |
| 450 | 840805 | WBDNTJSRHDSPSR-KPKJPENVSA-N |  |  | O |  |
| 451 | 862769 | NXBYIJSAISXPKJ-WEVVVXLNSA-N |  |  | O |  |
| 452 | 881804 | TUHJQMZJOMZXJO-XVNBXDOJSA-N |  |  | O |  |
| 453 | 904276 | FSHPJPOJLGCQOJ-UHFFFAOYSA-N |  | O |  |  |
| 454 | 905172 | OBFDSOYVKVRFGY-JEHLDPHZSA-N | O |  |  |  |
| 455 | 905173 | PDSBBERBJSPJMP-JNPZOHSNSA-N | O |  |  |  |
| 456 | 911308 | SLQRKKBBWHNDFD-UHFFFAOYSA-N | O |  |  |  |
| 457 | 911463 | PEUWWOCVYRAQEV-UHFFFAOYSA-N | O |  |  |  |
| 458 | 912581 | KCUZCRLRQVRBBV-UHFFFAOYSA-N |  | O |  |  |
| 459 | 938903 | BYQKEKUAWGGZTQ-PMIHXJGWSA-N |  |  | O |  |
| 460 | 938904 | DBMYJNREMDOYPY-XMCJHVAISA-N | O |  |  |  |
| 461 | 939210 | TUUBGLDJKKCMRH-LDSFXQROSA-N |  | O |  |  |
| 462 | 999902 | SBSQRDFJISUOGV-UHFFFAOYSA-N | O |  |  |  |

**Table S4 List of licorice compounds in TCMSP and their existence in TM-MC and TCMID.**

| No | ID | INCHIKEY | Only TCMSP | TCMSP, TM-MC, and TCMID | TCMSP and TM-MC | TCMSP and TCMID |
| --- | --- | --- | --- | --- | --- | --- |
| 1 | MOL000012 | VKOBVWXKNCXXDE-UHFFFAOYSA-N | O |  |  |  |
| 2 | MOL000040 | RODXRVNMMDRFIK-UHFFFAOYSA-N | O |  |  |  |
| 3 | MOL000057 | MGWAVDBGNNKXQV-UHFFFAOYSA-N | O |  |  |  |
| 4 | MOL000098 | REFJWTPEDVJJIY-UHFFFAOYSA-N |  |  | O |  |
| 5 | MOL000105 | YQUVCSBJEUQKSH-UHFFFAOYSA-N | O |  |  |  |
| 6 | MOL000118 | WUOACPNHFRMFPN-SECBINFHSA-N | O |  |  |  |
| 7 | MOL000211 | QGJZLNKBHJESQX-FZFNOLFKSA-N | O |  |  |  |
| 8 | MOL000239 | BJBUTJQYZDYRMJ-UHFFFAOYSA-N |  |  | O |  |
| 9 | MOL000263 | MIJYXULNPSFWEK-GTOFXWBISA-N |  |  | O |  |
| 10 | MOL000354 | IZQSVPBOUDKVDZ-UHFFFAOYSA-N | O |  |  |  |
| 11 | MOL000359 | KZJWDPNRJALLNS-ZFVHJZABSA-N |  | O |  |  |
| 12 | MOL000391 | MGJLSBDCWOSMHL-MIUGBVLSSA-N |  | O |  |  |
| 13 | MOL000392 | HKQYGTCOTHHOMP-UHFFFAOYSA-N |  | O |  |  |
| 14 | MOL000415 | IKGXIBQEEMLURG-NVPNHPEKSA-N |  | O |  |  |
| 15 | MOL000417 | ZZAJQOPSWWVMBI-UHFFFAOYSA-N |  |  | O |  |
| 16 | MOL000422 | IYRMWMYZSQPJKC-UHFFFAOYSA-N |  |  | O |  |
| 17 | MOL000437 | OVSQVDMCBVZWGM-QSOFNFLRSA-N |  |  | O |  |
| 18 | MOL000445 | UCHYSPNEUSDFQR-UHFFFAOYSA-N |  |  | O |  |
| 19 | MOL000467 | KJGPBYUQZLUKLL-UHFFFAOYSA-N | O |  |  |  |
| 20 | MOL000475 | RUVINXPYWBROJD-ONEGZZNKSA-N |  |  | O |  |
| 21 | MOL000486 | KQMVAGISDHMXJJ-UHFFFAOYSA-N | O |  |  |  |
| 22 | MOL000497 | KAZSKMJFUPEHHW-DHZHZOJOSA-N |  | O |  |  |
| 23 | MOL000500 | XRVFNNUXNVWYTI-LLVKDONJSA-N |  |  | O |  |
| 24 | MOL000511 | WCGUUGGRBIKTOS-GPOJBZKASA-N | O |  |  |  |
| 25 | MOL000561 | JPUKWEQWGBDDQB-QSOFNFLRSA-N |  | O |  |  |
| 26 | MOL000668 | YVBAUDVGOFCUSG-UHFFFAOYSA-N | O |  |  |  |
| 27 | MOL000671 | NOOLISFMXDJSKH-AEJSXWLSSA-N | O |  |  |  |
| 28 | MOL000676 | DOIRQSBPFJWKBE-UHFFFAOYSA-N | O |  |  |  |
| 29 | MOL000703 | CATSNJVOTSVZJV-UHFFFAOYSA-N | O |  |  |  |
| 30 | MOL000705 | FXHGMKSSBGDXIY-UHFFFAOYSA-N | O |  |  |  |
| 31 | MOL001097 | CTQNGGLPUBDAKN-UHFFFAOYSA-N | O |  |  |  |
| 32 | MOL001098 | IVSZLXZYQVIEFR-UHFFFAOYSA-N | O |  |  |  |
| 33 | MOL001099 | URLKBWYHVLBVBO-UHFFFAOYSA-N | O |  |  |  |
| 34 | MOL001484 | HUKSJTUUSUGIDC-ZBEGNZNMSA-N | O |  |  |  |
| 35 | MOL001543 | FIAAVMJLAGNUKW-VQVVXJKKSA-N |  |  | O |  |
| 36 | MOL001599 | BOBTWVZHSPRDFC-OUCADQQQSA-N | O |  |  |  |
| 37 | MOL001696 | XFFOMNJIDRDDLQ-UHFFFAOYSA-N | O |  |  |  |
| 38 | MOL001737 | KMAKOBLIOCQGJP-UHFFFAOYSA-N | O |  |  |  |
| 39 | MOL001789 | DXDRHHKMWQZJHT-FPYGCLRLSA-N |  | O |  |  |
| 40 | MOL001792 | FURUXTVZLHCCNA-AWEZNQCLSA-N |  | O |  |  |
| 41 | MOL001850 | HJOVHMDZYOCNQW-UHFFFAOYSA-N | O |  |  |  |
| 42 | MOL002137 | TVMXDCGIABBOFY-UHFFFAOYSA-N | O |  |  |  |
| 43 | MOL002166 | GXDHCNNESPLIKD-UHFFFAOYSA-N | O |  |  |  |
| 44 | MOL002198 | IMNFDUFMRHMDMM-UHFFFAOYSA-N | O |  |  |  |
| 45 | MOL002311 | LWESBHWAOZORCQ-UHFFFAOYSA-N |  | O |  |  |
| 46 | MOL002547 | LGNSZMLHOYDATP-YIZRAAEISA-N | O |  |  |  |
| 47 | MOL002565 | NSRJSISNDPOJOP-BBRMVZONSA-N |  |  | O |  |
| 48 | MOL002678 | YNQLUTRBYVCPMQ-UHFFFAOYSA-N | O |  |  |  |
| 49 | MOL002693 | RTATXGUCZHCSNG-QHWHWDPRSA-N |  | O |  |  |
| 50 | MOL002844 | URFCJEUYXNAHFI-ZDUSSCGKSA-N |  |  | O |  |
| 51 | MOL002850 | NLZUEZXRPGMBCV-UHFFFAOYSA-N | O |  |  |  |
| 52 | MOL002943 | LRHPLDYGYMQRHN-UHFFFAOYSA-N | O |  |  |  |
| 53 | MOL003218 | NACBYUYHTLUWAI-UHFFFAOYSA-N |  |  |  | O |
| 54 | MOL003656 | YGCCASGFIOIXIN-UHFFFAOYSA-N |  | O |  |  |
| 55 | MOL003662 | LCAWNFIFMLXZPQ-UHFFFAOYSA-N |  |  | O |  |
| 56 | MOL003686 | UIDGLYUNOUKLBM-GEBJFKNCSA-N |  | O |  |  |
| 57 | MOL003896 | XRGWZIGGCNSFRY-UHFFFAOYSA-N | O |  |  |  |
| 58 | MOL003985 | RAHRTJHAYXOITA-DTWKUNHWSA-N | O |  |  |  |
| 59 | MOL004328 | FTVWIRXFELQLPI-ZDUSSCGKSA-N |  |  | O |  |
| 60 | MOL004385 | PFQMUQWFRINBBG-UHFFFAOYSA-N |  |  | O |  |
| 61 | MOL004589 | JVSWJIKNEAIKJW-UHFFFAOYSA-N | O |  |  |  |
| 62 | MOL004723 | SCWPFSIZUZUCCE-UHFFFAOYSA-N | O |  |  |  |
| 63 | MOL004801 | IVCITHFPTPZRQC-IMWGDMARSA-N |  |  | O |  |
| 64 | MOL004802 | LXDQSUZPIXBWIQ-BQYQJAHWSA-N | O |  |  |  |
| 65 | MOL004803 | LAQLCZKPJGMFRM-BJKOFHAPSA-N |  | O |  |  |
| 66 | MOL004804 | MPDGHEJMBKOTSU-YKLVYJNSSA-N |  | O |  |  |
| 67 | MOL004805 | NEIURIYDQMKXIG-QHCPKHFHSA-N |  |  | O |  |
| 68 | MOL004806 | LOMFVXOLTBGKBK-CVUHLLMESA-N | O |  |  |  |
| 69 | MOL004807 | AEMOLEFTQBMNLQ-QIUUJYRFSA-N |  |  | O |  |
| 70 | MOL004808 | PDKHNRSRVURSHL-HNNXBMFYSA-N | O |  |  |  |
| 71 | MOL004809 | OJMSZMQLRQBGLS-UHFFFAOYSA-N | O |  |  |  |
| 72 | MOL004810 | CFCUNFSHJIQKLS-ZDUSSCGKSA-N |  |  | O |  |
| 73 | MOL004811 | RCZMWVKBVFOCEE-ZDUSSCGKSA-N |  |  | O |  |
| 74 | MOL004812 | DDMAUIOCNQXFHL-CQSZACIVSA-N |  |  | O |  |
| 75 | MOL004813 | RMIHCTUWJGVJQB-OAHLLOKOSA-N |  |  | O |  |
| 76 | MOL004814 | OVLUQDOJWGTPHA-UHFFFAOYSA-N |  | O |  |  |
| 77 | MOL004815 | TUHJQMZJOMZXJO-XVNBXDOJSA-N |  |  | O |  |
| 78 | MOL004816 | DKIYWPRXYDNQFG-XMMPIXPASA-N |  |  | O |  |
| 79 | MOL004817 | UWUOGPWSIVRQNM-UHFFFAOYSA-N | O |  |  |  |
| 80 | MOL004818 | MTISFNYJKNLZEN-UHFFFAOYSA-N | O |  |  |  |
| 81 | MOL004819 | HKELXUISPZEXHA-UHFFFAOYSA-N | O |  |  |  |
| 82 | MOL004820 | VFIXONREXXFDQV-UHFFFAOYSA-N | O |  |  |  |
| 83 | MOL004821 | KZTSESJJLOEXBX-GOSISDBHSA-N |  |  | O |  |
| 84 | MOL004822 | TVUGLERLRIQATC-BJMVGYQFSA-N |  |  | O |  |
| 85 | MOL004823 | IOKJHFYJRMTPMJ-UGKGYDQZSA-N | O |  |  |  |
| 86 | MOL004824 | GAUFLNQQCSXBPK-SFHVURJKSA-N |  | O |  |  |
| 87 | MOL004825 | FFPFIAFZJXSGDK-XKZIYDEJSA-N |  |  | O |  |
| 88 | MOL004827 | LWZACZCRAUQSLH-UHFFFAOYSA-N |  |  | O |  |
| 89 | MOL004828 | WCSHKPNHOSDFGK-UHFFFAOYSA-N | O |  |  |  |
| 90 | MOL004829 | ATJOIGKHVRPLSM-RBUKOAKNSA-N |  |  | O |  |
| 91 | MOL004830 | QTYUSOHYEPOHLV-FNORWQNLSA-N | O |  |  |  |
| 92 | MOL004831 | CBGDCCSHOGQUSW-MDWZMJQESA-N |  | O |  |  |
| 93 | MOL004832 | XSIFPSYPOVKYCO-UHFFFAOYSA-N | O |  |  |  |
| 94 | MOL004833 | UUJBHSNXZMGYBT-ZDUSSCGKSA-N |  |  |  | O |
| 95 | MOL004834 | CXCORJXYCPSBSK-UHFFFAOYSA-N | O |  |  |  |
| 96 | MOL004835 | CTBBBAQHUJVKNG-UXBLZVDNSA-N | O |  |  |  |
| 97 | MOL004836 | QJKMIJNRNRLQSS-UITAMQMPSA-N |  |  | O |  |
| 98 | MOL004837 | NZZIMKJIVMHWJC-UHFFFAOYSA-N |  |  | O |  |
| 99 | MOL004838 | SJIZTMNAKZAOTA-UHFFFAOYSA-N |  |  | O |  |
| 100 | MOL004839 | RIRARCHMRDHZAR-BQBZGAKWSA-N | O |  |  |  |
| 101 | MOL004840 | KRWXHCWICDLYOY-UHFFFAOYSA-N |  | O |  |  |
| 102 | MOL004841 | DRDRYGIIYOPBBZ-XBXARRHUSA-N |  |  | O |  |
| 103 | MOL004842 | WBDNTJSRHDSPSR-GHXNOFRVSA-N |  |  | O |  |
| 104 | MOL004843 | RETRVWFVEFCGOK-TWGQIWQCSA-N |  |  | O |  |
| 105 | MOL004844 | CUFAXDWQDQQKFF-XMMPIXPASA-N |  |  | O |  |
| 106 | MOL004845 | RETHOWGCGNZYSL-AVZKMJBFSA-N |  |  | O |  |
| 107 | MOL004847 | CXOWYJMDMMMMJO-UHFFFAOYSA-N | O |  |  |  |
| 108 | MOL004848 | UYRRMZSSCNIGNC-RMKNXTFCSA-N | O |  |  |  |
| 109 | MOL004849 | LCRIQVFKVCYUAO-UHFFFAOYSA-N |  |  | O |  |
| 110 | MOL004850 | NGWKGSCSHDHHAJ-FZEUIZPTSA-N |  | O |  |  |
| 111 | MOL004851 | HJGURBGBPIKRER-UHFFFAOYSA-N |  | O |  |  |
| 112 | MOL004852 | GLDVIKFETPAZNV-UHFFFAOYSA-N |  |  | O |  |
| 113 | MOL004853 | TVMHBSODLWMMMV-UHFFFAOYSA-N |  |  | O |  |
| 114 | MOL004855 | GGWMNTNDTRKETA-UHFFFAOYSA-N |  | O |  |  |
| 115 | MOL004856 | JQNSUDIGIIGIOL-UHFFFAOYSA-N |  |  |  | O |
| 116 | MOL004857 | YQEPOQVRUDADPH-UHFFFAOYSA-N |  |  |  | O |
| 117 | MOL004858 | MEADLGUPYQNUNF-BIIKFXOESA-N |  | O |  |  |
| 118 | MOL004859 | JXPOLSKBTUYKJB-QMMMGPOBSA-N | O |  |  |  |
| 119 | MOL004860 | GNJDLKNJCWOSBO-VIGBHHERSA-N |  |  | O |  |
| 120 | MOL004861 | UCKSAYIMWMIZQJ-QDEBKDIKSA-N |  | O |  |  |
| 121 | MOL004862 | HCBKENVWCDLQOA-JOCHJYFZSA-N |  | O |  |  |
| 122 | MOL004863 | WSOHPJFMARQRFD-UHFFFAOYSA-N |  |  | O |  |
| 123 | MOL004864 | DDLPIQXHEKZHQX-UHFFFAOYSA-N |  |  | O |  |
| 124 | MOL004865 | STFVTZQCNYBLNE-UHFFFAOYSA-N |  |  | O |  |
| 125 | MOL004866 | AFJYQKPCJLMHCC-UHFFFAOYSA-N | O |  |  |  |
| 126 | MOL004867 | OCIIFJFJVOTFTN-UHFFFAOYSA-N | O |  |  |  |
| 127 | MOL004868 | WGNIVAMNAWBYRO-UHFFFAOYSA-N |  |  | O |  |
| 128 | MOL004869 | QFAPONVNJTUMHF-UHFFFAOYSA-N |  |  | O |  |
| 129 | MOL004870 | VLJOWGMLRJTNDQ-UHFFFAOYSA-N | O |  |  |  |
| 130 | MOL004871 | WGECXQBGLLYSFP-ZETCQYMHSA-N | O |  |  |  |
| 131 | MOL004872 | OWBYJSNJVWQEQX-HSZRJFAPSA-N | O |  |  |  |
| 132 | MOL004873 | YJJXCOSDPIJFJR-UHFFFAOYSA-N | O |  |  |  |
| 133 | MOL004874 | UEXOPXIMQJMWKA-UHFFFAOYSA-N | O |  |  |  |
| 134 | MOL004875 | SSDIPYMSXRNGMZ-UHFFFAOYSA-N |  |  | O |  |
| 135 | MOL004876 | LPLVUJXQOOQHMX-QWBHMCJMSA-N |  | O |  |  |
| 136 | MOL004877 | GBRZTUJCDFSIHM-KRWDZBQOSA-N |  | O |  |  |
| 137 | MOL004878 | NZYSZZDSYIBYLC-UHFFFAOYSA-N |  | O |  |  |
| 138 | MOL004879 | FWWGXZYUURXJLK-UHFFFAOYSA-N |  | O |  |  |
| 139 | MOL004880 | ZBIKAJHAGKBFKR-UHFFFAOYSA-N |  |  |  | O |
| 140 | MOL004881 | NXBYIJSAISXPKJ-WEVVVXLNSA-N |  |  | O |  |
| 141 | MOL004882 | CNPMAFLUEHEXRE-UHFFFAOYSA-N |  | O |  |  |
| 142 | MOL004883 | KCUZCRLRQVRBBV-UHFFFAOYSA-N |  | O |  |  |
| 143 | MOL004884 | KIZPADOTOCPASX-UHFFFAOYSA-N |  | O |  |  |
| 144 | MOL004885 | JNDPLDZUOFZXIG-ZDUSSCGKSA-N |  | O |  |  |
| 145 | MOL004886 | WPDHECQXVOACTR-MOBXHYNLSA-N |  | O |  |  |
| 146 | MOL004888 | SUOZCCLVZRHGRK-ODRPJRBQSA-N |  | O |  |  |
| 147 | MOL004890 | HDGQICNBXPAKLR-QMMMGPOBSA-N | O |  |  |  |
| 148 | MOL004891 | QGPHRCQDTPCIQI-KXBFYZLASA-N | O |  |  |  |
| 149 | MOL004892 | WBQVRPYEEYUEBQ-QCNHKOKZSA-N |  | O |  |  |
| 150 | MOL004894 | LPLVUJXQOOQHMX-JWHFFEPQSA-N |  | O |  |  |
| 151 | MOL004896 | SLWCVFLNZDOMEZ-BOJTYEKNSA-N |  | O |  |  |
| 152 | MOL004898 | XHTOWVVXFDSBGH-XBXARRHUSA-N |  |  | O |  |
| 153 | MOL004899 | BCNKILSUUHWRTG-WTRZSJPGSA-N |  | O |  |  |
| 154 | MOL004900 | IPBGQSWRSQPGCF-BYWKWHLKSA-N |  | O |  |  |
| 155 | MOL004902 | JCGXIYQLRYPHDG-NNURFOPQSA-N |  | O |  |  |
| 156 | MOL004903 | DEMKZLAVQYISIA-ZRWXNEIDSA-N |  | O |  |  |
| 157 | MOL004904 | MOBCUWLJOZHPQL-OAQYLSRUSA-N |  | O |  |  |
| 158 | MOL004905 | MKDSBDQLSLPNOQ-YDJDNKAXSA-N |  | O |  |  |
| 159 | MOL004906 | CJUFYKORDZSOLF-INIZCTEOSA-N |  | O |  |  |
| 160 | MOL004907 | GRMSSCUVELGNHC-UHFFFAOYSA-N |  | O |  |  |
| 161 | MOL004908 | LBQIJVLKGVZRIW-ZDUSSCGKSA-N |  |  | O |  |
| 162 | MOL004909 | SSHDNSCEQSPWIM-RBBQGFHWSA-N |  | O |  |  |
| 163 | MOL004910 | DAWSYIQAGQMLFS-SFHVURJKSA-N |  |  | O |  |
| 164 | MOL004911 | NGGYSPUAKQMTNP-UHFFFAOYSA-N |  |  | O |  |
| 165 | MOL004912 | COLMVFWKLOZOOP-UHFFFAOYSA-N |  |  | O |  |
| 166 | MOL004913 | AAKHRTZXSZBLFQ-UHFFFAOYSA-N |  |  | O |  |
| 167 | MOL004914 | FBPDUHIOILHCFT-UHFFFAOYSA-N | O |  |  |  |
| 168 | MOL004915 | NNCFAUGCNTZUIW-UHFFFAOYSA-N |  |  | O |  |
| 169 | MOL004916 | CABCROIGBFQLHZ-CYBMUJFWSA-N | O |  |  |  |
| 170 | MOL004917 | TUUBGLDJKKCMRH-UXSCNWBASA-N |  | O |  |  |
| 171 | MOL004918 | VLKZOEOYAKHREP-UHFFFAOYSA-N | O |  |  |  |
| 172 | MOL004919 | UAEPNZWRGJTJPN-UHFFFAOYSA-N | O |  |  |  |
| 173 | MOL004920 | GDOPTJXRTPNYNR-UHFFFAOYSA-N | O |  |  |  |
| 174 | MOL004921 | GUWHMEMJBCLEBP-BZZOAKBMSA-N |  |  | O |  |
| 175 | MOL004922 | URCFDWVAWCRKTQ-CYBMUJFWSA-N | O |  |  |  |
| 176 | MOL004924 | PVEMGMOWXQUWRD-NJAOXFEXSA-N |  |  | O |  |
| 177 | MOL004925 | SGEWCQFRYRRZDC-VPRICQMDSA-N |  |  | O |  |
| 178 | MOL004926 | KSDSYIXRWHRPMN-SFTVRKLSSA-N |  |  | O |  |
| 179 | MOL004927 | HZHXMXSXYQCAIG-KRWDZBQOSA-N |  | O |  |  |
| 180 | MOL004928 | MVOUGOXRXQDXDC-ONIDZEHXSA-N |  |  | O |  |
| 181 | MOL004929 | REIUXOLGHVXAEO-UHFFFAOYSA-N | O |  |  |  |
| 182 | MOL004930 | WOMWVGHYSNATOB-UHFFFAOYSA-N |  | O |  |  |
| 183 | MOL004931 | VCKYLOIMXUHPDA-UHFFFAOYSA-N |  |  |  | O |
| 184 | MOL004932 | LPLVUJXQOOQHMX-MOGLOQIBSA-N |  | O |  |  |
| 185 | MOL004933 | KEIFOIQHFNUSQE-LSPCXFAPSA-N |  | O |  |  |
| 186 | MOL004934 | AFABGHUZZDYHJO-UHFFFAOYSA-N | O |  |  |  |
| 187 | MOL004935 | SFQIGPZCFNTPOD-KRWDZBQOSA-N |  | O |  |  |
| 188 | MOL004936 | QSIXTSXIETZHGN-UHFFFAOYSA-N |  |  |  | O |
| 189 | MOL004937 | VWQASRWQZBVNEI-QFOLPQNPSA-N |  |  |  | O |
| 190 | MOL004938 | NIABBGMPPWXWOJ-ARJRJXGUSA-N |  | O |  |  |
| 191 | MOL004939 | SPZXXUUDYMHBSG-UHFFFAOYSA-N | O |  |  |  |
| 192 | MOL004940 | HJBUYKZTEBZNSH-ONJCETCRSA-N |  | O |  |  |
| 193 | MOL004941 | FURUXTVZLHCCNA-CQSZACIVSA-N |  | O |  |  |
| 194 | MOL004942 | ADOQBZAVKYCFOI-HWKANZROSA-N | O |  |  |  |
| 195 | MOL004943 | XQWFHGOIUZFQPJ-NCLODGLDSA-N |  | O |  |  |
| 196 | MOL004944 | ZQXBPTKCIMXYMS-UHFFFAOYSA-N | O |  |  |  |
| 197 | MOL004945 | KYFBXCHUXFKMGQ-IBGZPJMESA-N | O |  |  |  |
| 198 | MOL004946 | POQLVOYRGNFGRM-UHFFFAOYSA-N | O |  |  |  |
| 199 | MOL004947 | TWBWSPDILHVKEV-VRLQRIPXSA-N |  |  | O |  |
| 200 | MOL004948 | CFWLRXJPRRCJTI-UHFFFAOYSA-N |  | O |  |  |
| 201 | MOL004949 | PGCKDCPTJAQQSQ-UHFFFAOYSA-N |  | O |  |  |
| 202 | MOL004950 | PHHAXWBLJNBVNS-UHFFFAOYSA-N |  |  | O |  |
| 203 | MOL004951 | YNWXJFQOCHMPCK-NCLODGLDSA-N |  | O |  |  |
| 204 | MOL004952 | NIZFPXZQERMCLE-KVFWHIKKSA-N |  |  | O |  |
| 205 | MOL004953 | FTVKHUHJWDMWIR-LGHIDRSRSA-N |  |  | O |  |
| 206 | MOL004954 | MLXUEMGNSDHQTJ-HSZRJFAPSA-N | O |  |  |  |
| 207 | MOL004955 | NCFVZZZXSIOSQB-CQRARSCRSA-N |  |  | O |  |
| 208 | MOL004956 | YAYJWUXAGVZHGX-MIUGBVLSSA-N |  | O |  |  |
| 209 | MOL004957 | LNIQZRIHAMVRJA-UHFFFAOYSA-N |  |  | O |  |
| 210 | MOL004958 | OVMFOVNOXASTPA-VYUBKLCTSA-N |  | O |  |  |
| 211 | MOL004959 | YKTZRMXYANFKQR-YCRPNKLZSA-N | O |  |  |  |
| 212 | MOL004960 | VVMZSXAUZNQUQA-UQENKSBZSA-N | O |  |  |  |
| 213 | MOL004961 | FMEHGPQTMOPUGM-UHFFFAOYSA-N |  |  |  | O |
| 214 | MOL004962 | KHABBSONZWLPEN-XIRSUAFNSA-N | O |  |  |  |
| 215 | MOL004963 | GSEPOEIKWTXTHS-WTMNEXLISA-N |  |  | O |  |
| 216 | MOL004964 | JUMSUVHHUVPSOY-TWGQIWQCSA-N | O |  |  |  |
| 217 | MOL004965 | FSHPJPOJLGCQOJ-GOSISDBHSA-N |  | O |  |  |
| 218 | MOL004966 | PPBISUGOQDBBEL-ZDUSSCGKSA-N |  |  | O |  |
| 219 | MOL004967 | AEXMKKGTQYQZCS-UHFFFAOYSA-N | O |  |  |  |
| 220 | MOL004968 | NVKZAILFHGUXNI-IHWYPQMZSA-N | O |  |  |  |
| 221 | MOL004969 | AXIUBBVSOWPLDA-UHFFFAOYSA-N | O |  |  |  |
| 222 | MOL004970 | LAIUFBWHERIJIH-MRVPVSSYSA-N | O |  |  |  |
| 223 | MOL004971 | VLJXXKKOSFGPHI-SSDOTTSWSA-N | O |  |  |  |
| 224 | MOL004972 | PFEOZHBOMNWTJB-UHFFFAOYSA-N | O |  |  |  |
| 225 | MOL004973 | AORMDLNPRGXHHL-UHFFFAOYSA-N | O |  |  |  |
| 226 | MOL004974 | SBQBKTSYEKPBJF-ZDUSSCGKSA-N |  | O |  |  |
| 227 | MOL004975 | OVAHJMNGYVAEDT-VGPVGYFWSA-N | O |  |  |  |
| 228 | MOL004976 | LPNBCGIVZXHHHO-UHFFFAOYSA-N | O |  |  |  |
| 229 | MOL004977 | WIEKYGJSRGBBTQ-AFMDSPMNSA-N |  |  | O |  |
| 230 | MOL004978 | ZZAIPFIGEGQNHP-AWEZNQCLSA-N |  | O |  |  |
| 231 | MOL004979 | SLKHLLNCFGPWAZ-CQSZACIVSA-N |  |  | O |  |
| 232 | MOL004980 | RNBLSJGPSGNSIN-UHFFFAOYSA-N |  |  | O |  |
| 233 | MOL004981 | IQHPDUUSMBMDGN-WEVVVXLNSA-N | O |  |  |  |
| 234 | MOL004982 | VOLMSPGWNYJHQQ-SCSAIBSYSA-N | O |  |  |  |
| 235 | MOL004983 | LGYCOYCCCKHXGC-UHFFFAOYSA-N |  |  |  | O |
| 236 | MOL004985 | FPAQLJHSZVFKES-FOCLMDBBSA-N | O |  |  |  |
| 237 | MOL004986 | TYDRPFWXPHBCDP-ZXGBLKHXSA-N |  |  | O |  |
| 238 | MOL004987 | JZFSMVXQUWRSIW-BTJIZOSBSA-N |  |  | O |  |
| 239 | MOL004988 | SVHCNENPWOPFOI-BVZFJXPGSA-N |  |  | O |  |
| 240 | MOL004989 | KBEFFXBSHFUQLT-QGZVFWFLSA-N |  |  | O |  |
| 241 | MOL004990 | FTOHMMMSWYNATM-UHFFFAOYSA-N | O |  |  |  |
| 242 | MOL004991 | DPIAJERHFDBLPT-UHFFFAOYSA-N | O |  |  |  |
| 243 | MOL004992 | BBCDTCKKROIGAB-UHFFFAOYSA-N | O |  |  |  |
| 244 | MOL004993 | XJUDJFVOICLOMY-GOSISDBHSA-N |  |  | O |  |
| 245 | MOL004994 | BJIUDNXPLSJWKE-OAHLLOKOSA-N | O |  |  |  |
| 246 | MOL004995 | JRVDUBFSQWHYRJ-INIZCTEOSA-N |  |  | O |  |
| 247 | MOL004996 | LQJBNNIYVWPHFW-VAWYXSNFSA-N | O |  |  |  |
| 248 | MOL004997 | DTMJARJUCFBMFR-SEMTUGJKSA-N |  |  | O |  |
| 249 | MOL004999 | HDHRTQZSBFUBMJ-UHFFFAOYSA-N | O |  |  |  |
| 250 | MOL005000 | WLPHLDLTTPUDSI-UHFFFAOYSA-N |  |  | O |  |
| 251 | MOL005001 | CCUZOQMNQONIMX-UHFFFAOYSA-N |  |  | O |  |
| 252 | MOL005002 | MPDGHEJMBKOTSU-PMTKVOBESA-N |  | O |  |  |
| 253 | MOL005003 | NYWUHXBEDBSRQB-UWJYYQICSA-N | O |  |  |  |
| 254 | MOL005004 | DKVBYQAVNNRVNN-UHFFFAOYSA-N |  | O |  |  |
| 255 | MOL005005 | XHCCZOWAHHBCAK-UHFFFAOYSA-N |  |  | O |  |
| 256 | MOL005006 | RDSJBMMQOCZNLR-GOSISDBHSA-N | O |  |  |  |
| 257 | MOL005007 | ZIDJFMZHWCAFPM-AWEZNQCLSA-N | O |  |  |  |
| 258 | MOL005008 | UFWHTSBKDGUFOX-OAHLLOKOSA-N |  |  |  | O |
| 259 | MOL005009 | DUWPGRAKHMEPCM-IZZDOVSWSA-N |  | O |  |  |
| 260 | MOL005010 | YQCBVKUBTQVHOT-UHFFFAOYSA-N | O |  |  |  |
| 261 | MOL005011 | IOXLCTZITMJUKD-PKTZIBPZSA-N | O |  |  |  |
| 262 | MOL005012 | CQXOSSCKEFRIBR-OAHLLOKOSA-N | O |  |  |  |
| 263 | MOL005013 | FCVHQYZUEPQNJU-JPQXAEDTSA-N | O |  |  |  |
| 264 | MOL005014 | BYQKEKUAWGGZTQ-JCEWGHKSSA-N |  |  | O |  |
| 265 | MOL005015 | GDAAEAXMNLVRCZ-SFHVURJKSA-N |  |  | O |  |
| 266 | MOL005016 | BYNYZQQDQIQLSO-UHFFFAOYSA-N | O |  |  |  |
| 267 | MOL005017 | FRXPSBUCIWPZMH-UHFFFAOYSA-N | O |  |  |  |
| 268 | MOL005018 | FGJUXFVUOCKRCY-QFIPXVFZSA-N | O |  |  |  |
| 269 | MOL005019 | NLTOTZSPOYWSSP-LJQANCHMSA-N |  |  | O |  |
| 270 | MOL005020 | DRUAOMUKHSJILC-UHFFFAOYSA-N | O |  |  |  |
| 271 | MOL005021 | NIQCNGHVCWTJSM-UHFFFAOYSA-N | O |  |  |  |
| 272 | MOL005812 | DFPMSGMNTNDNHN-ZPHOTFPESA-N |  |  | O |  |

Table S5 List of licorice compounds in TCMID and their existence in TM-MC and TCMSP.

| No | ID | INCHIKEY | Only TCMID | TCMID, TM-MC, and TCMSP | TCMID and TM-MC | TCMID and TCMSP |
| --- | --- | --- | --- | --- | --- | --- |
| 1 | 403 |  | O |  |  |  |
| 2 | 1935 | JPUKWEQWGBDDQB-ZVIPYZFUSA-N |  | O |  |  |
| 3 | 2455 |  | O |  |  |  |
| 4 | 4108 | DUWPGRAKHMEPCM-IZZDOVSWSA-N |  | O |  |  |
| 5 | 5404 |  | O |  |  |  |
| 6 | 6023 | DYPJOHFWCNIBKZ-RMKNXTFCSA-N | O |  |  |  |
| 7 | 6024 | CBGDCCSHOGQUSW-MDWZMJQESA-N |  | O |  |  |
| 8 | 6027 | PFYHYHZGDNWFIF-KVTDHHQDSA-N | O |  |  |  |
| 9 | 6306 | FSHPJPOJLGCQOJ-UHFFFAOYSA-N |  | O |  |  |
| 10 | 6401 | FMEHGPQTMOPUGM-UHFFFAOYSA-N |  |  |  | O |
| 11 | 6405 | ALOUNLDAKADEEB-UHFFFAOYSA-N | O |  |  |  |
| 12 | 7421 | DTWCSMODIRYEAA-YWQJNNKFSA-N | O |  |  |  |
| 13 | 7882 | HKQYGTCOTHHOMP-OPOMHRLSSA-N |  | O |  |  |
| 14 | 7884 | GWOKWCRSUJQOMD-QMCAAQAGSA-N | O |  |  |  |
| 15 | 8133 | JQNSUDIGIIGIOL-UHFFFAOYSA-N |  |  |  | O |
| 16 | 8134 | YQEPOQVRUDADPH-UHFFFAOYSA-N |  |  |  | O |
| 17 | 8135 | MEADLGUPYQNUNF-BIIKFXOESA-N |  | O |  |  |
| 18 | 8136 | UCKSAYIMWMIZQJ-QDEBKDIKSA-N |  | O |  |  |
| 19 | 8137 | HCBKENVWCDLQOA-UHFFFAOYSA-N |  | O |  |  |
| 20 | 8138 | YURHIASRSSMERJ-UHFFFAOYSA-N |  |  | O |  |
| 21 | 8139 | DKVBYQAVNNRVNN-UHFFFAOYSA-N |  | O |  |  |
| 22 | 8141 | UXTFKMCFQVSJLL-UHFFFAOYSA-N | O |  |  |  |
| 23 | 8142 | DRIPWQOGMJYOPU-UHFFFAOYSA-N | O |  |  |  |
| 24 | 8499 | SSHDNSCEQSPWIM-FVTWEACWSA-N |  | O |  |  |
| 25 | 8552 | RIWDYFGEQJAMKI-UHFFFAOYSA-N |  |  | O |  |
| 26 | 8580 | MXIZCSZWQVEQQV-MRIXOBAXSA-N | O |  |  |  |
| 27 | 8763 |  | O |  |  |  |
| 28 | 8794 | DPLWUTYEBRKBLI-UHFFFAOYSA-N |  |  | O |  |
| 29 | 8832 | NZYSZZDSYIBYLC-UHFFFAOYSA-N |  | O |  |  |
| 30 | 8833 | GLLUYNRFPAMGQR-WTKKLPLVSA-N | O |  |  |  |
| 31 | 8837 | FWWGXZYUURXJLK-UHFFFAOYSA-N |  | O |  |  |
| 32 | 8838 | LWESBHWAOZORCQ-UHFFFAOYSA-N |  | O |  |  |
| 33 | 8839 | TUUBGLDJKKCMRH-UHFFFAOYSA-N |  | O |  |  |
| 34 | 8840 | OBZHEBDUNPOCJG-ILYNEHRHSA-N |  |  | O |  |
| 35 | 8841 | JCGXIYQLRYPHDG-DVAIWGJFSA-N |  | O |  |  |
| 36 | 8842 | OTJMSWBNEUNNEW-UHFFFAOYSA-N |  |  | O |  |
| 37 | 8843 | JOQWUUJQWPZLAT-UHFFFAOYSA-N |  |  | O |  |
| 38 | 8844 | UFWHTSBKDGUFOX-UHFFFAOYSA-N |  |  |  | O |
| 39 | 8845 | LPLVUJXQOOQHMX-MOGLOQIBSA-N |  | O |  |  |
| 40 | 8846 | PFOWZHHJGPFJOO-AXWGKNCXSA-O | O |  |  |  |
| 41 | 8853 | DPLWUTYEBRKBLI-UHFFFAOYSA-N |  |  | O |  |
| 42 | 8854 | MKDSBDQLSLPNOQ-UHFFFAOYSA-N |  | O |  |  |
| 43 | 8855 | LKBVWZAHWLLJRO-IAPCRSCMSA-N | O |  |  |  |
| 44 | 8861 | GRMSSCUVELGNHC-UHFFFAOYSA-N |  | O |  |  |
| 45 | 8862 | ITOFWMRNIIFZKF-UTLUCORTSA-N | O |  |  |  |
| 46 | 9556 | HEHXVJXTDHVOBI-UHFFFAOYSA-N |  | O |  |  |
| 47 | 9557 | CJUFYKORDZSOLF-INIZCTEOSA-N |  | O |  |  |
| 48 | 9563 | IHFBPDAQLQOCBX-UHFFFAOYSA-N | O |  |  |  |
| 49 | 10148 | LAQLCZKPJGMFRM-UXMRNZNESA-N |  | O |  |  |
| 50 | 11441 | OJFZQSUOBVJAIP-UHFFFAOYSA-N |  | O |  |  |
| 51 | 11442 | RTUPRHIHXSAWDP-UHFFFAOYSA-N | O |  |  |  |
| 52 | 11487 | PGCKDCPTJAQQSQ-UHFFFAOYSA-N |  | O |  |  |
| 53 | 11488 | AJPXZTKPPINUKN-UHFFFAOYSA-N | O |  |  |  |
| 54 | 11501 | DXDRHHKMWQZJHT-BAQGIRSFSA-N |  | O |  |  |
| 55 | 11502 | YNWXJFQOCHMPCK-LXGDFETPSA-N |  | O |  |  |
| 56 | 11503 | OMAMGHBETNHQJC-KDURUIRLSA-N | O |  |  |  |
| 57 | 11573 | YAYJWUXAGVZHGX-MIUGBVLSSA-N |  | O |  |  |
| 58 | 11639 | OFVMWMCWOZJOOS-UHFFFAOYSA-N |  |  | O |  |
| 59 | 11640 | NWFNMRFBJUONKD-SIQHXTPISA-N | O |  |  |  |
| 60 | 11694 | OVMFOVNOXASTPA-ICQLDYLPSA-N |  | O |  |  |
| 61 | 11743 | OVLUQDOJWGTPHA-UHFFFAOYSA-N |  | O |  |  |
| 62 | 11744 | IGGMWDYWFSLMOW-SVBPBHIXSA-N | O |  |  |  |
| 63 | 12146 |  | O |  |  |  |
| 64 | 12147 |  | O |  |  |  |
| 65 | 12758 | KRWXHCWICDLYOY-UHFFFAOYSA-N |  | O |  |  |
| 66 | 12759 | WJSPTHUPUYBNNI-IZZNHLLZSA-N | O |  |  |  |
| 67 | 12760 | KAZSKMJFUPEHHW-DHZHZOJOSA-N |  | O |  |  |
| 68 | 12765 | CNPMAFLUEHEXRE-UHFFFAOYSA-N |  | O |  |  |
| 69 | 12771 | GAUFLNQQCSXBPK-UHFFFAOYSA-N |  | O |  |  |
| 70 | 12772 | KIZPADOTOCPASX-UHFFFAOYSA-N |  | O |  |  |
| 71 | 12774 | CIJATEIGJFIOPE-OUOXKOSGSA-N |  |  | O |  |
| 72 | 12776 | MOBCUWLJOZHPQL-UHFFFAOYSA-N |  | O |  |  |
| 73 | 12777 | HJFOOTRGDAPZMV-SMVKYPPISA-N |  |  | O |  |
| 74 | 12778 | BCNKILSUUHWRTG-JVHDORQOSA-N |  | O |  |  |
| 75 | 12779 | WPDHECQXVOACTR-KSHPUXFISA-N |  | O |  |  |
| 76 | 12780 | PLPHOQSHVPRIJE-HOVVJNQOSA-N |  |  | O |  |
| 77 | 12781 | ACCYCJOHUMRMMV-NHJGESHHSA-N |  |  | O |  |
| 78 | 12782 |  |  | O |  |  |
| 79 | 12783 | WBQVRPYEEYUEBQ-OJVDLISWSA-N |  | O |  |  |
| 80 | 12784 |  |  | O |  |  |
| 81 | 12785 | SLWCVFLNZDOMEZ-SVTBZOAZSA-N |  | O |  |  |
| 82 | 12786 | IPBGQSWRSQPGCF-LIOOBZAHSA-N |  | O |  |  |
| 83 | 12787 | HWJXKLRHGKKFBY-SOFGYWHQSA-N |  | O |  |  |
| 84 | 12788 | GGWMNTNDTRKETA-UHFFFAOYSA-N |  | O |  |  |
| 85 | 12789 | AXLJPECJCPNXRX-FQEVSTJZSA-N |  |  | O |  |
| 86 | 12902 | FURUXTVZLHCCNA-CQSZACIVSA-N |  | O |  |  |
| 87 | 12905 |  | O |  |  |  |
| 88 | 12906 | UCUBMAVPVJYHIR-BPKFCZSYSA-N |  |  | O |  |
| 89 | 12907 | DEMKZLAVQYISIA-UZQFATADSA-N |  | O |  |  |
| 90 | 13100 | YGCCASGFIOIXIN-UHFFFAOYSA-M |  | O |  |  |
| 91 | 13934 | SBQBKTSYEKPBJF-UHFFFAOYSA-N |  | O |  |  |
| 92 | 13935 | DGLBFMRIIDZOJI-QKYBYQKWSA-N | O |  |  |  |
| 93 | 14178 | DHJJPSKLKCWPDR-DCEKSMFGSA-N | O |  |  |  |
| 94 | 14458 | ZZAIPFIGEGQNHP-AWEZNQCLSA-N |  | O |  |  |
| 95 | 14466 | RMIVRCBSQPCSCQ-BDANYOJNSA-N |  |  | O |  |
| 96 | 14467 | AIJULSRZWUXGPQ-UHFFFAOYSA-N | O |  |  |  |
| 97 | 14493 | OLFMGIZTWHFEJC-LTJBUQOPSA-N | O |  |  |  |
| 98 | 14494 | ODKFBQCYKKPNLJ-UHFFFAOYSA-N | O |  |  |  |
| 99 | 14499 |  | O |  |  |  |
| 100 | 14500 | XUYKSQAOCYIBAB-YRCFHROASA-N | O |  |  |  |
| 101 | 14505 | QZLKGEWVHZXRAM-ISJQQBSNSA-N | O |  |  |  |
| 102 | 14546 | PKIXXJPMNDDDOS-MVQNEBOGSA-N | O |  |  |  |
| 103 | 14766 | KRYURACLPUIPBO-UHFFFAOYSA-N | O |  |  |  |
| 104 | 14767 | JKJVBHYKKRDSPP-UHFFFAOYSA-N | O |  |  |  |
| 105 | 15257 | UIDGLYUNOUKLBM-LQBNSVKOSA-N |  | O |  |  |
| 106 | 15280 | QENVUHCAYXAROT-MCVKWTAESA-N | O |  |  |  |
| 107 | 15393 | OKNPZRJNRSGKME-ZKRIFWAISA-N | O |  |  |  |
| 108 | 15406 | XQWFHGOIUZFQPJ-XETXNTDKSA-N |  | O |  |  |
| 109 | 15407 | ZYTMANIQRDEHIO-UTLUCORTSA-N | O |  |  |  |
| 110 | 15421 | HJBUYKZTEBZNSH-BDHDDQLPSA-N |  | O |  |  |
| 111 | 15425 | DUQRVHURUWMFMN-CHHOWFRJSA-N | O |  |  |  |
| 112 | 15462 | XHGNOEWBXVPYDM-UHFFFAOYSA-N |  |  |  | O |
| 113 | 15467 | NUIWIFSXKJCQOO-BPUDJEHESA-N | O |  |  |  |
| 114 | 15516 | RTATXGUCZHCSNG-SRZXWKELSA-N |  | O |  |  |
| 115 | 16108 | DSCFFEYYQKSRSV-CEDNOLFGSA-N | O |  |  |  |
| 116 | 17035 | UUJBHSNXZMGYBT-ZDUSSCGKSA-N |  |  |  | O |
| 117 | 17037 | YTDNHMHONBWCBV-UHFFFAOYSA-N | O |  |  |  |
| 118 | 19072 | ZMDOPLQSOGZPJN-UHFFFAOYSA-N |  | O |  |  |
| 119 | 19075 | RUXOGPGQKCWGOH-BGEHYGTQSA-N | O |  |  |  |
| 120 | 19448 | NIABBGMPPWXWOJ-UHFFFAOYSA-N |  | O |  |  |
| 121 | 19869 | SFQIGPZCFNTPOD-UHFFFAOYSA-N |  | O |  |  |
| 122 | 19967 | KZJWDPNRJALLNS-VJSFXXLFSA-N |  | O |  |  |
| 123 | 21053 | ZBIKAJHAGKBFKR-UHFFFAOYSA-N |  |  |  | O |
| 124 | 21054 | ZXLDQJLIBNPEFJ-MRVPVSSYSA-N | O |  |  |  |
| 125 | 21059 | LGYCOYCCCKHXGC-UHFFFAOYSA-N |  |  |  | O |
| 126 | 21061 | MGSZZQQRTPWMEI-UHFFFAOYSA-N | O |  |  |  |
| 127 | 21578 | FIGVVZUWCLSUEI-UHFFFAOYSA-N | O |  |  |  |
| 128 | 21678 | VDYSHUXENHRSOO-XBXARRHUSA-N |  |  | O |  |
| 129 | 22179 | ORHBXUUXSCNDEV-RALIUCGRSA-N |  |  | O |  |
| 130 | 22222 | QSIXTSXIETZHGN-UHFFFAOYSA-N |  |  |  | O |
| 131 | 22223 | CQVDOHIEYGNTQX-UHFFFAOYSA-N |  |  | O |  |
| 132 | 22224 | VWQASRWQZBVNEI-KLBPJQLPSA-N |  |  |  | O |
| 133 | 22225 | WOMWVGHYSNATOB-UHFFFAOYSA-N |  | O |  |  |
| 134 | 22226 | VCKYLOIMXUHPDA-UHFFFAOYSA-N |  |  |  | O |
| 135 | 22227 | SJQLKQKISIAYNF-KESOWIAMSA-N |  |  | O |  |
| 136 | 22228 | AJQIRMNRIITJJA-UCUUBXCKSA-N |  | O |  |  |
| 137 | 22229 | BNTYXDBRBRYHOP-UHFFFAOYSA-N |  |  | O |  |
| 138 | 22230 | IKHVNSQXSIEHOC-UHFFFAOYSA-N | O |  |  |  |
| 139 | 22449 | YYYCJNDALLBNEG-YQCIQBACSA-N | O |  |  |  |
| 140 | 23092 | MPDGHEJMBKOTSU-YKLVYJNSSA-N |  | O |  |  |
| 141 | 23134 | ODBRNZZJSYPIDI-VJXVFPJBSA-M | O |  |  |  |
| 142 | 23139 | PCMORTLOPMLEFB-ONEGZZNKSA-M |  |  | O |  |
| 143 | 23166 | MPDGHEJMBKOTSU-WWCMNKNLSA-N |  | O |  |  |
| 144 | 23172 | ILRKKHJEINIICQ-UIAZACTHSA-O |  | O |  |  |
| 145 | 23251 | LPLVUJXQOOQHMX-YFINQFFOSA-N |  | O |  |  |
| 146 | 23259 | ILRKKHJEINIICQ-UFTZEXNXSA-N |  |  | O |  |
| 147 | 23291 | MPDGHEJMBKOTSU-WFJWTYAKSA-N |  | O |  |  |
| 148 | 23696 | KSEBMYQBYZTDHS-HWKANZROSA-M |  |  | O |  |
| 149 | 24040 | MGJLSBDCWOSMHL-YSZROVPYSA-N |  | O |  |  |
| 150 | 24351 |  | O |  |  |  |
| 151 | 24907 |  | O |  |  |  |
| 152 | 24912 |  | O |  |  |  |
| 153 | 25888 | CBGDCCSHOGQUSW-MDWZMJQESA-N |  | O |  |  |
| 154 | 27722 | OVUOUFPIPZJGME-JQDIJSAQSA-N | O |  |  |  |
| 155 | 29509 | KZJWDPNRJALLNS-JQEGLAGPSA-N |  | O |  |  |
| 156 | 30901 | BEKYZOSRHNMGCR-ALSKWLPDSA-N | O |  |  |  |
| 157 | 31195 |  | O |  |  |  |
| 158 | 31429 | HJGURBGBPIKRER-UHFFFAOYSA-N |  | O |  |  |
| 159 | 31430 | JNDPLDZUOFZXIG-UHFFFAOYSA-N |  | O |  |  |
| 160 | 31431 | HJFOOTRGDAPZMV-IRUBZKONSA-N |  |  | O |  |
| 161 | 31433 | NVPPTQRKNPFNKS-HMZVGEFBSA-N | O |  |  |  |
| 162 | 31434 | GUQRPJIGNVZDMJ-NBXKVVTISA-N | O |  |  |  |
| 163 | 31435 |  | O |  |  |  |
| 164 | 31436 | WBQVRPYEEYUEBQ-QRSKSGCISA-N |  | O |  |  |
| 165 | 31437 |  | O |  |  |  |
| 166 | 31438 | SLWCVFLNZDOMEZ-PDZCEQESSA-N |  | O |  |  |
| 167 | 31439 | IPBGQSWRSQPGCF-PIOCFCGTSA-N |  | O |  |  |
| 168 | 31440 | XCUCMLUTCAKSOZ-FIRIVFDPSA-N | O |  |  |  |
| 169 | 31453 |  | O |  |  |  |
| 170 | 31455 | NGWKGSCSHDHHAJ-YYDBBHKUSA-N |  | O |  |  |
| 171 | 31625 | ZRFXBWYDLDSWMT-ZRADNITFSA-N | O |  |  |  |
| 172 | 32116 | FFKCDLGZUKVOSI-DFWSKRRBSA-N | O |  |  |  |

Table S6 List of ginseng compounds in TM-MC and their existence in TCMSP and TCMID.

| No | ID | INCHIKEY | Only TM-MC | TM-MC, TCMSP, and TCMID | TM-MC and TCMSP | TM-MC and TCMID |
| --- | --- | --- | --- | --- | --- | --- |
| 1 | 107 | WBYWAXJHAXSJNI-VOTSOKGWSA-N | O |  |  |  |
| 2 | 109 | KSEBMYQBYZTDHS-HWKANZROSA-N | O |  |  |  |
| 3 | 111 | NGSWKAQJJWESNS-ZZXKWVIFSA-N | O |  |  |  |
| 4 | 113 | QAIPRVGONGVQAS-DUXPYHPUSA-N | O |  |  |  |
| 5 | 120 | LUKBXSAWLPMMSZ-OWOJBTEDSA-N | O |  |  |  |
| 6 | 135 | IKGXIBQEEMLURG-NVPNHPEKSA-N | O |  |  |  |
| 7 | 136 | IYRMWMYZSQPJKC-UHFFFAOYSA-N |  | O |  |  |
| 8 | 154 | FTVWIRXFELQLPI-ZDUSSCGKSA-N | O |  |  |  |
| 9 | 155 | AIONOLUJZLIMTK-AWEZNQCLSA-N | O |  |  |  |
| 10 | 156 | PFTAWBLQPZVEMU-DZGCQCFKSA-N | O |  |  |  |
| 11 | 171 | YQUVCSBJEUQKSH-UHFFFAOYSA-N | O |  |  |  |
| 12 | 172 | FJKROLUGYXJWQN-UHFFFAOYSA-N | O |  |  |  |
| 13 | 174 | CWVRJTMFETXNAD-JUHZACGLSA-N | O |  |  |  |
| 14 | 193 | ONIBWKKTOPOVIA-BYPYZUCNSA-N | O |  |  |  |
| 15 | 232 | OIRDTQYFTABQOQ-KQYNXXCUSA-N |  | O |  |  |
| 16 | 305 | GFFGJBXGBJISGV-UHFFFAOYSA-N |  |  |  | O |
| 17 | 309 | NYHBQMYGNKIUIF-UUOKFMHZSA-N |  |  |  | O |
| 18 | 310 | RWQNBRDOKXIBIV-UHFFFAOYSA-N | O |  |  |  |
| 19 | 312 | ISAKRJDGNUQOIC-UHFFFAOYSA-N | O |  |  |  |
| 20 | 314 | DRTQHJPVMGBUCF-XVFCMESISA-N |  |  |  | O |
| 21 | 316 | UHDGCWIWMRVCDJ-XVFCMESISA-N | O |  |  |  |
| 22 | 320 | UGQMRVRMYYASKQ-KQYNXXCUSA-N | O |  |  |  |
| 23 | 337 | SECPZKHBENQXJG-FPLPWBNLSA-N |  | O |  |  |
| 24 | 338 | IPCSVZSSVZVIGE-UHFFFAOYSA-N |  | O |  |  |
| 25 | 339 | OYHQOLUKZRVURQ-HZJYTTRNSA-N |  |  | O |  |
| 26 | 340 | ZQPPMHVWECSIRJ-KTKRTIGZSA-N | O |  |  |  |
| 27 | 341 | QIQXTHQIDYTFRH-UHFFFAOYSA-N | O |  |  |  |
| 28 | 375 | KZJWDPNRJALLNS-VJSFXXLFSA-N |  | O |  |  |
| 29 | 376 | NPJICTMALKLTFW-OFUAXYCQSA-N |  | O |  |  |
| 30 | 454 | NUJGJRNETVAIRJ-UHFFFAOYSA-N |  |  |  | O |
| 31 | 597 | OPTASPLRGRRNAP-UHFFFAOYSA-N | O |  |  |  |
| 32 | 892 | CDAISMWEOUEBRE-UHFFFAOYSA-N | O |  |  |  |
| 33 | 957 | KBPLFHHGFOOTCA-UHFFFAOYSA-N | O |  |  |  |
| 34 | 1049 | JUJWROOIHBZHMG-UHFFFAOYSA-N | O |  |  |  |
| 35 | 1132 | YOVSPTNQHMDJAG-QLFBSQMISA-N |  | O |  |  |
| 36 | 1183 | MWOOGOJBHIARFG-UHFFFAOYSA-N | O |  |  |  |
| 37 | 1255 | WQZGKKKJIJFFOK-DVKNGEFBSA-N |  | O |  |  |
| 38 | 1281 | GRWFGVWFFZKLTI-UHFFFAOYSA-N | O |  |  |  |
| 39 | 1344 | NRJSRRMTRWBSEY-MEFSGEJESA-N | O |  |  |  |
| 40 | 1345 | IZNXDDYAYWSDPM-PCFULCJUSA-N | O |  |  |  |
| 41 | 1346 | DGBBUGWAMAPGQH-DXSBKGMBSA-N | O |  |  |  |
| 42 | 1347 | YIZGCLJAZVEWSI-WDLLAQNMSA-N | O |  |  |  |
| 43 | 1348 | QOGHAXHWJGZEST-XEUUVMKCSA-N | O |  |  |  |
| 44 | 1349 | QORFORDBLZAYLJ-PUQYTEIESA-N | O |  |  |  |
| 45 | 1466 | BSAIUMLZVGUGKX-BQYQJAHWSA-N | O |  |  |  |
| 46 | 1545 | DFPMSGMNTNDNHN-ZPHOTFPESA-N | O |  |  |  |
| 47 | 1933 | YMBFCQPIMVLNIU-SOUVJXGZSA-N | O |  |  |  |
| 48 | 2081 | WTTJVINHCBCLGX-NQLNTKRDSA-N |  |  | O |  |
| 49 | 2175 | COGPRPSWSKLKTF-UHFFFAOYSA-N | O |  |  |  |
| 50 | 2183 | DUYRYUZIBGFLDD-UHFFFAOYSA-N | O |  |  |  |
| 51 | 2937 | ADDQXUZECUAIGS-UHFFFAOYSA-N | O |  |  |  |
| 52 | 2969 | GHVNFZFCNZKVNT-UHFFFAOYSA-N | O |  |  |  |
| 53 | 3040 | IVBZYUKCNLJUDA-UHFFFAOYSA-N | O |  |  |  |
| 54 | 3327 | CRDAMVZIKSXKFV-UHFFFAOYSA-N | O |  |  |  |
| 55 | 3395 | VOLMSPGWNYJHQQ-UHFFFAOYSA-N | O |  |  |  |
| 56 | 3469 | WXTMDXOMEHJXQO-UHFFFAOYSA-N | O |  |  |  |
| 57 | 3515 | FWKQNCXZGNBPFD-UHFFFAOYSA-N | O |  |  |  |
| 58 | 3563 | TUMCLUKPDAUYFA-GUHGQFERSA-N | O |  |  |  |
| 59 | 3578 | NODILNFGTFIURN-USYOXQFSSA-N |  | O |  |  |
| 60 | 3579 | XUBSZCIZVSPSMQ-HTVDCONZSA-N |  | O |  |  |
| 61 | 3586 | TUMCLUKPDAUYFA-BYZJAHJESA-N | O |  |  |  |
| 62 | 3615 | FBFMBWCLBGQEBU-RXMALORBSA-N |  |  |  | O |
| 63 | 3674 | PSOUXXNNRFNUAY-UCWDMQAYSA-N | O |  |  |  |
| 64 | 3750 | KZJWDPNRJALLNS-FBZNIEFRSA-N |  | O |  |  |
| 65 | 3893 | POULHZVOKOAJMA-UHFFFAOYSA-N | O |  |  |  |
| 66 | 4007 | NTOPKICPEQUPPH-UHFFFAOYSA-N | O |  |  |  |
| 67 | 4528 | GGHMUJBZYLPWFD-CUZKYEQNSA-N |  |  |  | O |
| 68 | 5602 | BQSLMQNYHVFRDT-UHFFFAOYSA-N |  |  |  | O |
| 69 | 6184 | JARKCYVAAOWBJS-UHFFFAOYSA-N | O |  |  |  |
| 70 | 6326 | HSFWRNGVRCDJHI-UHFFFAOYSA-N | O |  |  |  |
| 71 | 6623 | IISBACLAFKSPIT-UHFFFAOYSA-N | O |  |  |  |
| 72 | 6949 | GIBQERSGRNPMEH-UHFFFAOYSA-N | O |  |  |  |
| 73 | 7002 | QPUYECUOLPXSFR-UHFFFAOYSA-N | O |  |  |  |
| 74 | 7253 | CNHDIAIOKMXOLK-UHFFFAOYSA-N | O |  |  |  |
| 75 | 7302 | YEJRWHAVMIAJKC-UHFFFAOYSA-N | O |  |  |  |
| 76 | 7361 | XPFVYQJUAUNWIW-UHFFFAOYSA-N | O |  |  |  |
| 77 | 7641 | SAOKZLXYCUGLFA-UHFFFAOYSA-N | O |  |  |  |
| 78 | 7720 | YIWUKEYIRIRTPP-UHFFFAOYSA-N | O |  |  |  |
| 79 | 7778 | DZNVIZQPWLDQHI-UHFFFAOYSA-N | O |  |  |  |
| 80 | 7883 | XPGWKKLDFXNBPJ-QTPLKFIXSA-N | O |  |  |  |
| 81 | 7976 | CAWHJQAVHZEVTJ-UHFFFAOYSA-N | O |  |  |  |
| 82 | 8130 | FXHGMKSSBGDXIY-UHFFFAOYSA-N | O |  |  |  |
| 83 | 8182 | SNRUBQQJIBEYMU-UHFFFAOYSA-N |  |  |  | O |
| 84 | 8215 | UKMSUNONTOPOIO-UHFFFAOYSA-N | O |  |  |  |
| 85 | 8221 | GLDOVTGHNKAZLK-UHFFFAOYSA-N | O |  |  |  |
| 86 | 8222 | CBFCDTFDPHXCNY-UHFFFAOYSA-N |  |  |  | O |
| 87 | 8247 | DTGKSKDOIYIVQL-QXFUBDJGSA-N | O |  |  |  |
| 88 | 8352 | FUCYIEXQVQJBKY-ZFWWWQNUSA-N |  |  |  | O |
| 89 | 8369 | XPCTZQVDEJYUGT-UHFFFAOYSA-N | O |  |  |  |
| 90 | 8507 | CRPUJAZIXJMDBK-UHFFFAOYSA-N | O |  |  |  |
| 91 | 8516 | VLXDPFLIRFYIME-BTFPBAQTSA-N | O |  |  |  |
| 92 | 8892 | FUZZWVXGSFPDMH-UHFFFAOYSA-N | O |  |  |  |
| 93 | 8908 | AOGQPLXWSUTHQB-UHFFFAOYSA-N | O |  |  |  |
| 94 | 9320 | FRMCCTDTYSRUBE-HYFYGGESSA-N | O |  |  |  |
| 95 | 9812 | WMOPMQRJLLIEJV-IUODEOHRSA-N | O |  |  |  |
| 96 | 10407 | JSNRRGGBADWTMC-NTCAYCPXSA-N |  |  |  | O |
| 97 | 10432 | OZQAPQSEYFAMCY-QLFBSQMISA-N |  |  |  | O |
| 98 | 10465 | KEMQGTRYUADPNZ-UHFFFAOYSA-N | O |  |  |  |
| 99 | 10494 | MIJYXULNPSFWEK-GTOFXWBISA-N |  |  | O |  |
| 100 | 10586 | RGZSQWQPBWRIAQ-LSDHHAIUSA-N | O |  |  |  |
| 101 | 10624 | HKQYGTCOTHHOMP-UHFFFAOYSA-N | O |  |  |  |
| 102 | 10690 | RGHNJXZEOKUKBD-SQOUGZDYSA-N | O |  |  |  |
| 103 | 10797 | VQKFNUFAXTZWDK-UHFFFAOYSA-N | O |  |  |  |
| 104 | 11005 | TUNFSRHWOTWDNC-UHFFFAOYSA-N | O |  |  |  |
| 105 | 11006 | DCAYPVUWAIABOU-UHFFFAOYSA-N |  |  |  | O |
| 106 | 11071 | JBGYSAVRIDZNKA-HIDBURGXSA-N |  |  |  | O |
| 107 | 11080 | VEHGXAYGFVYFPO-GJXAEJTASA-N | O |  |  |  |
| 108 | 11084 | NFZYDZXHKFHPGA-WLUMXSMGSA-N |  | O |  |  |
| 109 | 11200 | QNOJYUZTGHGWKF-SPURBKRKSA-N | O |  |  |  |
| 110 | 11420 | HMSWAIKSFDFLKN-UHFFFAOYSA-N | O |  |  |  |
| 111 | 11439 | KZNICNPSHKQLFF-UHFFFAOYSA-N | O |  |  |  |
| 112 | 11635 | RZJRJXONCZWCBN-UHFFFAOYSA-N | O |  |  |  |
| 113 | 12025 | HNJBEVLQSNELDL-UHFFFAOYSA-N | O |  |  |  |
| 114 | 12097 | OUDFNZMQXZILJD-UHFFFAOYSA-N | O |  |  |  |
| 115 | 12170 | CKOYRRWBOKMNRG-UHFFFAOYSA-N | O |  |  |  |
| 116 | 12255 | JPUKWEQWGBDDQB-DTGCRPNFSA-N |  |  |  | O |
| 117 | 12388 | IIYFAKIEWZDVMP-UHFFFAOYSA-N |  |  |  | O |
| 118 | 12391 | YCOZIPAWZNQLMR-UHFFFAOYSA-N |  | O |  |  |
| 119 | 12398 | NDJKXXJCMXVBJW-UHFFFAOYSA-N |  |  |  | O |
| 120 | 12401 | LQERIDTXQFOHKA-UHFFFAOYSA-N |  |  |  | O |
| 121 | 12403 | FNAZRRHPUDJQCJ-UHFFFAOYSA-N | O |  |  |  |
| 122 | 12405 | HOWGUJZVBDQJKV-UHFFFAOYSA-N | O |  |  |  |
| 123 | 12406 | YKNWIILGEFFOPE-UHFFFAOYSA-N | O |  |  |  |
| 124 | 12479 | HMPXCABYJUUDEV-STYIPFKDSA-N | O |  |  |  |
| 125 | 12482 | IJQCDXJUXSOKKW-HNNUEMMQSA-N | O |  |  |  |
| 126 | 12483 | IFRFMSPXPRNUSG-BMTZGSSCSA-N | O |  |  |  |
| 127 | 12523 | GGYKPYDKXLHNTI-UHFFFAOYSA-N | O |  |  |  |
| 128 | 12534 | FIGVVZUWCLSUEI-UHFFFAOYSA-N | O |  |  |  |
| 129 | 12592 | POOSGDOYLQNASK-UHFFFAOYSA-N | O |  |  |  |
| 130 | 12687 | KDCDEJOGNTYMEQ-UHFFFAOYSA-N | O |  |  |  |
| 131 | 12756 | JBFHTYHTHYHCDJ-UHFFFAOYSA-N | O |  |  |  |
| 132 | 12860 | VUMCUSHVMYIRMB-UHFFFAOYSA-N | O |  |  |  |
| 133 | 12974 | UOFHLCPZXZURFL-GPDZXBECSA-N |  | O |  |  |
| 134 | 13020 | YBQOLFRVPRVASB-CYLVDRAXSA-N | O |  |  |  |
| 135 | 13243 | FLIACVVOZYBSBS-UHFFFAOYSA-N |  | O |  |  |
| 136 | 13558 | KSDSYIXRWHRPMN-RGHIGTIISA-N | O |  |  |  |
| 137 | 13849 | WQEPLUUGTLDZJY-UHFFFAOYSA-N |  | O |  |  |
| 138 | 14079 | IGJQUJNPMOYEJY-UHFFFAOYSA-N | O |  |  |  |
| 139 | 14104 | LSQXNMXDFRRDSJ-UHFFFAOYSA-N | O |  |  |  |
| 140 | 14116 | ZEQZCZRDJPTCHI-JCHYFPDLSA-N |  |  |  | O |
| 141 | 14266 | ZSBWUNDRDHVNJL-UHFFFAOYSA-N | O |  |  |  |
| 142 | 14275 | DHLUJPLHLZJUBW-UHFFFAOYSA-N | O |  |  |  |
| 143 | 14296 | FINHMKGKINIASC-UHFFFAOYSA-N | O |  |  |  |
| 144 | 14542 | XOJVVFBFDXDTEG-UHFFFAOYSA-N | O |  |  |  |
| 145 | 14594 | ZFISZYZPHSICQI-VAIHSBBOSA-N | O |  |  |  |
| 146 | 14728 | YDQIRODFTJGGMP-CFXKPOTGSA-N | O |  |  |  |
| 147 | 14985 | GVJHHUAWPYXKBD-IEOSBIPESA-N | O |  |  |  |
| 148 | 15094 | WRHGORWNJGOVQY-ZNMIVQPWSA-N |  |  |  | O |
| 149 | 15248 | SNHCPECPLQRJNL-GWNBYJSOSA-N |  |  |  | O |
| 150 | 15265 | RJWUMFHQJJBBOD-UHFFFAOYSA-N | O |  |  |  |
| 151 | 15267 | BANXPJUEBPWEOT-UHFFFAOYSA-N | O |  |  |  |
| 152 | 15285 | QDUJKDRUFBJYSQ-HNNXBMFYSA-N | O |  |  |  |
| 153 | 16319 | ZALHPSXXQIPKTQ-UHFFFAOYSA-N | O |  |  |  |
| 154 | 16320 | WXQGPFZDVCRBME-QEJZJMRPSA-N | O |  |  |  |
| 155 | 16837 | AYXPYQRXGNDJFU-OMDCQRFSSA-N | O |  |  |  |
| 156 | 16937 | SNPBSTALMDUCRQ-GYDRUNPJSA-N | O |  |  |  |
| 157 | 17139 | CXENHBSYCFFKJS-VDQVFBMKSA-N |  | O |  |  |
| 158 | 17140 | CXENHBSYCFFKJS-LOQWIJHWSA-N |  | O |  |  |
| 159 | 17188 | WHXUZXDWQKUIJL-UHFFFAOYSA-N |  |  |  | O |
| 160 | 17488 | FMXKKHBXBBAQBC-UHFFFAOYSA-N | O |  |  |  |
| 161 | 17581 | LHYHMMRYTDARSZ-LJISPDSOSA-N |  |  | O |  |
| 162 | 17595 | LHYHMMRYTDARSZ-AJNGGQMLSA-N |  |  | O |  |
| 163 | 18827 | VSMOENVRRABVKN-UHFFFAOYSA-N | O |  |  |  |
| 164 | 19602 | YVBAUDVGOFCUSG-UHFFFAOYSA-N | O |  |  |  |
| 165 | 19773 | YFHFHLSMISYUAQ-UHFFFAOYSA-N | O |  |  |  |
| 166 | 20335 | LHYHMMRYTDARSZ-BYNSBNAKSA-N |  |  | O |  |
| 167 | 21585 | ZZTHVSDJFCDCGT-DTBLUQRWSA-N | O |  |  |  |
| 168 | 22311 | XMGQYMWWDOXHJM-UHFFFAOYSA-N | O |  |  |  |
| 169 | 23741 | DSZTYVZOIUIIGA-UHFFFAOYSA-N | O |  |  |  |
| 170 | 24585 | ZYTMANIQRDEHIO-UHFFFAOYSA-N | O |  |  |  |
| 171 | 25913 | PJLHTVIBELQURV-UHFFFAOYSA-N | O |  |  |  |
| 172 | 28129 | QJVXKWHHAMZTBY-GCPOEHJPSA-N | O |  |  |  |
| 173 | 28812 | IYFUQKGDILUVJG-UHFFFAOYSA-N | O |  |  |  |
| 174 | 28813 | UVIVWIFUPKGWGF-UHFFFAOYSA-N | O |  |  |  |
| 175 | 30167 | WTTJVINHCBCLGX-ZDVGBALWSA-N |  |  | O |  |
| 176 | 30175 | CRDAMVZIKSXKFV-FBXUGWQNSA-N | O |  |  |  |
| 177 | 30424 | ZXVDYQWETIMVLL-FPLPWBNLSA-N | O |  |  |  |
| 178 | 31253 | UAHWPYUMFXYFJY-UHFFFAOYSA-N | O |  |  |  |
| 179 | 31289 | GYHFUZHODSMOHU-UHFFFAOYSA-N | O |  |  |  |
| 180 | 31404 | NLZUEZXRPGMBCV-UHFFFAOYSA-N |  |  |  | O |
| 181 | 33037 | DSLZVSRJTYRBFB-LLEIAEIESA-N | O |  |  |  |
| 182 | 35768 | FONXOARHSFUBAN-UHFFFAOYSA-N | O |  |  |  |
| 183 | 35781 | HAJTYGKLQDXTPO-ODKZNWOASA-N | O |  |  |  |
| 184 | 36655 | XXFSYINDUHLBIL-UHFFFAOYSA-N | O |  |  |  |
| 185 | 37611 | FAMPSKZZVDUYOS-HRGUGZIWSA-N |  |  |  | O |
| 186 | 37767 | UZIOUZHBUYLDHW-XUBRWZAZSA-N |  | O |  |  |
| 187 | 37775 | LTEPQNJXCFBNEO-FBWVZUCNSA-N | O |  |  |  |
| 188 | 37776 | LTEPQNJXCFBNEO-BIYWFNTMSA-N | O |  |  |  |
| 189 | 37777 | ZLZYKWSVPJGARJ-QEUZPZNGSA-N | O |  |  |  |
| 190 | 37778 | ZLZYKWSVPJGARJ-WTUZFMCISA-N | O |  |  |  |
| 191 | 37779 | SLPPUMWTJMNBCW-SZHHNJADSA-N | O |  |  |  |
| 192 | 37780 | PEOKIFXHILHWMQ-MZFDXQHNSA-N | O |  |  |  |
| 193 | 38578 | WUUDXONYKRKOSM-PHYASTDQSA-N | O |  |  |  |
| 194 | 38835 | AYMHVZBATCNDEX-YZSKAGILSA-N | O |  |  |  |
| 195 | 38860 | SSPJYWUVFLPKSH-HRUAZROZSA-N | O |  |  |  |
| 196 | 38862 | SSPJYWUVFLPKSH-USRQKLRVSA-N | O |  |  |  |
| 197 | 38903 | YIYRCZFIJNGYOG-QINBLQPGSA-N | O |  |  |  |
| 198 | 39012 | ZSTVHYZMXWWSOS-GDWXKANVSA-N | O |  |  |  |
| 199 | 39013 | YWMMUFIKXYPKRA-ODYFUNJHSA-N | O |  |  |  |
| 200 | 39251 | PFDULEUHGVVQHK-QHEGXUBCSA-N | O |  |  |  |
| 201 | 39252 | BVVQQTCEEXHFAK-JWEHFGOZSA-N | O |  |  |  |
| 202 | 39255 | BNULWEWUUICHNI-QHEGXUBCSA-N | O |  |  |  |
| 203 | 39399 | AGBCLJAHARWNLA-RPNKVCLTSA-N |  | O |  |  |
| 204 | 39401 | CMDWTCDYUNAUNH-JDCZMZFSSA-N | O |  |  |  |
| 205 | 39716 | DEXMKKYRTKSOCW-UHFFFAOYSA-N | O |  |  |  |
| 206 | 40115 | OZNHATCGPKOFBH-UHFFFAOYSA-N | O |  |  |  |
| 207 | 40229 | ZTQSADJAYQOCDD-HUGMCNGHSA-N | O |  |  |  |
| 208 | 40342 | YTPBUIWNJRGZFW-CGUMRCKQSA-N | O |  |  |  |
| 209 | 40486 | BGHCVCJVXZWKCC-UHFFFAOYSA-N |  | O |  |  |
| 210 | 40682 | MNWFXJYAOYHMED-UHFFFAOYSA-N | O |  |  |  |
| 211 | 44193 | XZLOJOSQFFJQBP-BOEDYSQBSA-N | O |  |  |  |
| 212 | 44194 | VCMVZLIPJZNIBV-CLXHPGAVSA-N | O |  |  |  |
| 213 | 50181 | CATSNJVOTSVZJV-UHFFFAOYSA-N | O |  |  |  |
| 214 | 50206 | MBIPADCEHSKJDQ-PBOSXPJTSA-N |  |  |  | O |
| 215 | 50249 | WGTRJVCFDUCKCM-FMKGYKFTSA-N | O |  |  |  |
| 216 | 50352 | JXNPEDYJTDQORS-HZJYTTRNSA-N | O |  |  |  |
| 217 | 50597 | UXMKOLJFXKKWEQ-UHFFFAOYSA-N | O |  |  |  |
| 218 | 50735 | TVCXVUHHCUYLGX-UHFFFAOYSA-N | O |  |  |  |
| 219 | 53206 | MBIPADCEHSKJDQ-MUYACECFSA-N |  |  |  | O |
| 220 | 59900 | SFJOMLIUSIKKRA-MYYUVRNCSA-N | O |  |  |  |
| 221 | 60148 | YDCUKUFFVMOSKZ-JNIPTGCTSA-N | O |  |  |  |
| 222 | 67524 | KATXJJSCAPBIOB-UHFFFAOYSA-N | O |  |  |  |
| 223 | 67526 | JJWIOXUMXIOXQN-UHFFFAOYSA-N | O |  |  |  |
| 224 | 68167 | RGTIBVZDHOMOKC-UHFFFAOYSA-N | O |  |  |  |
| 225 | 69821 | ZVDBUOGYYYNMQI-UHFFFAOYSA-N | O |  |  |  |
| 226 | 73498 | PVLHOJXLNBFHDX-XHJPDDKBSA-N |  | O |  |  |
| 227 | 74138 | SPURMHFLEKVAAS-UHFFFAOYSA-N | O |  |  |  |
| 228 | 79497 | KQSFNXMDCOFFGW-GNDIVNLPSA-N |  | O |  |  |
| 229 | 81029 | QMAYBMKBYCGXDH-KFWWJZLASA-N |  |  |  | O |
| 230 | 82861 | QOXUIQMPPDIDGM-PMOUVXMZSA-N |  |  |  | O |
| 231 | 84744 | FFDULTAFAQRACT-JSGUJALWSA-N | O |  |  |  |
| 232 | 86263 | NTYAVUNEPXGZQJ-KXJMYDKTSA-N |  | O |  |  |
| 233 | 86264 | XENTZWWGCWIEHN-HLJDGUNLSA-N | O |  |  |  |
| 234 | 89332 | YOMSJEATGXXYPX-UHFFFAOYSA-N | O |  |  |  |
| 235 | 90052 | HCXVJBMSMIARIN-PHZDYDNGSA-N |  | O |  |  |
| 236 | 90200 | YHBUQBJHSRGZNF-VGOFMYFVSA-N | O |  |  |  |
| 237 | 90201 | YHBUQBJHSRGZNF-AUWJEWJLSA-N | O |  |  |  |
| 238 | 90233 | HAJTYGKLQDXTPO-VNWUQRQDSA-N |  | O |  |  |
| 239 | 90352 | FTSINDMZMFBWFS-UHFFFAOYSA-N | O |  |  |  |
| 240 | 90481 | KMKFOIBUKYMVRJ-YHFBEQRYSA-N | O |  |  |  |
| 241 | 90582 | IEPGNWMPIFDNSD-HZJYTTRNSA-N | O |  |  |  |
| 242 | 90600 | SPCXZDDGSGTVAW-XIDUGBJDSA-N |  | O |  |  |
| 243 | 90639 | DMWNOMDRYALDDY-IAPVPJMBSA-N | O |  |  |  |
| 244 | 90767 | WKOLLVMJNQIZCI-UHFFFAOYSA-N | O |  |  |  |
| 245 | 90772 | GOHCSRAOXZPHFO-HUGMCNGHSA-N | O |  |  |  |
| 246 | 90971 | LGSKOQUJWNADCQ-UHFFFAOYSA-N | O |  |  |  |
| 247 | 91028 | QMAYBMKBYCGXDH-ZNMIVQPWSA-N |  |  |  | O |
| 248 | 91047 | NPNUFJAVOOONJE-GFUGXAQUSA-N |  | O |  |  |
| 249 | 91311 | UGJAEDFOKNAMQD-QXPKXGMISA-N |  | O |  |  |
| 250 | 91350 | WIONCQLWGYLTME-UHFFFAOYSA-N |  | O |  |  |
| 251 | 91470 | GXEGJTGWYVZSNR-SJRHNVSNSA-N | O |  |  |  |
| 252 | 91663 | YEGODGHIXCTKCQ-DJTSXUJQSA-N | O |  |  |  |
| 253 | 91744 | WAAGDSRYOTWUKB-UHFFFAOYSA-N | O |  |  |  |
| 254 | 91771 | YPZBIQKYSOYOGV-UHFFFAOYSA-N | O |  |  |  |
| 255 | 91833 | NSMRKFBAPAOVQL-FLIBITNWSA-N | O |  |  |  |
| 256 | 91848 | LTYLUDGDHUEBGX-UHFFFAOYSA-N | O |  |  |  |
| 257 | 91902 | JHWFWLUAUPZUCP-UHFFFAOYSA-N | O |  |  |  |
| 258 | 91935 | CFAKWWQIUFSQFU-UHFFFAOYSA-N | O |  |  |  |
| 259 | 91968 | BBNYCLAREVXOSG-UHFFFAOYSA-N | O |  |  |  |
| 260 | 92080 | KKSDGJDHHZEWEP-SNAWJCMRSA-N | O |  |  |  |
| 261 | 92233 | DWRXFEITVBNRMK-JXOAFFINSA-N | O |  |  |  |
| 262 | 92313 | WRHGORWNJGOVQY-RBSFLKMASA-N |  |  |  | O |
| 263 | 92487 | STRABSCAWZINIF-FGRDXJNISA-N | O |  |  |  |
| 264 | 92586 | UAUDZVJPLUQNMU-KTKRTIGZSA-N | O |  |  |  |
| 265 | 92807 | YOSRLTNUOCHBEA-SGVKAIFKSA-N | O |  |  |  |
| 266 | 92903 | VMOJIHDTVZTGDO-UHFFFAOYSA-N | O |  |  |  |
| 267 | 92923 | PMOWTIHVNWZYFI-AATRIKPKSA-N | O |  |  |  |
| 268 | 92947 | KENSDALTFWMRLW-RHRFKROSSA-N | O |  |  |  |
| 269 | 93030 | WWPCLIMUTNKTDY-UHFFFAOYSA-N | O |  |  |  |
| 270 | 93081 | FSRZGYRCMPZNJF-KHMAMNHCSA-N | O |  |  |  |
| 271 | 93152 | XSXIVVZCUAHUJO-UHFFFAOYSA-N | O |  |  |  |
| 272 | 93200 | FRMCCTDTYSRUBE-BGPZULBFSA-N | O |  |  |  |
| 273 | 93201 | ITYNGVSTWVVPIC-XVIXHAIJSA-N |  | O |  |  |
| 274 | 93203 | ITYNGVSTWVVPIC-DHGKCCLASA-N |  | O |  |  |
| 275 | 93205 | OPFTUNCRGUEPRZ-QLFBSQMISA-N |  | O |  |  |
| 276 | 93216 | GAIBLDCXCZKKJE-RXJOXMPGSA-N | O |  |  |  |
| 277 | 93273 | YGSDEFSMJLZEOE-UHFFFAOYSA-N |  |  |  | O |
| 278 | 93274 | XPORLJWHBRUXGD-HWIUFREPSA-N | O |  |  |  |
| 279 | 93484 | RDIMTXDFGHNINN-UHFFFAOYSA-N |  | O |  |  |
| 280 | 94164 | KWFJIXPIFLVMPM-PEGGXJLSSA-N | O |  |  |  |
| 281 | 94213 | QULNVKABFWNUCW-UHFFFAOYSA-N | O |  |  |  |
| 282 | 94334 | BXGVVQADPFXGHD-LSDHHAIUSA-N |  |  |  | O |
| 283 | 94460 | MRBZASPFZDNMCA-UHFFFAOYSA-N | O |  |  |  |
| 284 | 94507 | CUUMXRBKJIDIAY-ZDUSSCGKSA-N | O |  |  |  |
| 285 | 94715 | AEMOLEFTQBMNLQ-AQKNRBDQSA-N |  | O |  |  |
| 286 | 94791 | CCNJEDHOSPKCTA-UHFFFAOYSA-N | O |  |  |  |
| 287 | 94792 | PEZWXFPXPHFYEI-UHFFFAOYSA-N | O |  |  |  |
| 288 | 94881 | STGSRVGAMRCRDT-QWPWDPHFSA-N | O |  |  |  |
| 289 | 94882 | CBFRMOAISYCMQF-NCYSPSBASA-N | O |  |  |  |
| 290 | 94899 | YLHIMAHFOWREAD-UHFFFAOYSA-N | O |  |  |  |
| 291 | 94931 | FWCWPAUCBWOOGG-LEBRZWKMSA-N |  |  |  | O |
| 292 | 95012 | WJQOMUVKRDJBGZ-AKQSMOMZSA-N | O |  |  |  |
| 293 | 95234 | UMTHRBGGTLLPEZ-AWTIYEGFSA-N | O |  |  |  |
| 294 | 95266 | CZMPRYZUTNMZPT-JSJPIWTBSA-N | O |  |  |  |
| 295 | 95430 | NJUXRKMKOFXMRX-OOFHPJNRSA-N |  |  |  | O |
| 296 | 95462 | FQTLCLSUCSAZDY-SDNWHVSQSA-N | O |  |  |  |
| 297 | 95581 | FMKXOBHFNIMPLP-RIOAGLNTSA-N | O |  |  |  |
| 298 | 95584 | HRUIFVUXBZHKGJ-ADZKRXCWSA-N | O |  |  |  |
| 299 | 95585 | ZLNSNUFITCZIHR-SJSOZKBRSA-N | O |  |  |  |
| 300 | 95586 | ICGATYTYOILYQX-DXSAALQNSA-N | O |  |  |  |
| 301 | 95588 | SABMMBQQLJBUGT-HXVAASPGSA-N | O |  |  |  |
| 302 | 95590 | VOINPVINZHREBB-HXSDXVBYSA-N | O |  |  |  |
| 303 | 95591 | KEQXHOWXGVHEHV-NTBBOPETSA-N | O |  |  |  |
| 304 | 95592 | AZIGQTILUNTIQH-RJLRDNOXSA-N | O |  |  |  |
| 305 | 95594 | LTJNNBTXMKQJDS-UTYWKFPOSA-N | O |  |  |  |
| 306 | 95595 | MQHAPACHPNAQLC-VEQNPLAVSA-N | O |  |  |  |
| 307 | 95597 | LBEQBAUYSPUYAY-ZQQZZLNPSA-N | O |  |  |  |
| 308 | 95598 | QKDFXHKCEBRIBS-FWHCNOIFSA-N | O |  |  |  |
| 309 | 95664 | NKVWPVHNGDIGKX-IXUIVVTISA-N | O |  |  |  |
| 310 | 95665 | RWBRUCCWZPSBFC-RXRZZTMXSA-N | O |  |  |  |
| 311 | 95738 | VKANVHXFBJQXLP-OWIQXKKRSA-N | O |  |  |  |
| 312 | 95742 | MYBAONSAUGZRAX-UBQYYSLZSA-N | O |  |  |  |
| 313 | 95746 | QIFYDSMWESCVBZ-FAOXUISGSA-N | O |  |  |  |
| 314 | 95747 | DKDROENXDOYZAS-MYTAREJTSA-N | O |  |  |  |
| 315 | 95943 | BINCVPIVQCXZAB-GKEBJNQESA-N | O |  |  |  |
| 316 | 95944 | PYRYQDQZMLGNFX-QLJZFXKWSA-N | O |  |  |  |
| 317 | 95946 | SCGZGMGJBYDJHK-JIWUHWEISA-N | O |  |  |  |
| 318 | 95958 | WTARULDDTDQWMU-UHFFFAOYSA-N | O |  |  |  |
| 319 | 95959 | ZMDBYYWNZIGFOZ-JBDTYSNRSA-N | O |  |  |  |
| 320 | 95960 | VLEPDZWFKYPLFS-CWNWXWMDSA-N | O |  |  |  |
| 321 | 95961 | ARLABIIIHZJSOZ-CWNWXWMDSA-N | O |  |  |  |
| 322 | 96341 | VKOBVWXKNCXXDE-UHFFFAOYSA-N | O |  |  |  |
| 323 | 96391 | DDOPAXLQAFAFAZ-YZSKAGILSA-N | O |  |  |  |
| 324 | 96396 | NNBFRCZAUFQIAH-WCFZQSTRSA-N | O |  |  |  |
| 325 | 96421 | RWXIFXNRCLMQCD-JBVRGBGGSA-N |  | O |  |  |
| 326 | 96422 | RWXIFXNRCLMQCD-CZIWJLDFSA-N |  | O |  |  |
| 327 | 96425 | UJUDHUMABIRGHD-RJIRWEFGSA-N | O |  |  |  |
| 328 | 96448 | ZTQSADJAYQOCDD-FDDSVCGKSA-N | O |  |  |  |
| 329 | 96449 | YNBYFOIDLBTOMW-QHNUHGIDSA-N | O |  |  |  |
| 330 | 96757 | CJFGBCWGOQRURQ-JFJIKBJRSA-N | O |  |  |  |
| 331 | 97042 | YLMBQJRKOKVUCP-HTVDCONZSA-N |  |  | O |  |
| 332 | 97463 | KEEWIHDTSNESJZ-ZJHVPRRPSA-N | O |  |  |  |
| 333 | 97464 | SRLWVHOYELSQPG-SHLRTLSOSA-N | O |  |  |  |
| 334 | 97465 | KPMBCGORMVBETN-RAMNPTJZSA-N | O |  |  |  |
| 335 | 97466 | CTFUVKZOEJTLHY-MGGVZZALSA-N | O |  |  |  |
| 336 | 97467 | RFTKPDHUKGCCEV-KMVKTPQPSA-N | O |  |  |  |
| 337 | 97468 | FZKCAHBNCSORRJ-XKUZKHRGSA-N | O |  |  |  |
| 338 | 97469 | UPWZFNVFBFZSOV-WKTNQJLESA-N | O |  |  |  |
| 339 | 97470 | FNBJJDRFUOJIFP-VLUHFQMZSA-N | O |  |  |  |
| 340 | 98191 | LHGVFZTZFXWLCP-UHFFFAOYSA-N | O |  |  |  |
| 341 | 101731 | CSKINCSXMLCMAR-UHFFFAOYSA-N |  |  |  | O |
| 342 | 110848 | NLHRRMKILFRDGV-UHFFFAOYSA-N | O |  |  |  |
| 343 | 111446 | UYPPHUAQDGUVKN-UHFFFAOYSA-N | O |  |  |  |
| 344 | 131039 | ZEQZCZRDJPTCHI-MWTAGDMHSA-N |  |  |  | O |
| 345 | 131256 | JDCPEKQWFDWQLI-LUQKBWBOSA-N |  | O |  |  |
| 346 | 142619 | AVTXSAWPGCSYFO-ZCHNTGFUSA-N |  |  |  | O |
| 347 | 157711 | AGBCLJAHARWNLA-DQUQINEDSA-N |  | O |  |  |
| 348 | 157712 | PEOKIFXHILHWMQ-HQKIIMAWSA-N | O |  |  |  |
| 349 | 158501 | VKYVIIIEJKSVBR-XHJPDDKBSA-N | O |  |  |  |
| 350 | 159022 | VPDZRSSKICPUEY-SUYBHAEQSA-N |  | O |  |  |
| 351 | 162967 | OSXWNRAKZUNVDR-QHEGXUBCSA-N |  | O |  |  |
| 352 | 162969 | FWCWPAUCBWOOGG-HCSCSRTKSA-N |  |  |  | O |
| 353 | 171401 | JSNRRGGBADWTMC-QINSGFPZSA-N |  |  |  | O |
| 354 | 200213 | SGNBVLSWZMBQTH-ZRUUVFCLSA-N |  |  |  | O |
| 355 | 202884 | UGMDQWNVJMIQKD-LJJUAEGKSA-N | O |  |  |  |
| 356 | 213350 | PYXFVCFISTUSOO-HKUCOEKDSA-N |  | O |  |  |
| 357 | 280934 | DTOSIQBPPRVQHS-PDBXOOCHSA-N | O |  |  |  |
| 358 | 283280 | GVLDSGIQZAFIAN-IXDOHACOSA-N |  |  |  | O |
| 359 | 283316 | NDFKTBCGKNOHPJ-AATRIKPKSA-N | O |  |  |  |
| 360 | 283349 | JZQKTMZYLHNFPL-UHFFFAOYSA-N | O |  |  |  |
| 361 | 283375 | QFPVVMKZTVQDTL-BQYQJAHWSA-N | O |  |  |  |
| 362 | 300379 | FNIRVWPHRMMRQI-PGOMJGFXSA-N |  | O |  |  |
| 363 | 301086 | LHORCXXUZJAMPU-UHFFFAOYSA-N | O |  |  |  |
| 364 | 301105 | LULQAZFDPUGEBU-UHFFFAOYSA-N | O |  |  |  |
| 365 | 301314 | OZHQNSFDJIIDJA-DANXVBPVSA-N | O |  |  |  |
| 366 | 301316 | JCFOXVKGSJUKTN-LJCGQYBJSA-N | O |  |  |  |
| 367 | 301386 | LDIAQNKCRRXZCD-JKIFXVOUSA-N |  | O |  |  |
| 368 | 301387 | JFTBERIHMIFXML-YYNUNSROSA-N | O |  |  |  |
| 369 | 301858 | DLIKSSGEMUFQOK-SFTVRKLSSA-N | O |  |  |  |
| 370 | 301908 | LLXVPTXOKTYXHU-HUGMCNGHSA-N | O |  |  |  |
| 371 | 301921 | JJVNINGBHGBWJH-UHFFFAOYSA-N | O |  |  |  |
| 372 | 301984 | PBNFQEYKZLUTLH-UHFFFAOYSA-N | O |  |  |  |
| 373 | 301993 | FMMOOAYVCKXGMF-MURFETPASA-N | O |  |  |  |
| 374 | 302704 | UOJAEODBOCLNBU-GYMUUCMZSA-N | O |  |  |  |
| 375 | 303083 | QZIBYWPRLPFUFP-UHFFFAOYSA-N | O |  |  |  |
| 376 | 304979 | AYXPYQRXGNDJFU-FEPPMDFBSA-N | O |  |  |  |
| 377 | 311382 | FJEHCEACNKMECD-XNNFINAPSA-N | O |  |  |  |
| 378 | 311566 | MKZIRHIVARSBHI-VHDGCEQUSA-N | O |  |  |  |
| 379 | 317632 | FTXZFRIHQNXZNH-UHFFFAOYSA-N |  | O |  |  |
| 380 | 317633 | MORPELUWUARUFU-UHFFFAOYSA-N |  | O |  |  |
| 381 | 317634 | WNVDKDQMWFSCPI-UHFFFAOYSA-N |  | O |  |  |
| 382 | 317635 | WDZQEROINMBCOK-UHFFFAOYSA-N |  | O |  |  |
| 383 | 328779 | QFJUYMMIBFBOJY-XBDFBJNCSA-N |  | O |  |  |
| 384 | 363097 | TWWSXAKLFHYWQT-NSCUHMNNSA-N | O |  |  |  |
| 385 | 364716 | IPIOINHYLACFOP-KTKRTIGZSA-N | O |  |  |  |
| 386 | 365075 | IGOWHGRNPLFNDJ-ZPHPHTNESA-N | O |  |  |  |
| 387 | 365678 | TYEPJNKESLRTEJ-AVQMFFATSA-N | O |  |  |  |
| 388 | 371839 | PHDRQUPMFWWDKK-AUWJEWJLSA-N | O |  |  |  |
| 389 | 377671 | NTWIHRCNQPXEOZ-KUOIDRQISA-N | O |  |  |  |
| 390 | 377673 | YXTDIKURRYLWJF-MNYRRJMLSA-N | O |  |  |  |
| 391 | 377674 | NNGLJDNEBXZMHP-UCXJXVQFSA-N | O |  |  |  |
| 392 | 412555 | AVXFIVJSCUOFNT-QXPABTKOSA-N | O |  |  |  |
| 393 | 412556 | XBGLCVZQMWKHFC-NMQALWILSA-N | O |  |  |  |
| 394 | 431297 | RXORHYFDDNAOQS-MDZDMXLPSA-N | O |  |  |  |
| 395 | 432497 | RMZHSBMIZBMVMN-YSSOQSIOSA-N |  | O |  |  |
| 396 | 432709 | SVURIXNDRWRAFU-OGMFBOKVSA-N | O |  |  |  |
| 397 | 440259 | NEEQFPMRODQIKX-REOHCLBHSA-N | O |  |  |  |
| 398 | 441033 | WQZGKKKJIJFFOK-QTVWNMPRSA-N |  | O |  |  |
| 399 | 441920 | YURJSTAIMNSZAE-OVQXMHPVSA-N |  | O |  |  |
| 400 | 441921 | PWAOOJDMFUQOKB-WCZZMFLVSA-N |  | O |  |  |
| 401 | 441922 | CDBSSEPBXCNOMO-UWASIUMJSA-N | O |  |  |  |
| 402 | 441923 | YURJSTAIMNSZAE-HHNZYBFYSA-N |  | O |  |  |
| 403 | 441925 | AWKIKXPYYLORMJ-RZGHNGSCSA-N | O |  |  |  |
| 404 | 441931 | DKQXJFKAGOIKTJ-VIFTWHOHSA-N | O |  |  |  |
| 405 | 441932 | VFRFTROKXBCTHW-VIFTWHOHSA-N | O |  |  |  |
| 406 | 441933 | DIRCSSBCBKSLIV-GFODNYKDSA-N | O |  |  |  |
| 407 | 441935 | AWKIKXPYYLORMJ-LFBJWFFQSA-N | O |  |  |  |
| 408 | 441936 | BBKYATCKVYLQSJ-STYIPFKDSA-N | O |  |  |  |
| 409 | 441937 | FMRIUCIDETWQJA-OMCHRGAGSA-N | O |  |  |  |
| 410 | 451129 | NPZAABKZLIBPQV-FDUYOZAUSA-N | O |  |  |  |
| 411 | 467801 | DSVMWGREWREVQQ-IRXDYDNUSA-N | O |  |  |  |
| 412 | 468733 | SHCBCKBYTHZQGZ-CJPZEJHVSA-N |  | O |  |  |
| 413 | 472238 | WMGBQZAELMGYNO-YMWSGFAJSA-N | O |  |  |  |
| 414 | 476330 | QTGAEXCCAPTGLB-UOAUIWSESA-N |  |  |  | O |
| 415 | 481990 | FVIZARNDLVOMSU-IRFFNABBSA-N | O |  |  |  |
| 416 | 499198 | KWDWBAISZWOAHD-MHOSXIPRSA-N | O |  |  |  |
| 417 | 522487 | WNNYSRHBDYRXCH-UHFFFAOYSA-N | O |  |  |  |
| 418 | 534398 | GLKQAHXBJLGAFT-UHFFFAOYSA-N | O |  |  |  |
| 419 | 534931 | CCMWYZQSEJEAEY-UHFFFAOYSA-N | O |  |  |  |
| 420 | 535296 | MIJOZYYZCMBCHF-UHFFFAOYSA-N | O |  |  |  |
| 421 | 536573 | BXKXWMWZABIJFN-UHFFFAOYSA-N | O |  |  |  |
| 422 | 536617 | NVADVJRRZSUKPI-UHFFFAOYSA-N | O |  |  |  |
| 423 | 560878 | SEWYHOMCDKWYEF-UHFFFAOYSA-N | O |  |  |  |
| 424 | 560917 | ISEQOSOYOGJYOG-UHFFFAOYSA-N | O |  |  |  |
| 425 | 566139 | WHMSWWROVUQISG-UHFFFAOYSA-N | O |  |  |  |
| 426 | 576719 | DNDLHRMVKMCSOS-UHFFFAOYSA-N | O |  |  |  |
| 427 | 582313 | VQDSDYULHIDXKS-UHFFFAOYSA-N | O |  |  |  |
| 428 | 584555 | ZRBFCAALKKNCJG-SJYBZOGZSA-N | O |  |  |  |
| 429 | 584557 | MIDMLXVYLNWDTG-ZUHWWPGTSA-N | O |  |  |  |
| 430 | 592853 | HERICYNRBVMDFO-WVYXDZFESA-N | O |  |  |  |
| 431 | 592854 | XEEFSHTUESCNEP-ICNXTIMXSA-N |  |  |  | O |
| 432 | 592855 | IRNPMKFRLSGTLR-NZHQOURKSA-N | O |  |  |  |
| 433 | 595094 | BUDWHMNUSAOQBI-SNPRPXQTSA-N |  |  |  | O |
| 434 | 595133 | LTMKWDWWHXRNMO-UHFFFAOYSA-N |  |  |  | O |
| 435 | 599928 | OZTXYFOXQFKYRP-TXRYYSRHSA-N |  | O |  |  |
| 436 | 608819 | RRDUCLRMPBYJEE-SJYBZOGZSA-N | O |  |  |  |
| 437 | 608820 | KMWSTRPSDZFSMW-QXIMOFHJSA-N | O |  |  |  |
| 438 | 618812 | WGUFHMCIXUZUAL-JLMCIHFGSA-N | O |  |  |  |
| 439 | 618813 | UDOFLRJQZVKUBL-JLMCIHFGSA-N | O |  |  |  |
| 440 | 618814 | DFNOHNMHWQVJHX-JLMCIHFGSA-N | O |  |  |  |
| 441 | 625116 | AOXSLJSDFVRCQA-RCWAPVLCSA-N | O |  |  |  |
| 442 | 625117 | RNNSAPJUQOXCLU-QLJIQFCGSA-N | O |  |  |  |
| 443 | 638015 | NCYCYZXNIZJOKI-OVSJKPMPSA-N | O |  |  |  |
| 444 | 638072 | YYGNTYWPHWGJRM-AAJYLUCBSA-N | O |  |  |  |
| 445 | 652021 | YDVZWJBDTALPRC-RZONFAIUSA-N | O |  |  |  |
| 446 | 652174 | YQUSSKADRHYVPT-QTYVTQAZSA-N | O |  |  |  |
| 447 | 668381 | CKUVNOCSBYYHIS-IRFFNABBSA-N |  | O |  |  |
| 448 | 668382 | CKUVNOCSBYYHIS-SUEBGMEDSA-N |  | O |  |  |
| 449 | 668383 | IMABIBADXHMGRS-DCVWDPKKSA-N | O |  |  |  |
| 450 | 668384 | IMABIBADXHMGRS-AOBITMBASA-N | O |  |  |  |
| 451 | 701272 | LLPWNQMSUYAGQI-OOSPGMBYSA-N |  |  |  | O |
| 452 | 706587 | QWRTXOOFEHOROQ-ORFOJOBXSA-N | O |  |  |  |
| 453 | 717750 | XYDGLABNHGXTRN-BHLNDSQSSA-N | O |  |  |  |
| 454 | 721561 | RLDVZILFNVRJTL-IWFVLDDISA-N |  | O |  |  |
| 455 | 736266 | SYNBBWLEYQBFQT-NTCAYCPXSA-N | O |  |  |  |
| 456 | 750025 | WVNIISADYSWCOG-MSSDKOIGSA-N | O |  |  |  |
| 457 | 800075 | FPRYGNYXOFHMLF-UHFFFAOYSA-N | O |  |  |  |
| 458 | 809542 | XNGXWSFSJIQMNC-FIYORUNESA-N |  |  |  | O |
| 459 | 839223 | PHLXREOMFNVWOH-DNQFHFKUSA-N |  | O |  |  |
| 460 | 855920 | RAQNTCRNSXYLAH-RFCGZQMISA-N |  | O |  |  |
| 461 | 855921 | RAQNTCRNSXYLAH-PQYWRUIPSA-N |  | O |  |  |
| 462 | 865919 | SVONIFWOMWTUKZ-FIYORUNESA-N | O |  |  |  |
| 463 | 865921 | OQXSQYIOYDUOSH-RHKQBYAPSA-N | O |  |  |  |
| 464 | 887590 | KWRQPASKWCJCPI-DOGUGFTJSA-N | O |  |  |  |
| 465 | 887678 | HJRVLGWTJSLQIG-ABNMXWHVSA-N | O |  |  |  |
| 466 | 895488 | QOMBXPYXWGTFNR-KRPFXEAISA-N |  |  |  | O |
| 467 | 895489 | ZVTVWDXRNMHGNY-JOGTXEPTSA-N | O |  |  |  |
| 468 | 895492 | JZGSAQYOKZSOSY-KCOPIVCESA-N | O |  |  |  |
| 469 | 895499 | SMPCFRCKHGOLAS-YPQQTJRSSA-N | O |  |  |  |
| 470 | 898279 | GZYPWOGIYAIIPV-JBDTYSNRSA-N |  | O |  |  |
| 471 | 900379 | WWZKQHOCKIZLMA-UHFFFAOYSA-N | O |  |  |  |
| 472 | 911092 | QUNSGRLNZDSQJC-SNJLJHFRSA-N |  | O |  |  |
| 473 | 913110 | BOUBBPHZUPRWIO-YUIUBULTSA-N | O |  |  |  |
| 474 | 913111 | UTRFMMWCNUHHBK-FGTMMUONSA-N | O |  |  |  |
| 475 | 913112 | UQEBOJRXTNLPKZ-KHPPLWFESA-N | O |  |  |  |
| 476 | 913113 | OOLOUZMPEFSKTD-DTQAZKPQSA-N | O |  |  |  |
| 477 | 917976 | NODILNFGTFIURN-GZPRDHCNSA-N |  | O |  |  |
| 478 | 918692 | SWIROVJVGRGSPO-JBVRGBGGSA-N | O |  |  |  |
| 479 | 918693 | GNSLNJGTRVKMGX-LRDVQNGZSA-N | O |  |  |  |
| 480 | 918694 | GNSLNJGTRVKMGX-UPWCVFCHSA-N | O |  |  |  |
| 481 | 941542 | KVMXBSSOCCPAOR-WWJNHZDPSA-N |  | O |  |  |
| 482 | 941543 | UEBIBJSWHIZNCA-BGPUAMRSSA-N |  | O |  |  |
| 483 | 945294 | BTRPYCUFCMLYGV-YRPJOIJRSA-N | O |  |  |  |
| 484 | 951890 | INSOWUHKGVUHAZ-VCNUQGIMSA-N | O |  |  |  |
| 485 | 973057 | ZCJQJJWNFDNQGZ-SNPRPXQTSA-N |  |  |  | O |
| 486 | 973934 | LKQMMFFQYMYQOJ-QVHKTLOISA-N | O |  |  |  |
| 487 | 986191 | LKZDFKLGDGSGEO-PABQPRPFSA-N | O |  |  |  |
| 488 | 996452 | AYXPYQRXGNDJFU-IMNVLQEYSA-N | O |  |  |  |

**Table S7 List of ginseng compounds in TCMSP and their existence in TM-MC and TCMID.**

| No | ID | INCHIKEY | Only TCMSP | TCMSP, TM-MC, and TCMID | TCMSP and TM-MC | TCMSP and TCMID |
| --- | --- | --- | --- | --- | --- | --- |
| 1 | MOL000029 | HAVYZKHVTLAPDZ-PPGMXFKZSA-N |  |  |  | O |
| 2 | MOL000035 | YOVSPTNQHMDJAG-QLFBSQMISA-N |  | O |  |  |
| 3 | MOL000036 | NPNUFJAVOOONJE-GFUGXAQUSA-N |  | O |  |  |
| 4 | MOL000066 | ITYNGVSTWVVPIC-PDWCTOEPSA-N |  | O |  |  |
| 5 | MOL000069 | IPCSVZSSVZVIGE-UHFFFAOYSA-N |  | O |  |  |
| 6 | MOL000269 | BPLQKQKXWHCZSS-UHFFFAOYSA-N |  |  |  | O |
| 7 | MOL000358 | KZJWDPNRJALLNS-VJSFXXLFSA-N |  | O |  |  |
| 8 | MOL000422 | IYRMWMYZSQPJKC-UHFFFAOYSA-N |  | O |  |  |
| 9 | MOL000449 | HCXVJBMSMIARIN-PHZDYDNGSA-N |  | O |  |  |
| 10 | MOL000628 | VWDLOXMZIGUBKM-CQJGLIEWSA-N |  |  |  | O |
| 11 | MOL000676 | DOIRQSBPFJWKBE-UHFFFAOYSA-N |  |  |  | O |
| 12 | MOL000749 | OYHQOLUKZRVURQ-AVQMFFATSA-N |  |  | O |  |
| 13 | MOL000787 | GPTFURBXHJWNHR-UHFFFAOYSA-N | O |  |  |  |
| 14 | MOL000864 | YCOZIPAWZNQLMR-UHFFFAOYSA-N |  | O |  |  |
| 15 | MOL000874 | UILPJVPSNHJFIK-UHFFFAOYSA-N | O |  |  |  |
| 16 | MOL000879 | FLIACVVOZYBSBS-UHFFFAOYSA-N |  | O |  |  |
| 17 | MOL000886 | BGHCVCJVXZWKCC-UHFFFAOYSA-N |  | O |  |  |
| 18 | MOL000908 | OPFTUNCRGUEPRZ-QLFBSQMISA-N |  | O |  |  |
| 19 | MOL000935 | SPCXZDDGSGTVAW-XIDUGBJDSA-N |  | O |  |  |
| 20 | MOL000942 | LHYHMMRYTDARSZ-BYNSBNAKSA-N |  |  | O |  |
| 21 | MOL000968 | XZRVRYFILCSYSP-HNNXBMFYSA-N |  |  |  | O |
| 22 | MOL001212 | HLZKNKRTKFSKGZ-UHFFFAOYSA-N |  |  |  | O |
| 23 | MOL001218 | LQZZUXJYWNFBMV-UHFFFAOYSA-N | O |  |  |  |
| 24 | MOL001312 | SECPZKHBENQXJG-BQYQJAHWSA-N |  | O |  |  |
| 25 | MOL001392 | ZAZKJZBWRNNLDS-UHFFFAOYSA-N | O |  |  |  |
| 26 | MOL001396 | WQEPLUUGTLDZJY-UHFFFAOYSA-N |  | O |  |  |
| 27 | MOL001641 | WTTJVINHCBCLGX-NQLNTKRDSA-N |  |  | O |  |
| 28 | MOL001706 | QEOHJVNDENHRCH-VQCYPWCPSA-N | O |  |  |  |
| 29 | MOL001738 | OFOBLEOULBTSOW-UHFFFAOYSA-N | O |  |  |  |
| 30 | MOL001817 | HPEUJPJOZXNMSJ-UHFFFAOYSA-N | O |  |  |  |
| 31 | MOL001818 | IZFGRAGOVZCUFB-CMDGGOBGSA-N | O |  |  |  |
| 32 | MOL001819 | XIUXKAZJZFLLDQ-UHFFFAOYSA-N | O |  |  |  |
| 33 | MOL001949 | UGJAEDFOKNAMQD-QXPKXGMISA-N |  | O |  |  |
| 34 | MOL001965 | AQASRZOCERRGBL-ROJLCIKYSA-N |  |  |  | O |
| 35 | MOL002121 | VPDZRSSKICPUEY-AGJXFOQBSA-N |  | O |  |  |
| 36 | MOL002136 | UPJFTVFLSIQQAV-ONGXEEELSA-N |  |  |  | O |
| 37 | MOL002137 | TVMXDCGIABBOFY-UHFFFAOYSA-N |  |  |  | O |
| 38 | MOL002307 | DSTCZBGJCUOFLM-SXNKARFESA-N |  |  |  | O |
| 39 | MOL002312 | QNVPMKAFCVXMFH-NDDHQHNUSA-N |  |  |  | O |
| 40 | MOL002323 | OIRDTQYFTABQOQ-DEGSGYPDSA-N |  | O |  |  |
| 41 | MOL002377 | POQICXMTUPVZMX-BQCJVYABSA-N |  |  |  | O |
| 42 | MOL002526 | YHAJBLWYOIUHHM-GUTXKFCHSA-N |  |  |  | O |
| 43 | MOL002669 | SWIWTAJTJOYCTB-WNUOVGGOSA-N |  |  |  | O |
| 44 | MOL002879 | IJFPVINAQGWBRJ-UHFFFAOYSA-N | O |  |  |  |
| 45 | MOL003346 | YXBUQQDFTYOHQI-UHFFFAOYSA-N | O |  |  |  |
| 46 | MOL003648 | HUKSJTUUSUGIDC-BDJLRTHQSA-N | O |  |  |  |
| 47 | MOL003845 | WGEMODLYPVOIIW-QWHCGFSZSA-N |  |  |  | O |
| 48 | MOL003902 | RBKMRGOHCLRTLZ-KHPPLWFESA-N | O |  |  |  |
| 49 | MOL004100 | UASXFKHZBZLKLY-DHDCSXOGSA-N | O |  |  |  |
| 50 | MOL004174 | NOLWRMQDWRAODO-KKUMJFAQSA-N |  |  |  | O |
| 51 | MOL004237 | MXDMETWAEGIFOE-HUUCEWRRSA-N |  |  |  | O |
| 52 | MOL004275 | RKQDKXOBRXTSFS-UOAUIWSESA-N | O |  |  |  |
| 53 | MOL004492 | JRHJXXLCNATYLS-HYHFCDFPSA-N |  |  |  | O |
| 54 | MOL004498 | OQFVSKGDZKMZEH-WCYOEAGXSA-N |  |  |  | O |
| 55 | MOL004647 | SZHOJFHSIKHZHA-UHFFFAOYSA-N |  |  |  | O |
| 56 | MOL005269 | PZKNYJWHOZUWDF-OSRDXIQISA-N |  |  |  | O |
| 57 | MOL005270 | GOQYKNQRPGWPLP-UHFFFAOYSA-N |  |  |  | O |
| 58 | MOL005271 | UCIDYSLOTJMRAM-UHFFFAOYSA-N | O |  |  |  |
| 59 | MOL005272 | DZXBZPMJYIXTTI-UHFFFAOYSA-N | O |  |  |  |
| 60 | MOL005273 | VNOKAWVKCFUZGK-PUFFNIGPSA-N |  |  |  | O |
| 61 | MOL005274 | HNRMPXKDFBEGFZ-UHFFFAOYSA-N | O |  |  |  |
| 62 | MOL005275 | YFHGNGNLIWGTTR-CHWSQXEVSA-N | O |  |  |  |
| 63 | MOL005276 | DXHBSDCNOWJBQW-CHWSQXEVSA-N | O |  |  |  |
| 64 | MOL005277 | ZZEQNXPBKOFTBG-ACRUOGEOSA-N | O |  |  |  |
| 65 | MOL005278 | CJBFZKZYIPBBTO-UHFFFAOYSA-N | O |  |  |  |
| 66 | MOL005279 | RAQNTCRNSXYLAH-UQORERNTSA-N |  | O |  |  |
| 67 | MOL005281 | RAQNTCRNSXYLAH-SLRRGMQASA-N |  | O |  |  |
| 68 | MOL005283 | AGBCLJAHARWNLA-CBWWKSIWSA-N |  | O |  |  |
| 69 | MOL005284 | SHCBCKBYTHZQGZ-CJPZEJHVSA-N |  | O |  |  |
| 70 | MOL005285 | PYXFVCFISTUSOO-VUFVRDRTSA-N |  | O |  |  |
| 71 | MOL005286 | AGBCLJAHARWNLA-LVJGBEOWSA-N |  | O |  |  |
| 72 | MOL005287 | RWXIFXNRCLMQCD-LYTHEZCASA-N |  | O |  |  |
| 73 | MOL005289 | MAKRYGRRIKSDES-RKDXNWHRSA-N | O |  |  |  |
| 74 | MOL005290 | WXVQURJGDUNJCS-UHFFFAOYSA-N |  |  |  | O |
| 75 | MOL005291 | NUSHOJSYOLRGAX-IIAUIHIBSA-N |  |  |  | O |
| 76 | MOL005293 | HSOMNBKXPGCNBH-UHFFFAOYSA-N | O |  |  |  |
| 77 | MOL005294 | LAIUFBWHERIJIH-QMMMGPOBSA-N | O |  |  |  |
| 78 | MOL005295 | HTZWVZNRDDOFEI-GFCCVEGCSA-N | O |  |  |  |
| 79 | MOL005296 | UZTXSMATBUWDDZ-CYBMUJFWSA-N | O |  |  |  |
| 80 | MOL005297 | RCOXMOQVMZTXOO-UHFFFAOYSA-N | O |  |  |  |
| 81 | MOL005298 | KDUIMXINOLVPCT-AATRIKPKSA-N |  |  |  | O |
| 82 | MOL005299 | SQKZZFHVQCSUHZ-OAHLLOKOSA-N | O |  |  |  |
| 83 | MOL005300 | NTYAVUNEPXGZQJ-JPOZCNCBSA-N |  | O |  |  |
| 84 | MOL005301 | PYXFVCFISTUSOO-BFYBINCJSA-N |  | O |  |  |
| 85 | MOL005302 | ZVXASUNIAXYESJ-CPMIRZSNSA-N |  |  |  | O |
| 86 | MOL005303 | AFNWSIIBAYUTTL-UHFFFAOYSA-N | O |  |  |  |
| 87 | MOL005304 | UXCXDWDJBSJZOU-IOHFVGAQSA-N |  |  |  | O |
| 88 | MOL005305 | FHHSEFRSDKWJKJ-UHFFFAOYSA-N | O |  |  |  |
| 89 | MOL005306 | DHKHKXVYLBGOIT-UHFFFAOYSA-N | O |  |  |  |
| 90 | MOL005307 | VKYOVEAZNCJNGQ-HUEBKHCJSA-N |  |  |  | O |
| 91 | MOL005308 | UPWMWFSEBOFTNA-SBAKFKMJSA-N |  |  |  | O |
| 92 | MOL005309 | KQSFNXMDCOFFGW-OUFZWZSTSA-N |  | O |  |  |
| 93 | MOL005310 | MIJYXULNPSFWEK-JZQYXDLISA-N |  |  | O |  |
| 94 | MOL005311 | NLEHZOMZLCBMKF-GOYIYKMHSA-N |  |  |  | O |
| 95 | MOL005313 | YBJHBAHKTGYVGT-ZXFLCMHBSA-N | O |  |  |  |
| 96 | MOL005314 | LSYKFBZWBDMZLQ-OAQYLSRUSA-N |  |  |  | O |
| 97 | MOL005315 | NEHNMFOYXAPHSD-SNVBAGLBSA-N |  |  |  | O |
| 98 | MOL005316 | AEMOLEFTQBMNLQ-BYHBOUFCSA-N |  | O |  |  |
| 99 | MOL005317 | WRCBXHDQHPUVHW-CDRKEARJSA-N |  |  |  | O |
| 100 | MOL005318 | SVZLTRRSGWXPBL-UHFFFAOYSA-N |  |  |  | O |
| 101 | MOL005319 | RYCNBIYTZSGSPI-UHFFFAOYSA-N |  |  |  | O |
| 102 | MOL005320 | YZXBAPSDXZZRGB-CGRWFSSPSA-N |  |  |  | O |
| 103 | MOL005321 | RFWULRHBGYKEEZ-UHFFFAOYSA-N |  |  |  | O |
| 104 | MOL005322 | RMZHSBMIZBMVMN-HUUCEWRRSA-N |  | O |  |  |
| 105 | MOL005323 | CYUTWBUOPDPXJL-FHZWBSINSA-N |  |  |  | O |
| 106 | MOL005325 | NFZYDZXHKFHPGA-UPULUGQVSA-N |  | O |  |  |
| 107 | MOL005326 | LDIAQNKCRRXZCD-UJQRMWHLSA-N |  | O |  |  |
| 108 | MOL005328 | KVMXBSSOCCPAOR-KHMVMXOBSA-N |  | O |  |  |
| 109 | MOL005329 | UEBIBJSWHIZNCA-MUXWUWCSSA-N |  | O |  |  |
| 110 | MOL005330 | QUNSGRLNZDSQJC-AYBHOFBPSA-N |  | O |  |  |
| 111 | MOL005331 | GZYPWOGIYAIIPV-MPBJDOIPSA-N |  | O |  |  |
| 112 | MOL005332 | PYXFVCFISTUSOO-WMQFBVARSA-N |  | O |  |  |
| 113 | MOL005333 | NODILNFGTFIURN-GAAOXUKQSA-N |  | O |  |  |
| 114 | MOL005334 | PYXFVCFISTUSOO-HKUCOEKDSA-N |  | O |  |  |
| 115 | MOL005335 | NODILNFGTFIURN-FCUDRQNNSA-N |  | O |  |  |
| 116 | MOL005336 | JDCPEKQWFDWQLI-ZCKGZTPXSA-N |  | O |  |  |
| 117 | MOL005337 | RLDVZILFNVRJTL-IWFVLDDISA-N |  | O |  |  |
| 118 | MOL005338 | PWAOOJDMFUQOKB-WCZZMFLVSA-N |  | O |  |  |
| 119 | MOL005340 | SHCBCKBYTHZQGZ-DLHMIPLTSA-N |  | O |  |  |
| 120 | MOL005341 | YURJSTAIMNSZAE-HHNZYBFYSA-N |  | O |  |  |
| 121 | MOL005342 | RWXIFXNRCLMQCD-DRJVPZJKSA-N |  | O |  |  |
| 122 | MOL005344 | CKUVNOCSBYYHIS-IRFFNABBSA-N |  | O |  |  |
| 123 | MOL005345 | PHLXREOMFNVWOH-YAGNRYSRSA-N |  | O |  |  |
| 124 | MOL005347 | OZTXYFOXQFKYRP-IKDRHPKYSA-N |  | O |  |  |
| 125 | MOL005349 | ZCVPBYMQBJPBCT-WZCOPVJESA-N |  | O |  |  |
| 126 | MOL005350 | YLMBQJRKOKVUCP-QVKAQDGOSA-N |  | O |  |  |
| 127 | MOL005351 | FTXZFRIHQNXZNH-YESZJQIVSA-N |  | O |  |  |
| 128 | MOL005352 | MORPELUWUARUFU-ULQDDVLXSA-N |  | O |  |  |
| 129 | MOL005353 | WNVDKDQMWFSCPI-ULQDDVLXSA-N |  | O |  |  |
| 130 | MOL005354 | WDZQEROINMBCOK-YESZJQIVSA-N |  | O |  |  |
| 131 | MOL005355 | WIONCQLWGYLTME-DLBZAZTESA-N |  | O |  |  |
| 132 | MOL005356 | GAEQWKVGMHUUKO-UHFFFAOYSA-N |  |  |  | O |
| 133 | MOL005357 | XDVOVYYAPHHHBE-YGIMCSHQSA-N |  |  |  | O |
| 134 | MOL005358 | ODBLHEXUDAPZAU-VVJJHMBFSA-N |  |  |  | O |
| 135 | MOL005359 | ODBLHEXUDAPZAU-OKKQSCSOSA-N |  |  |  | O |
| 136 | MOL005360 | DTMIMKTZETWDJV-DUUKBJRLSA-N |  |  |  | O |
| 137 | MOL005361 | UOFHLCPZXZURFL-RJBDEUDKSA-N |  | O |  |  |
| 138 | MOL005362 | PYXFVCFISTUSOO-SPPDXTJESA-N |  | O |  |  |
| 139 | MOL005363 | OSXWNRAKZUNVDR-HUOLQKEOSA-N |  | O |  |  |
| 140 | MOL005365 | GUBGYTABKSRVRQ-QUYVBRFLSA-N |  |  |  | O |
| 141 | MOL005366 | HPSSZFFAYWBIPY-UHFFFAOYSA-N |  |  |  | O |
| 142 | MOL005367 | WQZGKKKJIJFFOK-RWOPYEJCSA-N |  | O |  |  |
| 143 | MOL005368 | VORKGRIRMPBCCZ-UHFFFAOYSA-N | O |  |  |  |
| 144 | MOL005369 | FZRGCIPZQGXDCM-OAIDTJHVSA-N |  |  |  | O |
| 145 | MOL005370 | HNXNKTMIVROLTK-UHFFFAOYSA-N | O |  |  |  |
| 146 | MOL005371 | IAABKQHJKGRFAU-MNOVXSKESA-N |  |  |  | O |
| 147 | MOL005372 | FNIRVWPHRMMRQI-CVTXWOHCSA-N |  | O |  |  |
| 148 | MOL005374 | YPUHYSBFIMWSEC-FSMABQBOSA-N |  |  |  | O |
| 149 | MOL005375 | OIZXRZCQJDXPFO-UHFFFAOYSA-N | O |  |  |  |
| 150 | MOL005376 | PVLHOJXLNBFHDX-BMTJSWHCSA-N |  | O |  |  |
| 151 | MOL005378 | RDIMTXDFGHNINN-IXDOHACOSA-N |  | O |  |  |
| 152 | MOL005379 | VREZDOWOLGNDPW-ALTGWBOUSA-N |  |  |  | O |
| 153 | MOL005380 | STKZKAJIJHJDCQ-HPKJDOSXSA-N |  |  |  | O |
| 154 | MOL005381 | ZSKGQVFRTSEPJT-UHFFFAOYSA-N |  |  |  | O |
| 155 | MOL005382 | GEZCJRBINSDUSC-UHFFFAOYSA-N |  |  |  | O |
| 156 | MOL005383 | XDSSPSLGNGIIHP-VKHMYHEASA-N |  |  |  | O |
| 157 | MOL005384 | GVNUFBXIXQNOCF-CRNMQVKPSA-N |  |  |  | O |
| 158 | MOL005385 | RHHKXYIFKCWYMH-MVWJERBFSA-N | O |  |  |  |
| 159 | MOL005386 | NGPDZEACIWDCKX-WUDKWMPASA-N |  |  |  | O |
| 160 | MOL005388 | WLUQEGDKTQZXBV-QWHCGFSZSA-N | O |  |  |  |
| 161 | MOL005389 | RVSTWRHIGKXTLG-WCXIOVBPSA-N | O |  |  |  |
| 162 | MOL005390 | GHOKWGTUZJEAQD-SSDOTTSWSA-N |  |  |  | O |
| 163 | MOL005391 | CXENHBSYCFFKJS-LOQWIJHWSA-N |  | O |  |  |
| 164 | MOL005392 | MJJZVOIXKVUBQD-YISVQYQQSA-N |  |  |  | O |
| 165 | MOL005394 | OJYKYCDSGQGTRJ-GQYWAMEOSA-N |  |  |  | O |
| 166 | MOL005396 | BSLAWKTUBVGEDH-UNQGMJICSA-N |  |  |  | O |
| 167 | MOL005397 | OORMXZNMRWBSTK-XYCZMWNLSA-N | O |  |  |  |
| 168 | MOL005398 | NPJICTMALKLTFW-FYZSWKHYSA-N |  | O |  |  |
| 169 | MOL005400 | VNVVACHTDGQAHA-DONNJNOYSA-N |  |  | O |  |
| 170 | MOL005402 | HUEBIMLTDXKIPR-UHFFFAOYSA-N | O |  |  |  |
| 171 | MOL005403 | VCNKUCWWHVTTBY-YTMGNKDFSA-N | O |  |  |  |
| 172 | MOL005404 | PXWNPQIYWRFPGW-ILQXODQXSA-N |  |  |  | O |
| 173 | MOL006651 | VGSYCWGXBYZLLE-QEEQPWONSA-N |  |  |  | O |
| 174 | MOL007500 | QFJUYMMIBFBOJY-UXZRXANASA-N |  | O |  |  |
| 175 | MOL011400 | UZIOUZHBUYLDHW-XUBRWZAZSA-N |  | O |  |  |

Table S8 List of ginseng compounds in TCMID and their existence in TM-MC and TCMSP.

| No | ID | INCHIKEY | Only TCMID | TCMID, TM-MC, and TCMSP | TCMID and TM-MC | TCMID and TCMSP |
| --- | --- | --- | --- | --- | --- | --- |
| 1 | 617 | DMAFLSPHXHVGDJ-YUMQZZPRSA-N |  |  | O |  |
| 2 | 618 | OIRDTQYFTABQOQ-KQYNXXCUSA-N |  | O |  |  |
| 3 | 626 | ZKHQWZAMYRWXGA-CRKDRTNXSA-N |  |  |  | O |
| 4 | 928 | ITYNGVSTWVVPIC-DHGKCCLASA-N |  | O |  |  |
| 5 | 1534 | JJNVDCBKBUSUII-IHTQCXBNSA-N | O |  |  |  |
| 6 | 1608 | KQSFNXMDCOFFGW-QIUYVDLSSA-N |  | O |  |  |
| 7 | 1674 | NLEHZOMZLCBMKF-TXHKITKMSA-N |  |  |  | O |
| 8 | 2352 | VPDZRSSKICPUEY-MBTGMABISA-N |  | O |  |  |
| 9 | 2395 | BPARNYZCFXOEKY-ZETCQYMHSA-N | O |  |  |  |
| 10 | 2412 | XZRVRYFILCSYSP-OAHLLOKOSA-N |  |  |  | O |
| 11 | 2849 | QMAYBMKBYCGXDH-KFWWJZLASA-N |  |  | O |  |
| 12 | 2851 | WRHGORWNJGOVQY-RRFJBIMHSA-N |  |  | O |  |
| 13 | 2852 |  |  |  | O |  |
| 14 | 2943 | MBIPADCEHSKJDQ-LAKVINJISA-N |  |  | O |  |
| 15 | 3040 | CPTCHGRDJMOIJZ-XHTKMIFXSA-N |  |  | O |  |
| 16 | 3041 | SWIWTAJTJOYCTB-NMYXBGBTSA-N |  |  |  | O |
| 17 | 3242 | INOSMXBKABLUIL-DCKDXSSCSA-N |  | O |  |  |
| 18 | 3354 |  | O |  |  |  |
| 19 | 3356 | LSYKFBZWBDMZLQ-UHFFFAOYSA-N |  |  |  | O |
| 20 | 3522 | ZICDJKZDHVLVOD-HUGMCNGHSA-N | O |  |  |  |
| 21 | 3523 | KQSFNXMDCOFFGW-GNDIVNLPSA-N |  | O |  |  |
| 22 | 3565 | BPRJTLAULHNDLP-UHFFFAOYSA-N | O |  |  |  |
| 23 | 3589 | OEYIOHPDSNJKLS-UHFFFAOYSA-N | O |  |  |  |
| 24 | 3592 | JRHJXXLCNATYLS-OMSIYMKDSA-N |  |  |  | O |
| 25 | 3766 | KRKNYBCHXYNGOX-UHFFFAOYSA-N | O |  |  |  |
| 26 | 3767 | NEHNMFOYXAPHSD-SNVBAGLBSA-N |  |  |  | O |
| 27 | 4679 | NPJICTMALKLTFW-MSNYQJDCSA-N |  | O |  |  |
| 28 | 4684 | AQASRZOCERRGBL-IADGFXSZSA-N |  |  |  | O |
| 29 | 5177 | RTZKSTLPRTWFEV-OLZOCXBDSA-N | O |  |  |  |
| 30 | 5180 | WRCBXHDQHPUVHW-KBXBIZAPSA-N |  |  |  | O |
| 31 | 5370 | YGSIRXHFAUFUEJ-GPTQDWHKSA-N | O |  |  |  |
| 32 | 5371 | SVZLTRRSGWXPBL-UHFFFAOYSA-N |  |  |  | O |
| 33 | 5402 | JKRZOJADNVOXPM-UHFFFAOYSA-N | O |  |  |  |
| 34 | 5403 | DOIRQSBPFJWKBE-IUWMYWAXSA-N |  |  |  | O |
| 35 | 6359 | CRNGFKXWIYTEPH-UHFFFAOYSA-N | O |  |  |  |
| 36 | 6521 | NLZUEZXRPGMBCV-UHFFFAOYSA-N |  |  | O |  |
| 37 | 6540 | GDNCXORZAMVMIW-UHFFFAOYSA-N |  |  | O |  |
| 38 | 6541 | CQKHFONAFZDDKV-VAWYXSNFSA-N | O |  |  |  |
| 39 | 6719 | DIOQZVSQGTUSAI-UHFFFAOYSA-N |  |  | O |  |
| 40 | 6740 | OPFTUNCRGUEPRZ-QLFBSQMISA-N |  | O |  |  |
| 41 | 6741 | BQSLMQNYHVFRDT-LSDHHAIUSA-N |  |  | O |  |
| 42 | 6742 | MXDMETWAEGIFOE-CABCVRRESA-N |  |  |  | O |
| 43 | 6744 | BPLQKQKXWHCZSS-UHFFFAOYSA-N |  |  |  | O |
| 44 | 7103 | FLWGFJVPYVWJCO-DLBZAZTESA-N | O |  |  |  |
| 45 | 7729 | JSNRRGGBADWTMC-NTCAYCPXSA-N |  |  | O |  |
| 46 | 7851 | OVBPIULPVIDEAO-LBPRGKRZSA-N | O |  |  |  |
| 47 | 7970 | FZRJRIPXNVGOHZ-HSUXUTPPSA-N | O |  |  |  |
| 48 | 7972 | RFWULRHBGYKEEZ-UHFFFAOYSA-N |  |  |  | O |
| 49 | 8408 | QOXUIQMPPDIDGM-PMOUVXMZSA-N |  |  | O |  |
| 50 | 8409 | QLPKLNOLHUEWSE-TTWOHQMCSA-N |  |  | O |  |
| 51 | 8410 | QOMBXPYXWGTFNR-KRPFXEAISA-N |  |  | O |  |
| 52 | 8411 | QCCQLRNQOAQMKU-JECDWVISSA-N | O |  |  |  |
| 53 | 8412 |  |  |  | O |  |
| 54 | 8413 |  | O |  |  |  |
| 55 | 8414 | NJUXRKMKOFXMRX-RNCAKNGISA-N |  |  | O |  |
| 56 | 8415 | QCCQLRNQOAQMKU-JECDWVISSA-N | O |  |  |  |
| 57 | 8416 |  | O |  |  |  |
| 58 | 8417 | CYUTWBUOPDPXJL-BESZVSAISA-N |  |  |  | O |
| 59 | 8418 |  |  | O |  |  |
| 60 | 8419 | KVMXBSSOCCPAOR-WWJNHZDPSA-N |  | O |  |  |
| 61 | 8420 | UEBIBJSWHIZNCA-BGPUAMRSSA-N |  | O |  |  |
| 62 | 8421 | QUNSGRLNZDSQJC-SNJLJHFRSA-N |  | O |  |  |
| 63 | 8422 | GZYPWOGIYAIIPV-JBDTYSNRSA-N |  | O |  |  |
| 64 | 8423 | NODILNFGTFIURN-GZPRDHCNSA-N |  | O |  |  |
| 65 | 8424 |  |  |  | O |  |
| 66 | 8425 | JDCPEKQWFDWQLI-USDRARRQSA-N |  | O |  |  |
| 67 | 8426 | RLDVZILFNVRJTL-IWFVLDDISA-N |  | O |  |  |
| 68 | 8427 | PWAOOJDMFUQOKB-UXYDDKAJSA-N |  | O |  |  |
| 69 | 8428 | UZIOUZHBUYLDHW-XUBRWZAZSA-N |  | O |  |  |
| 70 | 8429 | YURJSTAIMNSZAE-ZSRLCDFVSA-N |  | O |  |  |
| 71 | 8430 | AGBCLJAHARWNLA-DQUQINEDSA-N |  | O |  |  |
| 72 | 8431 |  |  | O |  |  |
| 73 | 8433 | RAQNTCRNSXYLAH-PQYWRUIPSA-N |  | O |  |  |
| 74 | 8434 | RAQNTCRNSXYLAH-TXZQGQPKSA-N |  | O |  |  |
| 75 | 8435 | CKUVNOCSBYYHIS-SUEBGMEDSA-N |  | O |  |  |
| 76 | 8436 |  |  |  | O |  |
| 77 | 8437 | QKZVLGNPEUWYDR-HWJAGOLRSA-N |  |  | O |  |
| 78 | 8438 | OZTXYFOXQFKYRP-ZYWVKJCFSA-N |  | O |  |  |
| 79 | 8444 | NFZYDZXHKFHPGA-QQHDHSITSA-N |  | O |  |  |
| 80 | 8445 | ZCVPBYMQBJPBCT-MUEIFEFKSA-N |  | O |  |  |
| 81 | 8446 | ZCVPBYMQBJPBCT-DGAXUDEFSA-N |  | O |  |  |
| 82 | 8447 | FTXZFRIHQNXZNH-UHFFFAOYSA-N |  | O |  |  |
| 83 | 8448 | MORPELUWUARUFU-UHFFFAOYSA-N |  | O |  |  |
| 84 | 8449 | WNVDKDQMWFSCPI-UHFFFAOYSA-N |  | O |  |  |
| 85 | 8450 | WDZQEROINMBCOK-LRUHZDSUSA-N |  | O |  |  |
| 86 | 8451 | WIONCQLWGYLTME-UHFFFAOYSA-N |  | O |  |  |
| 87 | 8452 | ANMYKTVFNJJBPX-UHFFFAOYSA-N | O |  |  |  |
| 88 | 8455 | GAEQWKVGMHUUKO-UHFFFAOYSA-N |  |  |  | O |
| 89 | 8754 | FBFMBWCLBGQEBU-RXMALORBSA-N |  |  | O |  |
| 90 | 8755 | JUGNSCDMIZVJCB-DELNEQSJSA-N |  |  |  | O |
| 91 | 8760 | AEMOLEFTQBMNLQ-QIUUJYRFSA-N |  | O |  |  |
| 92 | 8905 | ZWRRJEICIPUPHZ-DAOPMYJZSA-N | O |  |  |  |
| 93 | 8906 | BKGUPIVDQHHVMV-LSHKVNPSSA-N |  |  |  | O |
| 94 | 9040 | ADIDQIZBYUABQK-RWMBFGLXSA-N | O |  |  |  |
| 95 | 9070 |  |  |  | O |  |
| 96 | 9087 | SPCXZDDGSGTVAW-RFVSLCSESA-N |  | O |  |  |
| 97 | 9377 | NDJKXXJCMXVBJW-UHFFFAOYSA-N |  |  | O |  |
| 98 | 9378 | GOQYKNQRPGWPLP-GZXVCZRGSA-N |  |  |  | O |
| 99 | 9484 | VHOGADCBMMJONL-UHFFFAOYSA-N |  |  | O |  |
| 100 | 9485 | AOAFVSVYRSUDBN-UHFFFAOYSA-N | O |  |  |  |
| 101 | 9488 | DSTCZBGJCUOFLM-COECVNONSA-N |  |  |  | O |
| 102 | 9668 |  |  |  | O |  |
| 103 | 9669 |  |  |  |  | O |
| 104 | 9670 | QTGAEXCCAPTGLB-ZYEZJADKSA-N |  |  | O |  |
| 105 | 11329 | ODBLHEXUDAPZAU-VVJJHMBFSA-N |  |  |  | O |
| 106 | 11330 | ODBLHEXUDAPZAU-OKKQSCSOSA-N |  |  |  | O |
| 107 | 11331 |  | O |  |  |  |
| 108 | 11332 |  | O |  |  |  |
| 109 | 11333 | UPJFTVFLSIQQAV-ONGXEEELSA-N |  |  |  | O |
| 110 | 12017 | IYRMWMYZSQPJKC-UHFFFAOYSA-M |  | O |  |  |
| 111 | 12885 |  | O |  |  |  |
| 112 | 13277 | UPGLJTCDRBIZKP-KYOSRNDESA-N | O |  |  |  |
| 113 | 13278 | PZKNYJWHOZUWDF-YPRXJGMQSA-N |  |  |  | O |
| 114 | 13412 | BJEPYKJPYRNKOW-UHFFFAOYSA-N | O |  |  |  |
| 115 | 13414 | DTMIMKTZETWDJV-PBBAXLBGSA-N |  |  |  | O |
| 116 | 13434 | FWCWPAUCBWOOGG-HCSCSRTKSA-N |  |  | O |  |
| 117 | 13435 | UOFHLCPZXZURFL-GPDZXBECSA-N |  | O |  |  |
| 118 | 13436 | OSXWNRAKZUNVDR-QHEGXUBCSA-N |  | O |  |  |
| 119 | 13437 |  | O |  |  |  |
| 120 | 13447 | GUBGYTABKSRVRQ-LUOHZERUSA-N |  |  |  | O |
| 121 | 13500 | GZCGUPFRVQAUEE-UHFFFAOYSA-N |  |  | O |  |
| 122 | 13501 | WQZGKKKJIJFFOK-RWOPYEJCSA-N |  | O |  |  |
| 123 | 13772 | XHXUANMFYXWVNG-ADEWGFFLSA-N | O |  |  |  |
| 124 | 13915 | ZBDTYWHJRWZCPN-ZWKOTPCHSA-N | O |  |  |  |
| 125 | 15132 | QMAYBMKBYCGXDH-KKUMJFAQSA-N |  |  | O |  |
| 126 | 15134 | FZRGCIPZQGXDCM-IZZDOVSWSA-N |  |  |  | O |
| 127 | 15360 | ZCJQJJWNFDNQGZ-SNPRPXQTSA-N |  |  | O |  |
| 128 | 15361 |  |  |  | O |  |
| 129 | 15403 | DPQYOKVMVCQHMY-TUVASFSCSA-N | O |  |  |  |
| 130 | 15519 | PVNIIMVLHYAWGP-UHFFFAOYSA-N | O |  |  |  |
| 131 | 15520 | OQFVSKGDZKMZEH-QUMGVHPFSA-N |  |  |  | O |
| 132 | 15651 | IGGUPRCHHJZPBS-UHFFFAOYSA-N | O |  |  |  |
| 133 | 15653 | DAGYDJHSDMRWJX-WDYNHAJCSA-N |  |  |  | O |
| 134 | 15827 | ZBXDHDDTAIOMHK-UHFFFAOYSA-N |  |  | O |  |
| 135 | 15828 | FNIRVWPHRMMRQI-AWISTCESSA-N |  | O |  |  |
| 136 | 15830 | RTUMUBQEOSTPHN-VEIIDDJFSA-N |  |  | O |  |
| 137 | 15831 | YPUHYSBFIMWSEC-JODPPCSYSA-N |  |  |  | O |
| 138 | 15963 | NUJGJRNETVAIRJ-COJKEBBMSA-N |  |  | O |  |
| 139 | 15964 | TVMXDCGIABBOFY-UHFFFAOYSA-N |  |  |  | O |
| 140 | 16549 | QHZLMUACJMDIAE-UHFFFAOYSA-N | O |  |  |  |
| 141 | 16571 | COZUZLCKOUNNJQ-BABYYJQJSA-N | O |  |  |  |
| 142 | 16572 | ZRXJARPSARBQCO-UHFFFAOYSA-N | O |  |  |  |
| 143 | 16577 | ZEQZCZRDJPTCHI-JCHYFPDLSA-N |  |  | O |  |
| 144 | 16578 | ZEQZCZRDJPTCHI-JCHYFPDLSA-N |  |  | O |  |
| 145 | 16579 |  |  |  | O |  |
| 146 | 16580 | WHXUZXDWQKUIJL-UHFFFAOYSA-N |  |  | O |  |
| 147 | 16581 | VNLATJUGAZKQEH-IAGOWNOFSA-N | O |  |  |  |
| 148 | 16582 | PVLHOJXLNBFHDX-JVHPXEDNSA-N |  | O |  |  |
| 149 | 16583 | QFJUYMMIBFBOJY-AQIAMZLMSA-N |  | O |  |  |
| 150 | 16584 | UAOBSVDFJSNTLJ-UHFFFAOYSA-N | O |  |  |  |
| 151 | 16585 | GVLDSGIQZAFIAN-YJEKIOLLSA-N |  |  | O |  |
| 152 | 16586 | UGJAEDFOKNAMQD-MQNTZWLQSA-N |  | O |  |  |
| 153 | 16587 | RDIMTXDFGHNINN-IKGGRYGDSA-N |  | O |  |  |
| 154 | 16589 | VREZDOWOLGNDPW-HPORCNTFSA-N |  |  |  | O |
| 155 | 16590 | STKZKAJIJHJDCQ-RETZBPDQSA-N |  |  |  | O |
| 156 | 16695 | CSKINCSXMLCMAR-UHFFFAOYSA-N |  |  | O |  |
| 157 | 16817 | YCOZIPAWZNQLMR-UHFFFAOYSA-N |  | O |  |  |
| 158 | 17967 | PYXFVCFISTUSOO-QRVZQXMZSA-N |  | O |  |  |
| 159 | 17968 | SHCBCKBYTHZQGZ-XUIOTSDSSA-N |  | O |  |  |
| 160 | 17969 | ZAALQOFZFANFTF-UHFFFAOYSA-N | O |  |  |  |
| 161 | 18011 | OKZBCSRPGFBBBG-HBNUGNGRSA-N |  |  | O |  |
| 162 | 18014 |  | O |  |  |  |
| 163 | 18216 | BPHGMWVJJCWDPB-UHFFFAOYSA-N | O |  |  |  |
| 164 | 18260 | ZSKGQVFRTSEPJT-UHFFFAOYSA-N |  |  |  | O |
| 165 | 18267 | LCTONWCANYUPML-UHFFFAOYSA-N | O |  |  |  |
| 166 | 18435 | SNHCPECPLQRJNL-GWNBYJSOSA-N |  |  | O |  |
| 167 | 18511 | MUPFEKGTMRGPLJ-DSUVSBNISA-N | O |  |  |  |
| 168 | 18724 | SHZGCJCMOBCMKK-HGVZOGFYSA-N | O |  |  |  |
| 169 | 18819 | AUNGANRZJHBGPY-SCRDCRAPSA-N | O |  |  |  |
| 170 | 19172 | YGSDEFSMJLZEOE-UHFFFAOYSA-N |  |  | O |  |
| 171 | 19173 |  |  |  | O |  |
| 172 | 19291 | PDEQKAVEYSOLJX-PUVAYXBBSA-N | O |  |  |  |
| 173 | 19666 | ZQODGIAJYWHYRF-SWLSCSKDSA-N | O |  |  |  |
| 174 | 19671 | OZQAPQSEYFAMCY-RBSFLKMASA-N |  |  | O |  |
| 175 | 19672 | YOVSPTNQHMDJAG-QLFBSQMISA-N |  | O |  |  |
| 176 | 19673 |  |  | O |  |  |
| 177 | 19676 | XDSSPSLGNGIIHP-VKHMYHEASA-N |  |  |  | O |
| 178 | 19967 | KZJWDPNRJALLNS-VJSFXXLFSA-N |  | O |  |  |
| 179 | 19973 |  | O |  |  |  |
| 180 | 19974 |  | O |  |  |  |
| 181 | 19975 |  | O |  |  |  |
| 182 | 20130 | RQFVSMCFDFGRDX-UHFFFAOYSA-N | O |  |  |  |
| 183 | 20131 | PFNFFQXMRSDOHW-BFGUONQLSA-N | O |  |  |  |
| 184 | 20353 | GYDIPQRLDOTECG-PYURGGGNSA-N |  | O |  |  |
| 185 | 20357 |  | O |  |  |  |
| 186 | 20358 | QCQUFLUHCFYSFG-HTQKDBJGSA-N | O |  |  |  |
| 187 | 20359 |  | O |  |  |  |
| 188 | 20362 | SQDYRCMAEQXKCQ-BGPOZWQASA-N | O |  |  |  |
| 189 | 20428 | ZEDCKNAPOUCJFY-UHFFFAOYSA-N | O |  |  |  |
| 190 | 20429 | GVNUFBXIXQNOCF-LZYBPNLTSA-N |  |  |  | O |
| 191 | 20430 | CZMRCDWAGMRECN-FXFGBNHJSA-N | O |  |  |  |
| 192 | 20435 | QNVPMKAFCVXMFH-NDDHQHNUSA-N |  |  |  | O |
| 193 | 20699 | KWWCVCFQHGKOMI-UHFFFAOYSA-N | O |  |  |  |
| 194 | 21024 | HFDVRLIODXPAHB-UHFFFAOYSA-N |  | O |  |  |
| 195 | 21025 | HLZKNKRTKFSKGZ-UHFFFAOYSA-N |  |  |  | O |
| 196 | 21594 | IIYFAKIEWZDVMP-UHFFFAOYSA-N |  |  | O |  |
| 197 | 21618 | JPUKWEQWGBDDQB-WVTKYERHSA-N |  |  | O |  |
| 198 | 22236 | DRTQHJPVMGBUCF-AYZDMWBASA-N |  |  | O |  |
| 199 | 22538 | WQRHEUJLQBIGEO-UHFFFAOYSA-M | O |  |  |  |
| 200 | 22539 | GHOKWGTUZJEAQD-ZETCQYMHSA-M |  |  |  | O |
| 201 | 22540 | AGVAZMGAQJOSFJ-UHFFFAOYSA-M | O |  |  |  |
| 202 | 22541 | ZQTHOIGMSJMBLM-BUJSFMDZSA-N | O |  |  |  |
| 203 | 22643 | BXGVVQADPFXGHD-CABCVRRESA-N |  |  | O |  |
| 204 | 22827 | SRBFZHDQGSBBOR-OWMBCFKOSA-N | O |  |  |  |
| 205 | 23034 |  |  |  | O |  |
| 206 | 23038 | FLIACVVOZYBSBS-GZXVCZRGSA-N |  | O |  |  |
| 207 | 23066 | RWXIFXNRCLMQCD-NESGGLTLSA-N |  | O |  |  |
| 208 | 23085 |  |  | O |  |  |
| 209 | 23117 | YURJSTAIMNSZAE-ZUJJWFDPSA-N |  | O |  |  |
| 210 | 23129 |  |  | O |  |  |
| 211 | 23150 | NPNUFJAVOOONJE-IZZDOVSWSA-N |  | O |  |  |
| 212 | 23173 | MXDMETWAEGIFOE-GJZGRUSLSA-N |  |  |  | O |
| 213 | 23175 | LGOPTUPXVVNJFH-UHFFFAOYSA-N |  | O |  |  |
| 214 | 23181 | VRZCOXDMMILAJA-UHFFFAOYSA-N |  | O |  |  |
| 215 | 23219 |  |  | O |  |  |
| 216 | 23271 | PYXFVCFISTUSOO-OIORDRSNSA-N |  | O |  |  |
| 217 | 23273 |  |  |  | O |  |
| 218 | 23279 | OPFTUNCRGUEPRZ-QLFBSQMISA-N |  | O |  |  |
| 219 | 23379 | WQEPLUUGTLDZJY-UHFFFAOYSA-M |  | O |  |  |
| 220 | 23506 | TVTCXPXLRKTHAU-UHFFFAOYSA-N | O |  |  |  |
| 221 | 23559 | YOVSPTNQHMDJAG-QLFBSQMISA-N |  | O |  |  |
| 222 | 23655 | SECPZKHBENQXJG-FPLPWBNLSA-M |  | O |  |  |
| 223 | 23717 | GGHMUJBZYLPWFD-CUZKYEQNSA-N |  |  | O |  |
| 224 | 23794 | KUVMKLCGXIYSNH-UHFFFAOYSA-N | O |  |  |  |
| 225 | 23805 | OJYKYCDSGQGTRJ-GQYWAMEOSA-N |  |  |  | O |
| 226 | 23887 | VWDLOXMZIGUBKM-PLWLBJPKSA-N |  |  |  | O |
| 227 | 23919 | LQERIDTXQFOHKA-UHFFFAOYSA-N |  |  | O |  |
| 228 | 24037 | FAMPSKZZVDUYOS-OLXWZMHJSA-N |  |  | O |  |
| 229 | 24070 | XZRVRYFILCSYSP-OAHLLOKOSA-N |  |  |  | O |
| 230 | 24248 | VGSYCWGXBYZLLE-NEVVRMFVSA-N |  |  |  | O |
| 231 | 24748 | XBUPCLXNEIRAEX-UHFFFAOYSA-N | O |  |  |  |
| 232 | 25050 |  | O |  |  |  |
| 233 | 25051 | NODILNFGTFIURN-GZPRDHCNSA-N |  | O |  |  |
| 234 | 25215 | NGPDZEACIWDCKX-WUDKWMPASA-N |  |  |  | O |
| 235 | 25306 | RMZHSBMIZBMVMN-LSDHHAIUSA-N |  | O |  |  |
| 236 | 25395 | VNOKAWVKCFUZGK-HOJYRBEBSA-N |  |  |  | O |
| 237 | 25574 |  |  |  |  | O |
| 238 | 25613 |  |  |  |  | O |
| 239 | 25614 | SPCXZDDGSGTVAW-KQSVBRPFSA-N |  | O |  |  |
| 240 | 25622 | YHAJBLWYOIUHHM-SSDMNJCBSA-N |  |  |  | O |
| 241 | 25667 | KFNNEUAROQLUQS-ZJDNFKTFSA-N |  |  |  | O |
| 242 | 25702 |  |  | O |  |  |
| 243 | 25703 |  |  | O |  |  |
| 244 | 25704 | OZTXYFOXQFKYRP-TXRYYSRHSA-N |  | O |  |  |
| 245 | 25705 | PHLXREOMFNVWOH-YAGNRYSRSA-N |  | O |  |  |
| 246 | 25706 | RAQNTCRNSXYLAH-AWJPBMSFSA-N |  | O |  |  |
| 247 | 25707 | RAQNTCRNSXYLAH-RFCGZQMISA-N |  | O |  |  |
| 248 | 25708 |  |  | O |  |  |
| 249 | 25709 |  |  | O |  |  |
| 250 | 25710 | YURJSTAIMNSZAE-HHNZYBFYSA-N |  | O |  |  |
| 251 | 25711 | UZIOUZHBUYLDHW-XUBRWZAZSA-N |  | O |  |  |
| 252 | 25712 | PWAOOJDMFUQOKB-QVPADXGFSA-N |  | O |  |  |
| 253 | 25713 | FBFMBWCLBGQEBU-UHFFFAOYSA-N |  |  | O |  |
| 254 | 25714 | JDCPEKQWFDWQLI-UHFFFAOYSA-N |  | O |  |  |
| 255 | 25715 | NODILNFGTFIURN-USYOXQFSSA-N |  | O |  |  |
| 256 | 25716 |  |  | O |  |  |
| 257 | 25717 | GZYPWOGIYAIIPV-JBDTYSNRSA-N |  | O |  |  |
| 258 | 25718 | IWDYQBDCEDNTDP-BHPIZNGBSA-N |  | O |  |  |
| 259 | 25719 | UEBIBJSWHIZNCA-BGPUAMRSSA-N |  | O |  |  |
| 260 | 25720 | KVMXBSSOCCPAOR-WWJNHZDPSA-N |  | O |  |  |
| 261 | 25814 | RYCNBIYTZSGSPI-UHFFFAOYSA-N |  |  |  | O |
| 262 | 25838 | WXVQURJGDUNJCS-UHFFFAOYSA-N |  |  |  | O |
| 263 | 26070 | OIRDTQYFTABQOQ-DGPXGRDGSA-N |  | O |  |  |
| 264 | 28530 |  |  |  | O |  |
| 265 | 29509 | KZJWDPNRJALLNS-JQEGLAGPSA-N |  | O |  |  |
| 266 | 30187 | SZHOJFHSIKHZHA-UHFFFAOYSA-N |  |  |  | O |
| 267 | 30624 | UPWMWFSEBOFTNA-UHFFFAOYSA-N |  |  |  | O |
| 268 | 30704 | NOLWRMQDWRAODO-SLTAFYQDSA-N |  |  |  | O |
| 269 | 30750 | NPNUFJAVOOONJE-IOMPXFEGSA-N |  | O |  |  |
| 270 | 30939 | YZXBAPSDXZZRGB-CGRWFSSPSA-N |  |  |  | O |
| 271 | 30956 | CXENHBSYCFFKJS-ZWWAVPAWSA-N |  | O |  |  |
| 272 | 30968 |  |  |  |  | O |
| 273 | 31037 | VOUCMBDNXOKLCQ-YATHHJDDSA-N |  |  |  | O |
| 274 | 31038 | NFZYDZXHKFHPGA-QQHDHSITSA-N |  | O |  |  |
| 275 | 31039 |  |  | O |  |  |
| 276 | 31040 | RAQNTCRNSXYLAH-AWJPBMSFSA-N |  | O |  |  |
| 277 | 31078 | VBBLHZOJAWSCSP-FPLPWBNLSA-N |  |  |  | O |
| 278 | 31122 | HAVYZKHVTLAPDZ-RCHZWEEESA-N |  |  |  | O |
| 279 | 31337 | ODBLHEXUDAPZAU-OKKQSCSOSA-N |  |  |  | O |
| 280 | 31338 | ODBLHEXUDAPZAU-OKKQSCSOSA-N |  |  |  | O |
| 281 | 31339 | ODBLHEXUDAPZAU-FONMRSAGSA-N |  |  |  | O |
| 282 | 31449 | JBYXPOFIGCOSSB-GOJKSUSPSA-N | O |  |  |  |
| 283 | 31483 | UOFHLCPZXZURFL-TYRBHPFPSA-N |  | O |  |  |
| 284 | 31484 | NTYAVUNEPXGZQJ-KXJMYDKTSA-N |  | O |  |  |
| 285 | 31485 | OSXWNRAKZUNVDR-RALFNDPZSA-N |  | O |  |  |
| 286 | 31489 | GUBGYTABKSRVRQ-ASMJPISFSA-N |  |  |  | O |
| 287 | 31490 | HPSSZFFAYWBIPY-UHFFFAOYSA-N |  |  |  | O |
| 288 | 31495 | AEMOLEFTQBMNLQ-BYHBOUFCSA-M |  | O |  |  |
| 289 | 31853 | SHCBCKBYTHZQGZ-UHFFFAOYSA-N |  | O |  |  |
| 290 | 31900 | GEZCJRBINSDUSC-UHFFFAOYSA-N |  |  |  | O |
| 291 | 31910 |  |  |  |  | O |
| 292 | 32225 |  |  |  |  | O |
| 293 | 32244 |  |  |  |  | O |

Table S9 List of marker compounds from Chinese pharmacopoeia and their existence in TM-MC, TCMSP, and TCMID.

| Latin | Korean | Chinese | Japanese | Compound | TMMC | TCMSP | TCMID |
| --- | --- | --- | --- | --- | --- | --- | --- |
| Achyranthis Radix | 우슬 | 牛膝 | ゴシツ | β-ecdysterone | O | O | O |
| Aconiti Kusnezoffii Tuber | 초오 | 草乌 |  | aconitine | O | O | O |
| Aconiti Kusnezoffii Tuber | 초오 | 草乌 |  | hypaconitine | O | O | O |
| Aconiti Kusnezoffii Tuber | 초오 | 草乌 |  | mesaconitine | O | O | O |
| Aconiti Lateralis Radix Preparata | 부자 | 附子 | ブシ | benzoylaconitine | O | O | O |
| Aconiti Lateralis Radix Preparata | 부자 | 附子 | ブシ | benzoylhypaconine | O | O | O |
| Aconiti Lateralis Radix Preparata | 부자 | 附子 | ブシ | benzoylmesaconine | O | O | O |
| Aconiti Tuber | 천오 | 川乌 |  | aconitine | O | O | O |
| Aconiti Tuber | 천오 | 川乌 |  | hypaconitine | O | O | O |
| Aconiti Tuber | 천오 | 川乌 |  | mesaconitine | O | O | O |
| Aesculi Semen | 사라자 | 娑罗子 |  | escin Ia | O |  |  |
| Akebiae Caulis | 목통 | 木通 | モクツウ | calceolarioside B | O | O | O |
| Akebiae Fructus | 예지자 | 预知子 |  | α-hederin | O |  | O |
| Alismatis Rhizoma | 택사 | 泽泻 | タクシャ | alisol B 23-acetate | O | O | O |
| Allii Bulbus | 대산 | 大蒜 |  | allicin | O | O | O |
| Aloe | 노회 | 芦荟 | アロエ | barbaloin | O | O | O |
| Alpiniae Officinari Rhizoma | 고량강 | 高良姜 | リョウキョウ | galangin | O | O | O |
| Amomi Fructus | 사인 | 砂仁 | シュクシャ | isobornyl acetate |  | O |  |
| Amomi Fructus Rotundus | 백두구 | 豆蔻 |  | eucalyptol | O | O | O |
| Andrographis Herba | 천심련 | 穿心莲 |  | andrographolide | O |  | O |
| Andrographis Herba | 천심련 | 穿心莲 |  | dehydroandrographolide | O |  | O |
| Anemarrhenae Rhizoma | 지모 | 知母 | チモ | mangiferin | O | O | O |
| Anemarrhenae Rhizoma | 지모 | 知母 | チモ | timosaponin BII | O | O | O |
| Anemones Raddeanae Rhizoma | 양두첨 | 两头尖 |  | raddeanin A | O | O | O |
| Angelicae Dahuricae Radix | 백지 | 白芷 | ビャクシ | imperatorin | O | O | O |
| Angelicae Gigantis Radix | 당귀 | 当归 |  | ferulic acid | O | O | O |
| Aquilariae Lignum | 침향 | 沉香 | ジンコウ | agarotetrol | O | O | O |
| Araliae Continentalis Radix | 독활 | 独活 | ドクカツ | columbianadin | O | O | O |
| Araliae Continentalis Radix | 독활 | 独活 | ドクカツ | osthole | O | O | O |
| Arctii Fructus | 우방자 | 牛蒡子 | ゴボウシ | arctiin | O | O | O |
| Ardisiae Japonicae Herba | 왜지차 | 矮地茶 |  | bengenin | O | O | O |
| Arecae Semen | 빈랑자 | 槟榔 | ビンロウジ | arecoline | O | O | O |
| Arisaematis Rhizoma | 천남성 | 天南星 | テンナンショウ | apigenin |  |  |  |
| Armeniacae Semen | 행인 | 苦杏仁 | キョウニン | amygdalin | O | O | O |
| Artemisiae Argyi Folium | 애엽 | 艾叶 | ガイヨウ | eucalyptol | O |  | O |
| Artemisiae Capillaris Herba | 인진호 | 茵陈 | インチンコウ | chlorogenic acid | O | O | O |
| Artemisiae Capillaris Herba | 인진호 | 茵陈 | インチンコウ | scoparone | O | O | O |
| Asiasari Radix et Rhizoma | 세신 | 细辛 | サイシン | asarinin | O | O | O |
| Asteris Radix et Rhizoma | 자완 | 紫菀 | シオン | shionone | O | O | O |
| Astragali Complanati Semen | 사원자 | 沙苑子 |  | complanatuside | O | O | O |
| Astragali Radix | 황기 | 黄芪 | オウギ | astragaloside IV | O | O | O |
| Astragali Radix | 황기 | 黄芪 | オウギ | calycosin-7-glucoside | O | O |  |
| Atractylodis Rhizoma | 창출 | 苍术 | ソウジュツ | atractylodin | O | O | O |
| Aucklandiae Radix | 목향 | 木香 | モッコウ | costunolide | O | O | O |
| Aucklandiae Radix | 목향 | 木香 | モッコウ | dehydrocostus lactone | O | O | O |
| Aurantii Fructus Immaturus | 지각 | 枳壳 |  | naringin | O | O | O |
| Aurantii Fructus Immaturus | 지각 | 枳壳 |  | neohesperidin | O | O | O |
| Belamcandae Rhizoma | 사간 | 射干 |  | irisflorentin | O | O | O |
| Benzoinum | 안식향 | 安息香 | アンソッコウ | benzoic acid | O | O | O |
| Bistortae Rhizoma | 권삼 | 拳参 |  | gallic acid | O | O | O |
| Bolbostemmatis Rhizoma | 토패모 | 土贝母 |  | tubeimoside I | O |  | O |
| Bomeolum | 용뇌 | 天然冰片 |  | borneol | O | O | O |
| Bovis Calculus | 우황 | 牛黄 | ゴオウ | bilirubin | O |  | O |
| Bovis Calculus | 우황 | 牛黄 | ゴオウ | cholic acid | O |  | O |
| Brassicae Semen | 개자 | 芥子 |  | sinapine thiocyanate | O |  | O |
| Breeae Herba | 소계 | 小蓟 |  | linarin | O | O | O |
| Bruceae Fructus | 아담자 | 鸦胆子 |  | oleic acid | O |  | O |
| Buddlejae Flos | 밀몽화 | 密蒙花 |  | linarin | O | O | O |
| Bupleuri Radix | 시호 | 柴胡 | サイコ | saikosaponin A | O | O | O |
| Bupleuri Radix | 시호 | 柴胡 | サイコ | saikosaponin D | O | O | O |
| Capsici Fructus | 고추 | 辣椒 | トウガラシ | capsaicin | O | O | O |
| Capsici Fructus | 고추 | 辣椒 | トウガラシ | dihydrocapsaicin | O | O | O |
| Carthami Flos | 홍화 | 红花 | コウカ | hydroxysafflor yellow A | O | O | O |
| Carthami Flos | 홍화 | 红花 | コウカ | kaempferol | O | O | O |
| Cassiae Semen | 결명자 | 决明子 | ケツメイシ | aurantio-obtusin | O | O | O |
| Cassiae Semen | 결명자 | 决明子 | ケツメイシ | chrysophanol | O | O | O |
| Catechu | 아차 | 儿茶 |  | catechin | O | O |  |
| Catechu | 아차 | 儿茶 |  | l-epicatechin | O | O | O |
| Centellae Herba | 적설초 | 积雪草 |  | asiaticoside | O | O | O |
| Centellae Herba | 적설초 | 积雪草 |  | madecassoside | O | O | O |
| Chaenomelis Fructus | 모과 | 木瓜 | モッカ | oleanolic acid | O | O | O |
| Chaenomelis Fructus | 모과 | 木瓜 | モッカ | ursolic acid | O | O | O |
| Chelidonii Herba | 백굴채 | 白屈菜 |  | chelerythrine chloride | O |  | O |
| Choerospondiatis Fructus | 광조 | 广枣 |  | gallic acid | O | O | O |
| Chrysanthemi Indici Flos | 감국 | 野菊花 |  | linarin | O | O | O |
| Chrysanthmi Flos | 국화 | 菊花 | キクカ | 3,5-di-O-caffeoylquinic acid | O | O |  |
| Chrysanthmi Flos | 국화 | 菊花 | キクカ | chlorogenic acid | O | O | O |
| Chrysanthmi Flos | 국화 | 菊花 | キクカ | cynaroside | O | O | O |
| Cimicifugae Rhizoma | 승마 | 升麻 | ショウマ | isoferulic acid | O | O | O |
| Cinnamomi Cortex | 육계 | 肉桂 | ケイヒ | cinnamaldehyde | O | O | O |
| Cinnamomi Ramulus | 계지 | 桂枝 | ケイシ | cinnamaldehyde | O | O | O |
| Cirsii Herba | 대계 | 大蓟 |  | pectolinarin | O | O | O |
| Cistanchis Herba | 육종용 | 肉苁蓉 | ニクジュヨウ | echinacoside | O | O | O |
| Cistanchis Herba | 육종용 | 肉苁蓉 | ニクジュヨウ | verbascoside | O | O | O |
| Citri Grandis Exocarpium | 화귤홍 | 化橘红 |  | naringin | O | O | O |
| Citri Sarcodactylis Fructus | 불수 | 佛手 |  | hesperidin | O |  | O |
| Citri Unshius Pericarpium | 진피 | 陈皮 | チンピ | hesperidin | O | O | O |
| Citri Unshius Pericarpium Immaturus | 청피 | 青皮 | セイヒ | hesperidin | O | O | O |
| Clematidis Radix | 위령선 | 威灵仙 | イレイセン | oleanolic acid | O | O |  |
| Cnidi Fructus | 사상자 | 蛇床子 | ジャショウシ | osthole | O | O | O |
| Cnidii Rhizoma | 천궁 | 川芎 | センキュウ | ferulic acid | O |  | O |
| Coicis Semen | 의이인 | 薏苡仁 | ヨクイニン | trioleoylglyceride | O |  | O |
| Coptidis Rhizoma | 황련 | 黄连 | オウレン | berberine | O | O | O |
| Coptidis Rhizoma | 황련 | 黄连 | オウレン | berberine chloride | O |  |  |
| Coptidis Rhizoma | 황련 | 黄连 | オウレン | coptisine | O | O | O |
| Coptidis Rhizoma | 황련 | 黄连 | オウレン | epiberberine | O | O | O |
| Coptidis Rhizoma | 황련 | 黄连 | オウレン | palmatine | O | O | O |
| Cordyceps | 동충하초 | 冬虫夏草 |  | adenosine | O |  | O |
| Corni Fructus | 산수유 | 山茱萸 | サンシュユ | loganin | O | O | O |
| Corni Fructus | 산수유 | 山茱萸 | サンシュユ | morroniside | O |  | O |
| Corydalis Bungeanae Herba | 고지정 | 苦地丁 |  | corynoline | O | O | O |
| Corydalis Decumbentis Rhizoma | 하천무 | 夏天无 |  | palmatine | O | O | O |
| Corydalis Decumbentis Rhizoma | 하천무 | 夏天无 |  | protopine | O | O | O |
| Corydalis Tuber | 현호색 | 延胡索 | エンゴサク | tetrahydropalmatine | O |  | O |
| Crataegi Folium | 산사엽 | 山楂叶 |  | hyperin | O | O | O |
| Crataegi Folium | 산사엽 | 山楂叶 |  | rutin | O | O | O |
| Crocus | 번홍화 | 西红花 | サフラン | crocin I | O | O | O |
| Crocus | 번홍화 | 西红花 | サフラン | crocin II | O | O | O |
| Crotonis Semen | 파두 | 巴豆 |  | 2-hydroxyadenosine | O | O | O |
| Curculiginis Rhizoma | 선모 | 仙茅 |  | curculigoside | O | O | O |
| Curcumae Longae Rhizoma | 강황 | 姜黄 | ウコン | curcumin | O |  | O |
| Cuscutae Semen | 토사자 | 菟丝子 |  | hyperin | O | O | O |
| Cyathulae Radix | 천우슬 | 川牛膝 |  | cyasterone | O | O | O |
| Cynanchi Paniculati Radix et Rhizoma | 서장경 | 徐长卿 |  | paeonol | O | O | O |
| Daturae Flos | 양금화 | 洋金花 |  | scopolamine | O | O | O |
| Dictamni Radicis Cortex | 백선피 | 白鲜皮 |  | fraxinellone | O | O | O |
| Dictamni Radicis Cortex | 백선피 | 白鲜皮 |  | obacunone | O | O | O |
| Dioscoreae Nipponicae Rhizoma | 천산룡 | 穿山龙 |  | dioscin | O |  | O |
| Dipsaci Radix | 속단 | 续断 |  | asperosaponin VI | O | O |  |
| Drynariae Rhizoma | 골쇄보 | 骨碎补 |  | naringin | O |  | O |
| Echinopsis Radix | 우주누로 | 禹州漏芦 |  | 2,2':5',2''-terthiophene | O | O |  |
| Ecliptae Herba | 한련초 | 墨旱莲 |  | wedelolactone | O | O | O |
| Elsholtziae Herba | 향유 | 香薷 |  | 5-isopropyl-2-methylphenol | O | O | O |
| Elsholtziae Herba | 향유 | 香薷 |  | thymol | O | O | O |
| Ephedrae Herba | 마황 | 麻黄 | マオウ | ephedrine | O | O | O |
| Ephedrae Herba | 마황 | 麻黄 | マオウ | pseudoephedrine | O | O | O |
| Epimedii Herba | 음양곽 | 淫羊藿 巫山淫羊藿 | インヨウカク | icariin | O | O | O |
| Equiseti Herba | 목적 | 木贼 |  | kaempferol | O | O | O |
| Eriobotryae Folium | 비파엽 | 枇杷叶 | ビワヨウ | oleanolic acid | O | O | O |
| Eriobotryae Folium | 비파엽 | 枇杷叶 | ビワヨウ | ursolic acid | O | O | O |
| Erycibae Caulis | 정공등 | 丁公藤 |  | scopoletin | O | O | O |
| Eucommiae Cortex | 두충 | 杜仲 | トチュウ | pinoresinol diglucoside | O |  | O |
| Eucommiae Folium | 두충엽 | 杜仲叶 |  | chlorogenic acid | O | O | O |
| Euphorbiae Humifusae Herba | 지금초 | 地锦草 |  | quercetin | O | O | O |
| Euphorbiae Kansui Radix | 감수 | 甘遂 |  | euphadienol | O | O | O |
| Euphorbiae Lathyridis Semen | 속수자 | 千金子 |  | euphorbiasteroid | O |  | O |
| Euphorbiae Pekinensis Radix | 대극 | 京大戟 |  | euphadienol | O | O | O |
| Evodiae Fructus | 오수유 | 吴茱萸 | ゴシュユ | evodiamine | O | O | O |
| Evodiae Fructus | 오수유 | 吴茱萸 | ゴシュユ | limonin | O | O | O |
| Evodiae Fructus | 오수유 | 吴茱萸 | ゴシュユ | rutaecarpine | O | O | O |
| Fagopyri Dibotryis Rhizoma | 금교맥 | 金荞麦 |  | l-epicatechin | O | O | O |
| Foeniculi Fructus | 회향 | 小茴香 | ウイキョウ | trans-anethole | O | O | O |
| Forsythiae Fructus | 연교 | 连翘 | レンギョウ | forsythoside A | O | O | O |
| Forsythiae Fructus | 연교 | 连翘 | レンギョウ | phillyrin | O | O | O |
| Fritillariae Cirrhosae Bulbus | 천패모 | 川贝母 |  | sipeimine | O | O | O |
| Fritillariae Pallidiflorae Bulbus | 이패모 | 伊贝母 |  | sipeimine | O | O | O |
| Fritillariae Pallidiflorae Bulbus | 이패모 | 伊贝母 |  | sipeimine-3β-D-glucoside | O | O |  |
| Fritillariae Thunbergii Bulbus | 절패모 | 浙贝母 |  | peimine | O | O | O |
| Fritillariae Thunbergii Bulbus | 절패모 | 浙贝母 |  | peiminine | O | O | O |
| Fritillariae Ussuriensis Bulbus | 평패모 | 平贝母 |  | peiminine | O | O | O |
| Galla Rhois | 오배자 | 五倍子 |  | gallic acid | O | O |  |
| Ganoderma | 영지 | 灵芝 |  | oleanolic acid |  |  |  |
| Gardeniae Fructus | 치자 | 栀子 | サンシシ | gardenoside | O | O | O |
| Genkwae Flos | 원화 | 芫花 |  | genkwanin | O | O | O |
| Gentianae Macrophyllae Radix | 진교 | 秦艽 | ジンギョウ | gentiopicroside | O | O | O |
| Gentianae Macrophyllae Radix | 진교 | 秦艽 | ジンギョウ | loganic acid | O | O | O |
| Gentianae Scabrae Radix et Rhizoma | 용담 | 龙胆 | リュウタン | gentiopicroside | O | O | O |
| Ginkgo Folium | 은행엽 | 银杏叶 |  | bilobalide | O | O | O |
| Ginkgo Folium | 은행엽 | 银杏叶 |  | ginkgolide A | O | O | O |
| Ginkgo Folium | 은행엽 | 银杏叶 |  | ginkgolide B | O | O | O |
| Ginkgo Folium | 은행엽 | 银杏叶 |  | ginkgolide C | O | O | O |
| Ginseng Folium | 인삼엽 | 人参叶 |  | ginsenoside Re | O | O | O |
| Ginseng Folium | 인삼엽 | 人参叶 |  | ginsenoside Rg1 | O | O | O |
| Ginseng Radix | 인삼 | 人参 | ニンジン | ginsenoside Rb1 | O | O | O |
| Ginseng Radix | 인삼 | 人参 | ニンジン | ginsenoside Re | O | O | O |
| Ginseng Radix | 인삼 | 人参 | ニンジン | ginsenoside Rg1 | O | O | O |
| Ginseng Radix Rubra | 홍삼 | 红参 | コウジン | ginsenoside Rb1 | O | O | O |
| Ginseng Radix Rubra | 홍삼 | 红参 | コウジン | ginsenoside Re | O | O | O |
| Ginseng Radix Rubra | 홍삼 | 红参 | コウジン | ginsenoside Rg1 | O | O | O |
| Glycyrrhizae Radix et Rhizoma | 감초 | 甘草 | カンゾウ | glycyrrhizic acid | O | O | O |
| Glycyrrhizae Radix et Rhizoma | 감초 | 甘草 | カンゾウ | liquiritin | O | O | O |
| Granati Cortex | 석류피 | 石榴皮 |  | ellagic acid | O | O |  |
| Hippophae Fructus | 사극 | 沙棘 |  | isorhamnetin | O | O |  |
| Hippophae Fructus | 사극 | 沙棘 |  | rutin | O | O |  |
| Homalomenae Rhizoma | 천년건 | 千年健 |  | linalool |  | O | O |
| Hyoscyami Semen | 천선자 | 天仙子 |  | hyoscyamine | O |  | O |
| Hyoscyami Semen | 천선자 | 天仙子 |  | scopolamine | O | O | O |
| Ilicis Chinensis Folium | 사계청 | 四季青 |  | pedunculoside | O | O | O |
| Illici Veri Fructus | 팔각회향 | 八角茴香 |  | anethole | O | O | O |
| Impatientis Semen | 급성자 | 急性子 |  | hosenkoside A | O |  |  |
| Impatientis Semen | 급성자 | 急性子 |  | hosenkoside K | O |  |  |
| Indigo Pulverata Levis | 청대 | 青黛 |  | indigo | O | O | O |
| Indigo Pulverata Levis | 청대 | 青黛 |  | indirubin | O | O | O |
| Isatidis Folium | 대청엽 | 大青叶 蓼大青叶 | | indigo | O | O | O |
| Isatidis Folium | 대청엽 | 大青叶 蓼大青叶 | | indirubin | O | O | O |
| Isatidis Radix | 판람근 | 板蓝根 |  | (R,S)-epigoitrin | O |  |  |
| Kochiae Fructus | 지부자 | 地肤子 |  | momordin Ic | O | O |  |
| Leonuri Herba | 익모초 | 益母草 | ヤクモソウ | leonurine | O | O | O |
| Leonuri Herba | 익모초 | 益母草 | ヤクモソウ | stachydrine | O | O | O |
| Leonuri Semen | 충위자 | 茺蔚子 |  | stachydrine | O |  | O |
| Lepidii seu Descurainiae Semen | 정력자 | 葶苈子 |  | quercetin-3-O-β-D-glucose-7-O-β-D-gentiobiosiden | O |  |  |
| Ligustici Tenuissimi Rhizoma et Radix | 고본 | 蒿本 | コウホン | ferulic acid | O | O | O |
| Ligustri Fructus | 여정실 | 女贞子 |  | nuezhenide | O | O | O |
| Linderae Radix | 오약 | 乌药 | ウヤク | linderane | O |  | O |
| Linderae Radix | 오약 | 乌药 | ウヤク | norisoboldine | O |  |  |
| Lini Semen | 아마인 | 亚麻子 |  | linoleic acid | O | O |  |
| Lini Semen | 아마인 | 亚麻子 |  | α-linolenic acid | O | O |  |
| Liquidambaris Fructus | 노로통 | 路路通 |  | betulonic acid | O | O | O |
| Liquidambaris Storax | 소합향 | 苏合香 |  | cinnamic acid | O | O | O |
| Lithospermi Radix | 자근 | 紫草 | シコン | shikonin | O | O | O |
| Lithospermi Radix | 자근 | 紫草 | シコン | β,β'-dimethylacrylalkannin | O |  | O |
| Lonicerae Japonicae Flos | 금은화 | 金银花 | キンギンカ | chlorogenic acid | O | O | O |
| Lonicerae Japonicae Flos | 금은화 | 金银花 | キンギンカ | cynaroside | O | O |  |
| Lycii Fructus | 구기자 | 枸杞子 | クコシ | trimethylglycine | O | O | O |
| Lysimachiae Herba | 금전초 | 金钱草 |  | kaempferol | O | O |  |
| Lysimachiae Herba | 금전초 | 金钱草 |  | quercetin | O | O |  |
| Magnoliae Cortex | 후박 | 厚朴 | コウボク | honokiol | O | O | O |
| Magnoliae Cortex | 후박 | 厚朴 | コウボク | magnolol | O | O | O |
| Magnoliae Flos | 신이 | 辛夷 | シンイ | magnolin | O |  | O |
| Mahoniae Caulis | 공로목 | 功劳木 |  | berberine | O |  | O |
| Mahoniae Caulis | 공로목 | 功劳木 |  | columbamine | O | O |  |
| Mahoniae Caulis | 공로목 | 功劳木 |  | jatrorrhizine | O | O | O |
| Mahoniae Caulis | 공로목 | 功劳木 |  | palmatine | O | O | O |
| Malvae Fructus | 동규자 | 冬葵果 |  | caffeic acid |  |  |  |
| Melandrii Herba | 왕불류행 | 王不留行 |  | vaccarin | O | O | O |
| Meliae Cortex | 고련피 | 苦楝皮 |  | toosendanin | O |  | O |
| Meliae Fructus | 천련자 | 川楝子 | センレンシ | toosendanin | O | O | O |
| Menispermi Rhizoma | 북두근 | 北豆根 |  | dauricine | O |  | O |
| Menispermi Rhizoma | 북두근 | 北豆根 |  | daurisoline | O | O | O |
| Mori Folium | 상엽 | 桑叶 |  | rutin | O | O | O |
| Morindae Radix | 파극천 | 巴戟天 |  | nystose | O | O |  |
| Moutan Radicis Cortex | 목단피 | 牡丹皮 | ボタンピ | paeonol | O | O | O |
| Mume Flos | 매화 | 梅花 |  | chlorogenic acid | O |  |  |
| Mume Flos | 매화 | 梅花 |  | hyperin | O |  |  |
| Mume Flos | 매화 | 梅花 |  | isoquercitrin | O |  |  |
| Mume Fructus | 오매 | 乌梅 | ウバイ | citric acid | O | O | O |
| Myristicae Semen | 육두구 | 肉豆蔻 | ニクズク | dehydrodiisoeugenol | O | O | O |
| Nardostachyos Radix et Rhizoma | 감송향 | 甘松 |  | nardosinone | O | O | O |
| Nelumbinis Folium | 하엽 | 荷叶 |  | nuciferine | O | O | O |
| Nelumbinis Plumula | 연자심 | 莲子心 |  | liensinine | O | O | O |
| Notoginseng Radix et Rhizoma | 삼칠 | 三七 | サンシチニンジン | ginsenoside Rb1 | O | O | O |
| Notoginseng Radix et Rhizoma | 삼칠 | 三七 | サンシチニンジン | ginsenoside Rg1 | O | O | O |
| Notoginseng Radix et Rhizoma | 삼칠 | 三七 | サンシチニンジン | notoginsenoside R1 | O | O | O |
| Oroxyli Semen | 목호접 | 木蝴蝶 |  | oroxin B | O |  | O |
| Osterici seu Notopterygii Radix et Rhizoma | 강활 | 羌活 | キョウカツ | isoimperatorin | O | O | O |
| Osterici seu Notopterygii Radix et Rhizoma | 강활 | 羌活 | キョウカツ | notopterol | O | O | O |
| Paeoniae Radix | 작약 | 白芍 赤芍 | シャクヤク | paeoniflorin | O | O | O |
| Panacis Quinquefolii Radix | 서양삼 | 西洋参 |  | ginsenoside Rb1 | O | O | O |
| Panacis Quinquefolii Radix | 서양삼 | 西洋参 |  | ginsenoside Re | O | O | O |
| Panacis Quinquefolii Radix | 서양삼 | 西洋参 |  | ginsenoside Rg1 | O | O | O |
| Papaveris Pericarpium | 앵속각 | 罂粟壳 |  | morphine | O | O | O |
| Perillae Fructus | 자소자 | 紫苏子 | シソシ | rosmarinic acid | O |  | O |
| Persicae Semen | 도인 | 桃仁 | トウニン | amygdalin | O | O | O |
| Peucedani Radix | 전호 | 前胡 紫花前胡 | ゼンコ | nodakenin | O | O | O |
| Peucedani Radix | 전호 | 前胡 紫花前胡 | ゼンコ | praeruptorin A | O | O | O |
| Peucedani Radix | 전호 | 前胡 紫花前胡 | ゼンコ | praeruptorin B | O | O |  |
| Phellodendri Cortex | 황백 | 黄柏 关黄柏 | オウバク | berberine | O | O | O |
| Phellodendri Cortex | 황백 | 黄柏 关黄柏 | オウバク | berberine chloride | O |  |  |
| Phellodendri Cortex | 황백 | 黄柏 关黄柏 | オウバク | phellodendrine | O | O | O |
| Phyllanthi Fructus | 여감자 | 余甘子 |  | gallic acid | O | O | O |
| Physochlainae Radix | 화산삼 | 华山参 |  | hyoscyamine | O | O | O |
| Physochlainae Radix | 화산삼 | 华山参 |  | scopoletin |  | O |  |
| Phytolaccae Radix | 상륙 | 商陆 |  | esculentoside A | O | O | O |
| Picrorhizae Rhizoma | 호황련 | 胡黄连 |  | picroside I | O | O | O |
| Picrorhizae Rhizoma | 호황련 | 胡黄连 |  | picroside II | O | O | O |
| Pinelliae Tuber | 반하 | 半夏 | ハンゲ | succinic acid | O | O |  |
| Piperis Longi Fructus | 필발 | 荜茇 |  | piperine | O | O | O |
| Piperis Nigri Fructus | 후추 | 胡椒 |  | piperine | O |  | O |
| Plantaginis Herba | 차전초 | 车前草 | シャゼンソウ | plantamajoside | O | O |  |
| Plantaginis Semen | 차전자 | 车前子 | シャゼンシ | geniposidic acid | O | O |  |
| Plantaginis Semen | 차전자 | 车前子 | シャゼンシ | verbascoside | O |  |  |
| Platycodonis Radix | 길경 | 桔梗 | キキョウ | platycodin D | O | O | O |
| Pogostemonis Herba | 광곽향 | 广藿香 | カッコウ | patchouli alcohol | O | O | O |
| Polygoni Avicularis Herba | 편축 | 萹蓄 |  | myricitrin | O | O |  |
| Polygoni Cuspidati Rhizoma et Radix | 호장근 | 虎杖 |  | emodin | O | O | O |
| Polygoni Cuspidati Rhizoma et Radix | 호장근 | 虎杖 |  | polydatin | O | O | O |
| Polygoni Orientalis Fructus | 수홍화자 | 水红花子 |  | taxifolin | O | O |  |
| Polyporus | 저령 | 猪苓 | チョレイ | ergosterol | O | O | O |
| Ponciri Fructus Immaturus | 지실 | 枳实 | キジツ | synephrine | O |  | O |
| Potentillae Herba | 위릉채 | 委陵菜 |  | gallic acid |  | O | O |
| Prunellae Spica | 하고초 | 夏枯草 | カゴソウ | rosmarinic acid | O |  |  |
| Pruni Japonicae Semen | 욱리인 | 郁李仁 |  | amygdalin | O | O | O |
| Puerariae Radix | 갈근 | 葛根 | カッコン | puerarin | O | O | O |
| Pulsatillae Radix | 백두옹 | 白头翁 |  | anemoside B4 | O | O | O |
| Pyrolae Herba | 녹제초 | 鹿衔草 |  | monotropein | O |  | O |
| Quisqualis Fructus | 사군자 | 使君子 |  | trigonelline | O |  | O |
| Raphani Semen | 내복자 | 莱菔子 |  | sinapine thiocyanate | O | O | O |
| Rehmanniae Radix Preparata | 숙지황 | 熟地黄 |  | verbascoside | O | O |  |
| Rhapontici Radix | 누로 | 漏芦 |  | β-ecdysone | O | O | O |
| Rhei Radix et Rhizoma | 대황 | 大黄 | ダイオウ | aloe-emodin | O | O | O |
| Rhei Radix et Rhizoma | 대황 | 大黄 | ダイオウ | anthraquinone | O |  |  |
| Rhei Radix et Rhizoma | 대황 | 大黄 | ダイオウ | chrysophanol | O | O | O |
| Rhei Radix et Rhizoma | 대황 | 大黄 | ダイオウ | emodin | O | O | O |
| Rhei Radix et Rhizoma | 대황 | 大黄 | ダイオウ | physcion | O | O | O |
| Rhei Radix et Rhizoma | 대황 | 大黄 | ダイオウ | rhein | O | O | O |
| Rhododendri Daurici Folium | 만산홍 | 满山红 |  | farrerol | O | O | O |
| Rosae Chinensis Flos | 월계화 | 月季花 |  | hyperin | O |  | O |
| Rosae Chinensis Flos | 월계화 | 月季花 |  | isoquercitrin | O | O |  |
| Rubi Fructus | 복분자 | 覆盆子 |  | ellagic acid | O | O | O |
| Rubi Fructus | 복분자 | 覆盆子 |  | kaempferol 3-rutinoside | O | O | O |
| Rubiae Radix | 천초근 | 茜草 |  | mollugin | O | O | O |
| Rubiae Radix | 천초근 | 茜草 |  | purpurin | O | O | O |
| Salviae Miltiorrhizae Radix | 단삼 | 丹参 | タンジン | cryptotanshinone | O | O | O |
| Salviae Miltiorrhizae Radix | 단삼 | 丹参 | タンジン | salvianolic acid B | O | O | O |
| Salviae Miltiorrhizae Radix | 단삼 | 丹参 | タンジン | tanshinone I | O | O | O |
| Salviae Miltiorrhizae Radix | 단삼 | 丹参 | タンジン | tanshinone IIA | O | O | O |
| Saposhnikoviae Radix | 방풍 | 防风 | ボウフウ | 4'-O-β-D-glucosyl-5-O-methylvisamminol | O | O | O |
| Saposhnikoviae Radix | 방풍 | 防风 | ボウフウ | prim-O-glucosylcimifugin | O | O | O |
| Sarcandrae Herba | 종절풍 | 肿节风 |  | isofraxidin | O | O | O |
| Sarcandrae Herba | 종절풍 | 肿节风 |  | rosmarinic acid | O |  |  |
| Saururi Herba | 삼백초 | 三白草 |  | sauchinone | O | O | O |
| Schisandrae Fructus | 오미자 | 五味子 | ゴミシ | schisandrin | O | O | O |
| Schizonepetae Spica | 형개 | 荆芥 荆芥穗 | ケイガイ | menthone | O | O | O |
| Scrophulariae Radix | 현삼 | 玄参 | ゲンジン | harpagide | O | O | O |
| Scrophulariae Radix | 현삼 | 玄参 | ゲンジン | harpagoside | O | O | O |
| Scutellariae Barbatae Herba | 반지련 | 半枝莲 |  | scutellarin | O | O | O |
| Scutellariae Radix | 황금 | 黄芩 | オウゴン | baicalin | O | O | O |
| Sedi Herba | 수분초 | 垂盆草 |  | isorhamnetin | O | O | O |
| Sedi Herba | 수분초 | 垂盆草 |  | kaempferol | O |  |  |
| Sedi Herba | 수분초 | 垂盆草 |  | quercetin | O | O | O |
| Selaginellae Herba | 권백 | 卷柏 |  | amentoflavone | O | O | O |
| Sennae Folium | 번사엽 | 番泻叶 | センナ | sennoside A | O | O | O |
| Sennae Folium | 번사엽 | 番泻叶 | センナ | sennoside B | O | O | O |
| Silybi Fructus | 수비계 | 水飞蓟 |  | silibinin | O | O | O |
| Sinomeni Caulis et Rhizoma | 방기 | 防己 青风藤 | ボウイ | fangchinoline | O | O | O |
| Sinomeni Caulis et Rhizoma | 방기 | 防己 青风藤 | ボウイ | tetrandrine | O | O | O |
| Siphonostegiae Herba | 북유기노 | 北刘寄奴 |  | luteolin |  | O | O |
| Siphonostegiae Herba | 북유기노 | 北刘寄奴 |  | verbascoside |  |  |  |
| Siraitiae Fructus | 나한과 | 罗汉果 |  | mogroside V | O | O |  |
| Smilacis Rhizoma | 토복령 | 土茯苓 菝葜 | サンキライ | astilbin | O | O | O |
| Sophorae Flos | 괴화 | 槐花 | カイカ | rutin | O |  | O |
| Sophorae Fructus | 괴각 | 槐角 |  | sophoricoside | O | O | O |
| Sophorae Radix | 고삼 | 苦参 | クジン | matrine | O | O | O |
| Sophorae Radix | 고삼 | 苦参 | クジン | oxymatrine | O | O | O |
| Sophorae Tonkinensis Radix et Rhizoma | 산두근 | 山豆根 | サンズコン | matrine | O | O | O |
| Sophorae Tonkinensis Radix et Rhizoma | 산두근 | 山豆根 | サンズコン | oxymatrine | O |  | O |
| Strychni Semen | 마전자 | 马钱子 | ホミカ | brucine | O | O | O |
| Strychni Semen | 마전자 | 马钱子 | ホミカ | strychnine | O |  | O |
| Swertiae Mileensis Herba | 청엽담 | 青叶胆 |  | swertiamarine | O | O |  |
| Syzygii Flos | 정향 | 丁香 | チョウジ | eugenol | O | O | O |
| Thujae Orientalis Folium | 측백엽 | 侧柏叶 |  | quercitrin | O | O | O |
| Tinosporae Radix | 금과람 | 金果榄 |  | columbin | O | O | O |
| Trachelospermi Caulis | 낙석등 | 络石藤 |  | tracheloside | O | O | O |
| Trigonellae Semen | 호로파 | 胡芦巴 |  | trigonelline | O |  | O |
| Typhae Pollen | 포황 | 蒲黄 |  | isorhamnetin 3-O-neohesperidin | O |  | O |
| Typhae Pollen | 포황 | 蒲黄 |  | typhaneoside | O |  | O |
| Verbenae Herba | 마편초 | 马鞭草 |  | oleanolic acid | O | O |  |
| Verbenae Herba | 마편초 | 马鞭草 |  | ursolic acid | O | O | O |
| Visci Ramulus et Folium | 곡기생 | 槲寄生 |  | syringin | O | O |  |
| Viticis Fructus | 만형자 | 蔓荆子 | マンケイシ | vitexicarpin | O | O | O |
| Xanthii Fructus | 창이자 | 苍耳子 |  | carboxyatractyloside | O | O | O |
| Xanthii Fructus | 창이자 | 苍耳子 |  | chlorogenic acid | O | O | O |
| Zanthoxyli Radix | 양면침 | 两面针 |  | nitidine chloride | O | O | O |
| Zingiberis Rhizoma | 건강 | 干姜 | カンキョウ | 6-gingerol | O | O | O |
| Zingiberis Rhizoma Recens | 생강 | 生姜 | ショウキョウ | 10-gingerol | O | O | O |
| Zingiberis Rhizoma Recens | 생강 | 生姜 | ショウキョウ | 6-gingerol | O | O | O |
| Zingiberis Rhizoma Recens | 생강 | 生姜 | ショウキョウ | 8-gingerol | O | O |  |
| Zizyphi Semen | 산조인 | 酸枣仁 | サンソウニン | jujuboside A | O | O | O |
| Zizyphi Semen | 산조인 | 酸枣仁 | サンソウニン | spinosin | O | O | O |
